# *ARL6IP1* gene delivery reduces neuroinflammation and neurodegenerative pathology in hereditary spastic paraplegia model

**DOI:** 10.1084/jem.20230367

**Published:** 2023-11-07

**Authors:** Jung Hwa Lim, Hyun Mi Kang, Dae Hun Kim, Bohyeon Jeong, Da Yong Lee, Jae-Ran Lee, Jeong Yeob Baek, Hyun-Soo Cho, Mi-Young Son, Dae Soo Kim, Nam-Soon Kim, Cho-Rok Jung

**Affiliations:** 1 Korea Research Institute of Bioscience and Biotechnology, Daejeon, Republic of Korea; 2 Department of Functional Genomics, Korea University of Science and Technology, Daejeon, Republic of Korea

## Abstract

ARL6IP1 is implicated in hereditary spastic paraplegia (HSP), but the specific pathogenic mechanism leading to neurodegeneration has not been elucidated. Here, we clarified the molecular mechanism of ARL6IP1 in HSP using in vitro and in vivo models. The *Arl6ip1* knockout (KO) mouse model was generated to represent the clinically involved frameshift mutations and mimicked the HSP phenotypes. Notably, in vivo brain histopathological analysis revealed demyelination of the axon and neuroinflammation in the white matter, including the corticospinal tract. In in vitro experiments, *ARL6IP1* silencing caused cell death during neuronal differentiation and mitochondrial dysfunction by dysregulated autophagy. ARL6IP1 localized on mitochondria-associated membranes (MAMs) to maintain endoplasmic reticulum and mitochondrial homeostasis via direct interaction with LC3B and BCl2L13. ARL6IP1 played a crucial role in connecting the endoplasmic reticulum and mitochondria as a member of MAMs. *ARL6IP1* gene therapy reduced HSP phenotypes and restored pathophysiological changes in the *Arl6ip1* KO model. Our results established that *ARL6IP1* could be a potential target for HSP gene therapy.

## Teaser

We established an *Arl6ip1*^*−/−*^ mouse model mimicking HSP with severe spastic paralysis and gait abnormalities. Neuroinflammation was identified as a key pathophysiological change. AAV9-ARL6IP1 delivery reduced limb paraplegia and gait abnormality, making ARL6IP1 a potential target for HSP gene therapy.

## Graphical Abstract

**Figure:**
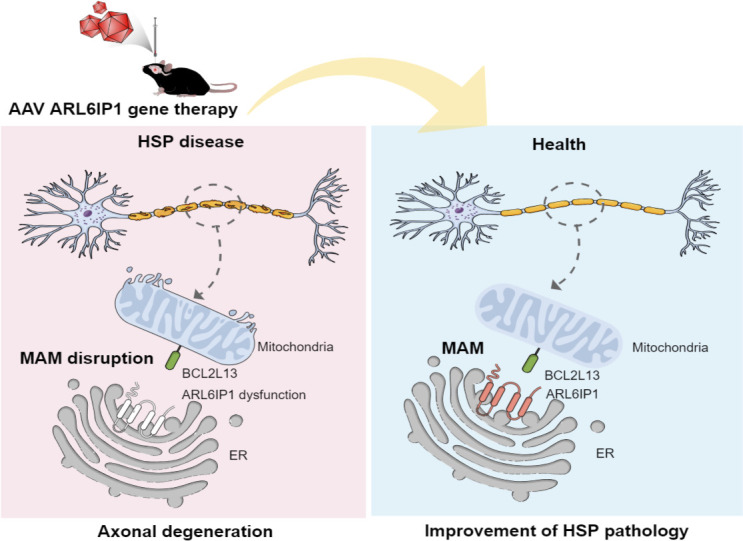

## Introduction

Hereditary spastic paraplegia (HSP), a genetically and symptomatically heterogeneous neurodegenerative disease, is classified as either uncomplicated (pure; impairment limited to lower extremity spasticity and weakness) or complicated (accompanied by other neuronal symptoms such as distal peripheral neuropathy and cognitive impairments or seizures) types (Lo Giudice et al., 2014; Shribman et al., 2019). To date, 72 different HSP disease loci have been identified and 55 spastic paraplegia (SPG) genes have been cloned (Tesson et al., 2015; Lallemant-Dudek et al., 2021). SPG proteins are involved in HSP pathogenesis via different molecular pathways, including axonal transport, lipid metabolism, and mitochondrial dysfunction. Several SPG proteins, such as SPG4, SPG3A, and receptor expression-enhancing proteins (e.g., REEP1 [SPG31], REEP2, and SPG72) shape the smooth ER network (Hübner and Kurth, 2014; Blackstone, 2012; Voeltz et al., 2006; Park et al., 2010; Chang et al., 2013; Gumeni et al., 2021). Recently, ARL6IP1, another ER-shaping protein, has been identified as SPG61 using whole-exome sequencing in a consanguineous family with HSP (Novarino et al., 2014). ARL6IP1 possesses hairpin-loop domains involving ER structural proteins, which localize to smooth ER tubules, modulating their morphology by increasing the curvature to form highly curved smooth ER tubules (Yamamoto et al., 2014). The ER is a cell organelle with the largest intracellular membrane architecture, controlling protein and lipid syntheses, while ensuring their quality and organelle communications (Hu et al., 2008; Park and Blackstone, 2010; Walter and Ron, 2011; Schuck et al., 2009). Constant ER turnover and modulation are required to meet different cellular requirements with autophagy (Borgese et al., 2006; Khaminets et al., 2015). The ER and mitochondria are closely linked via mitochondria-associated membranes (MAMs), which play an important role in maintaining healthy cellular functions via molecule exchange through processes such as calcium homeostasis, autophagy, lipid metabolism, cellular metabolic homeostasis, and neuroinflammation (Missiroli et al., 2018; Perrone et al., 2020; Vance, 2015). Particularly, the exchange of metabolites and signaling molecules through MAMs in neurons is widely implicated in neurodegenerative disorders, such as Alzheimer’s disease (Schon and Area-Gomez, 2013), amyotrophic lateral sclerosis (Bernard-Marissal et al., 2015), Parkinson’s disease (Guardia-Laguarta et al., 2014), and other axonal degeneration diseases, including HSP and Charcot‒Marie‒Tooth disease (Krols et al., 2016). Here, we investigated the pathological role of ARL6IP1 in HSP. Specifically, we elucidated the crucial underlying role of ARL6IP1 in organelle homeostasis through ER and mitochondrial connection, regulated by mitophagy, and in neuroinflammation pathology using *Arl6ip1* KO versus *Arl6ip1* wild-type (WT) mouse models. We also investigated the body morphology and neurodegenerative phenotypes present in the *Arl6ip1* KO mouse model to examine the role of ARL6IP1 in neuronal homeostasis. Furthermore, the therapeutic effect of targeted gene delivery was assessed for HSP gene therapy development.

## Results

### Limb abnormalities and histopathological changes observed in human HSP are replicated in the *Arl6ip1*^*−/−*^ mouse model

**Figure 1.**
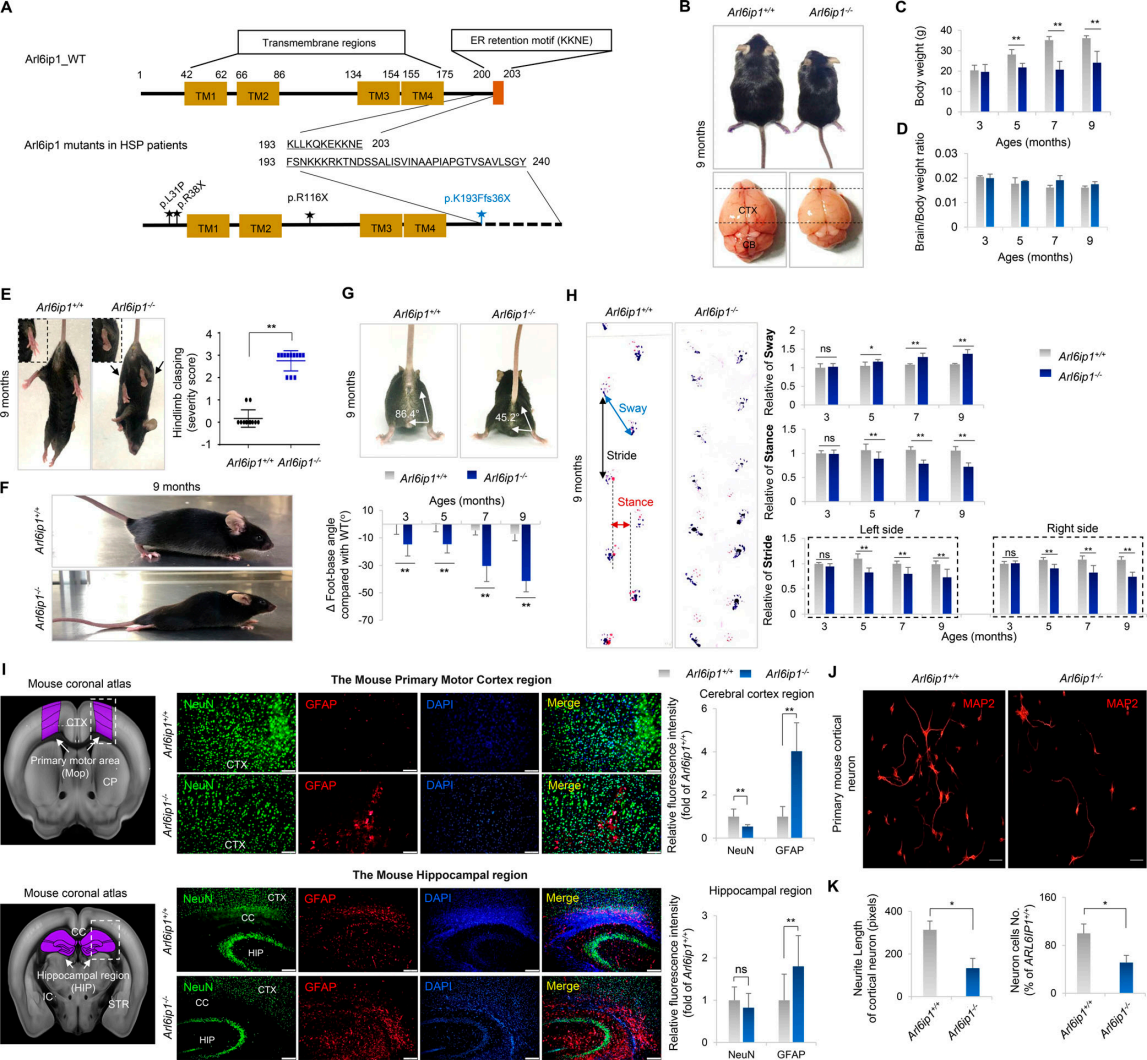
**Behavioral abnormalities and brain histopathology in an *Arl6ip1***^***−/−***^ **mouse model of HSP. (A)** ARL6IP1 protein structure. Locations of mutations previously identified in patients with HSP. **(B)** Representative body and brain size of *Arl6ip1*^*–/– *^and *Arl6ip1*^*+/+*^ mice at 9 mo. CTX, cerebral cortex; CB, cerebellum. **(C and D)** Measurement of body (C) and brain/body weight ratio (D) change over time of *Arl6ip1*^*−/−*^ and *Arl6ip1*^*+/+*^ mice. Data are presented as mean ± SD of *n* = 10 mice/group. **(E)** Representative image of abnormal limb reflexes in 9-mo-old *Arl6ip1*^*−/−*^ and *Arl6ip1*^*+/+*^ mice. **(F)** Abnormal hindlimb posture of *Arl6ip1*^*−/−*^ and *Arl6ip1*^*+/+*^ mice. **(G)** Representative image (top) and measurement of foot-base angle (bottom) of *Arl6ip1*^*−/−*^ and *Arl6ip1*^*+/+*^ mice. Data are presented as mean ± SD of *n* = 10 mice/group. **(H)** Representative image of disturbed footprint pattern in *Arl6ip1*^*−/−*^ and *Arl6ip1*^*+/+*^ mice (left). Forelimbs are marked in red and hindlimbs in blue. Stride, stance, and sway lengths were calculated from footprint patterns of *Arl6ip1*^*−/−*^ and *Arl6ip1*^*+/+*^ mice (right). Data represent mean ± SD of *n* = 10 mice/group. **(I)** Mouse coronal section image from Allen Adult Mouse Brain Atlas (http://atlas.brain-map.org) at the same slice position as primary motor cortex and hippocampus regions (left, marked in purple). Representative images of immunofluorescence staining of NeuN and GFAP in the primary motor cortex (top) and hippocampus (down) regions of *Arl6ip1*^*−/−*^ and *Arl6ip1*^*+/+*^ mice (200×, scale bar: 100 μm). **(J)** Representative immunofluorescence image showing MAP2 expression in primary cortical neurons from *Arl6ip1*^*−/−*^ and *Arl6ip1*^*+/+*^ mice (400×, scale bar: 50 μm). **(K)** Neurite length and neuron cell number in WT and *Arl6ip1*-deficient cortical neurons. Data represent mean ± SD of triplicates and are analyzed using Student’s *t* test. *P < 0.05; **P < 0.01; ns, not significant. Data represent averages of three independent biological replicates with two technical replicates each.

**Figure S1.**
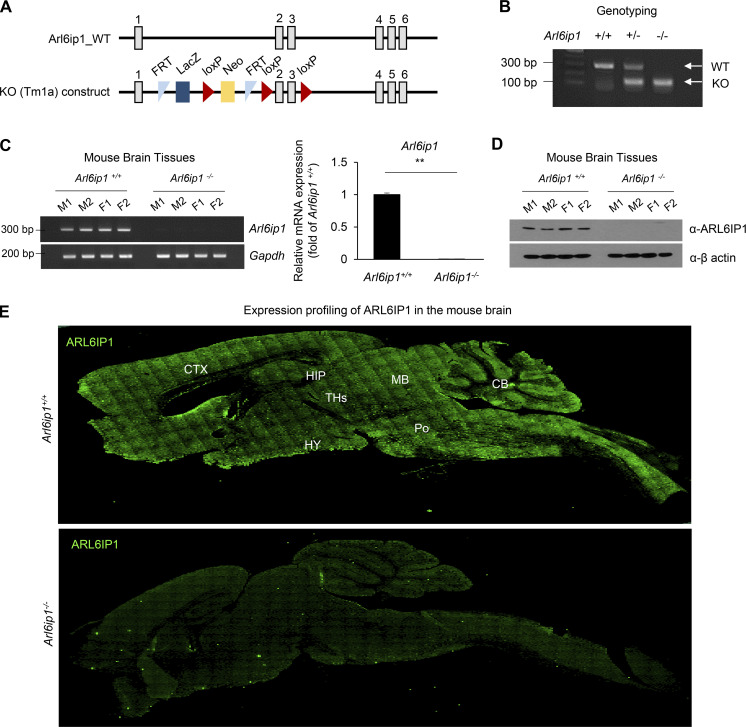
**Information of *Arl6ip1***^***−/−***^**mice. (A)** The WT allele of *Arl6ip1* (top) and the targeted locus of *Arl6ip1* (bottom) is shown in the schematic diagram. The L1L2_Bact_P cassette was inserted at position 118127554 of Chromosome 7 upstream of the critical exon(s) (Build GRCm38). The cassette is composed of an FRT site followed by lacZ sequence and a loxP site. This first loxP site is followed by a neomycin resistance gene under the control of the human β-actin promoter, SV40 polyA, a second FRT site and a second loxP site. A third loxP site is inserted downstream of the targeted exon(s) at position 118125888. Further information on targeting strategies used for this and other IKMC alleles can be found at http://www.informatics.jax.org/mgihome/nomen/IKMC_schematics.shtml (J:157065). Gray boxes represent exons and black lines represent introns. **(B)** Mouse genotyping by PCR in WT (*Arl6ip1*^*+/+*^), heterozygous (*Arl6ip1*^*+/−*^), and homozygous *Arl6ip1* KO (*Arl6ip1*^*−/−*^) mice. **(C)** RT-PCR (left) and real-time PCR (right) analysis to confirm the loss of mRNA level in *Arl6ip1*^*−/−*^ brain tissues. *Arl6ip1*^*+/+*^ was used as a positive control for evaluating the ARL6IP1 expression (male and female mice aged 9 mo, total *n* = 4/group). **(D)** The protein level of ARL6IP1 in brain tissue lysates from *Arl6ip1*^*−/−*^ and *Arl6ip1*^*+/+*^ mice. **(E)** Immunofluorescence image of ARL6IP1 level in the brain tissues from *Arl6ip1*^*−/−*^ and *Arl6ip1*^*+/+*^ mice at 6 mo (20×; scale bars: 1 mm). Data are represented by three independent biological replicates, the mean and error bars represent the SEM, and the two-tailed unpaired *t* test. **P < 0.01. CTX, cortex; HIPP, hippocampus; THs, thalamus; HY, hypothalamus; MB, midbrain; Po, pons; CB, cerebellum. Source data are available for this figure: SourceData FS1.

*ARL6IP1* mutations are strongly implicated in HSP; five families have been reported with complex forms of autosomal recessive (AR)-HSP (Table S1). Among them, an *ARL6IP1* mutation (“AAAC” at position c.576_579, leading to a frameshift) was reported in two families at the same locus (Novarino et al., 2014; Nizon et al., 2018). This non-stop mutation causes the continuous translation of the mRNA into the untranslated region, which is consequently regulated by transcription quality control by the non-stop decay mechanism (Garneau et al., 2007). A schematic diagram of the *ARL6IP1* mutation reported in patients with HSP is illustrated in Fig. 1 A. To clarify the physiological significance of *ARL6IP1* in vivo, *Arl6ip1* KO homozygote (*Arl6ip1*^*−/−*^) mice were generated by *Arl6ip1* heterozygotes (*Arl6ip1*^*+/−*^) and identified by genotyping. Genotyping of KO alleles in mice is usually performed via PCR using specifically designed primers. For the genotyping of pups, toes were used for genomic DNA extraction and amplification. *ARL6IP1* transcripts and protein were not detected in the brain tissues of *Arl6ip1*^*−/−*^ mice (Fig. S1, A–D). ARL6IP1 was broadly expressed in the WT (*Arl6ip1*^*+/+*^) mouse brain, including the olfactory bulb, cortex, hippocampus, and cerebellum, but it was not detected in the *Arl6ip1*^*−/−*^ mice (Fig. S1 E). They showed significant age-dependent differences progressively in body and brain weight compared with *Arl6ip1*^*+/+*^ *mice* (Fig. 1, B‒D). At 9 mo of age, *Arl6ip1*^*−/−*^ mice developed an abnormal hindlimb reflex and posture where the hindlimbs contracted toward the trunk, as opposed to a normal extension of legs in *Arl6ip1*^*+/+*^ mice when the hindlimbs were lifted by the tail (Fig. 1, E and F). To evaluate the locomotor function, *Arl6ip1*^*−/−*^ and *Arl6ip1*^*+/+*^ mice were compared for progressive gait disorder and motor deficits by measuring the foot-base angle. Significant differences were identified from 3 mo of age. At 9 mo of age, the foot-base angle of *Arl6ip1*^*−/−*^ mice severely decreased to ∼45.2° compared to ∼86.4° of *Arl6ip1*^*+/+*^ mice (Fig. 1 G). Footprint analysis of *Arl6ip1*^*−/−*^ mice showed a significant decrease in the hindlimb stride and stance length and increased relative hindlimb sway length compared with those seen in *Arl6ip1*^*+/+*^ mice from 5 mo of age (Fig. 1 H and Videos 1 and 2). Overall, the *Arl6ip1*^*−/−*^ mice exhibited initial mild phenotypic manifestations at 3 mo, progressively exacerbating at 5 and 7 mo, culminating in pronounced symptoms by 9 mo, for which visual data were presented. Furthermore, longitudinal changes were quantified and documented over time.

**Video 1.** **The abnormal gait of 9-mo-old *Arl6ip1***^***−/−***^ **mice.** In 9-mo-old *Arl6ip1*^*−/−*^ mice, exhibiting HSP disease phenotypes such as hindlimb muscle weakness and spasticity, abnormalities in mouse locomotor activity and gait were observed. Video is presented in real-time.

**Video 2.** **The normal gait of 9-mo-old *Arl6ip1***^***+/+***^ **mice.** In 9-mo-old *Arl6ip1*^*+/+*^ mice showed normal gait. Video is presented in real-time.

**Figure S2.**
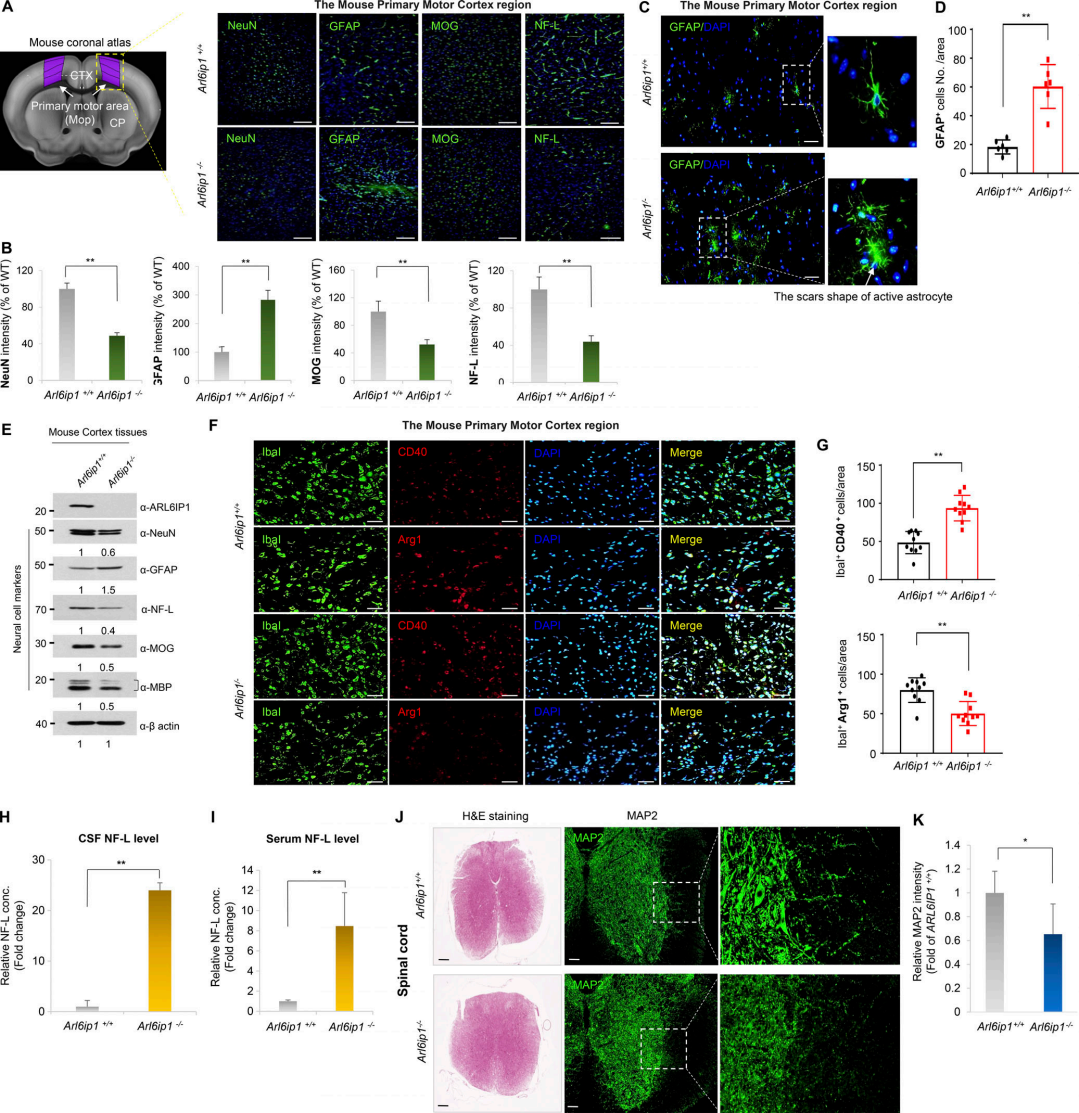
**Neuropathological changes in the corticospinal tract of *Arl6ip1***^***−/−***^ **mice. (A and B)** Representative immunofluorescence images and quantification of NeuN, GFAP, MOG, and NF-L level in the primary motor cortex region from *Arl6ip1*^*−/−*^ and *Arl6ip1*^*+/+*^ mice at 6 mo (left, 200×; scale bars: 100 μm). **(C and D)** Representative immunofluorescence images and quantification of scar-forming astrocytes with GFAP in the mouse primary motor cortex region. **(E)** Protein levels of neuronal and glial markers in cortex tissues. **(F and G)** Representative immunofluorescence images and quantification for CD40-Iba1 microglia, and Arg-1-Iba1 microglia in the mouse primary motor cortex region. **(H and I)** Concentrations of NF-L in the CSF and serum in *Arl6ip1*^*−/−*^ and *Arl6ip1*^*+/+*^ mice at 9 mo (male, *n* = 8 mice/group). **(J)** Representative image of H&E and MAP2 fluorescence staining in the spinal cord from *Arl6ip1*^*−/−*^ and *Arl6ip1*^*+/+*^ mice at 6 mo (40×; scale bars: 500 μm; 200×; scale bars: 100 μm). **(K)** Quantification of immunofluorescence staining. Data are represented with the average of three independent biological replicates, and the mean and error bars represent the SEM, and the two-tailed unpaired *t* test. *P < 0.05, **P < 0.01. Source data are available for this figure: SourceData FS2.

HSP causes degeneration of the distal end of the corticospinal cord; hence, the most common neuroimaging finding is atrophy of the spinal cord (Hedera et al., 2005). To determine the consequences of *ARL6IP1* deficiency in the central nervous system (CNS) of *Arl6ip1*^*−/−*^ mice, we assessed astrocyte-, oligodendrocyte-, and neuron-specific protein levels via immunofluorescence and western blot analyses of the cerebral cortex of *Arl6ip1*^*−/−*^ and *Arl6ip1*^*+/+*^ mice. The levels were decreased in neurons (NeuN and neurofilament light chain; NF-L) and mature oligodendrocytes (myelin oligodendrocyte glycoprotein; MOG), while the expression of GFAP, an active astrocyte marker, was increased in *Arl6ip1*^*−/−*^ mice (Fig. 1 I and Fig. S2, A–E). To verify the disease-specific pathophysiology of the *ARL6IP1* mutation in primary mouse cortical neurons from *Arl6ip1*^*−/−*^ and *Arl6ip1*^*+/+*^ mice, the expression of MAP2, a neuronal differentiation marker, was reduced compared with that in *Arl6ip1*^*+/+*^ mice. *Arl6ip1*^*−/−*^ mice significantly reduced neuronal cell counts and neurite outgrowth compared with the corresponding counterparts in *Arl6ip1*^*+/+*^ mice (Fig. 1, J and K).

### *Arl6ip1* depletion is implicated in demyelination seen in the HSP mouse model

**Figure 2.**
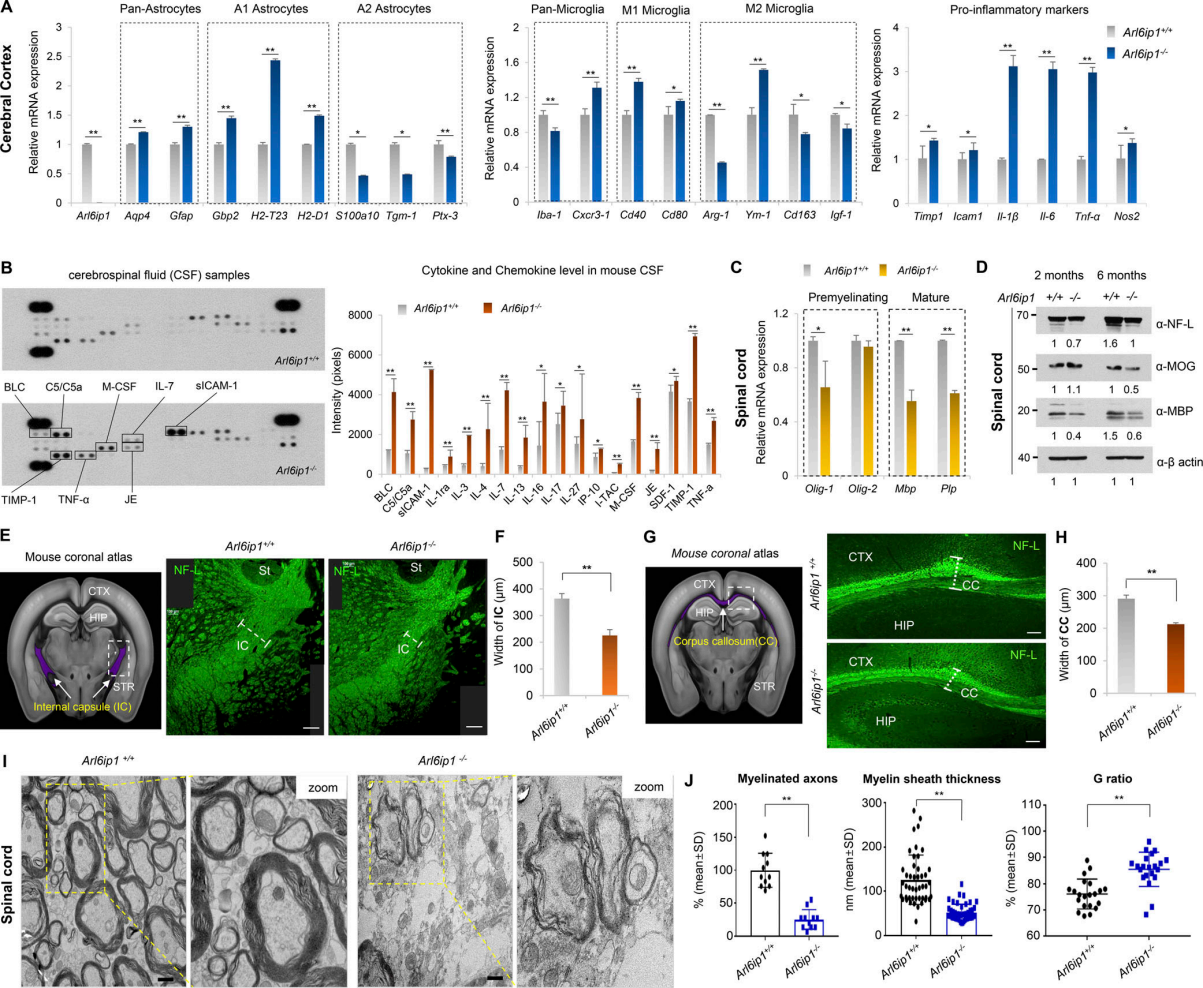
**Abnormal myelination is associated with neuroinflammation in an *Arl6ip1***^***−/−***^ **mouse model of HSP. (A)** mRNA levels of glial or pro-inflammatory marker genes in the cortex from *Arl6ip1*^*−/−*^ and *Arl6ip1*^*+/+*^ mice at 6 mo. Data represent mean ± SEM of *n* = 6 mice/group. **(B)** Determination of relative levels of mouse cytokines and chemokines using a proteome profiler mouse cytokine array kit in mouse CSF samples at 6 mo. Data represent mean ± SD of *n* = 8 mice/group. **(C)** mRNA levels of CNS myelin-related genes in the spinal cord from *Arl6ip1*^*−/−*^ and *Arl6ip1*^*+/+*^ mice at 6 mo. Data represent mean ± SEM of *n* = 6 mice/group. **(D)** Protein levels of NF-L, MOG, and MBP from pooled lysates in the spinal cord of *Arl6ip1*^*−/−*^ and *Arl6ip1*^*+/+*^ mice at 2 and 6 mo, as assessed via western blot (*n* = 3 mice/group). **(E)** Mouse coronal section image from Allen Adult Mouse Brain Atlas (http://atlas.brain-map.org) showing internal capsule area (left, marked in purple). Representative NF-L fluorescence staining (right, 200×; scale bar: 100 μm) images in the internal capsule of brain tissues from *Arl6ip1*^*−/−*^ and *Arl6ip1*^*+/+*^ mice at 6 mo. CTX, cerebral cortex; HIP, hippocampus; STR, striatum; St, stria terminals; IC, internal capsule. **(F)** Internal capsule width (myelin thickness) was measured using ImageJ v1.57. Data represent mean ± SD of *n* = 5 mice/group and are analyzed using Student’s *t* test. **(G)** Mouse coronal section image from Allen Adult Mouse Brain Atlas showing the corpus callosum area (left, marked in purple). Representative NF-L fluorescence staining images in white matter regions of brain tissues from *Arl6ip1*^*−/−*^ and *Arl6ip1*^*+/+*^ mice at 6 mo (right, 200×; scale bar: 100 μm) CTX, cerebral cortex; HIP, hippocampus; CC, corpus callosum; STR, striatum. **(H)** Quantification of immunofluorescence staining. Data represent mean ± SD of *n* = 5 mice/group and are analyzed using Student’s *t* test. **(I)** Representative TEM images of spinal cord white matter in *Arl6ip1*^*−/−*^ and *Arl6ip1*^*+/+*^ mice at 6 mo (*n* = 4/group, 15,000×; scale bar: 800 nm). **(J)** Quantitative data of the percentage of myelinated axons, G ratio, and myelin sheath thickness. G ratio = % axon diameter/diameter of the axon with a myelin sheath. Data represent mean ± SD and are calculated in GraphPad Prism 7.0. *P < 0.05; **P < 0.01; ns, not significant. Data represent averages of three independent biological replicates with two technical replicates for each. Source data are available for this figure: SourceData F2.

Glial cells in the CNS of adult mammals, including astrocytes, oligodendrocytes, and microglia, play many roles, such as physical support and protection of neurons, maintenance of homeostasis, formation of myelination, nerve signal propagation, and responses to neural injury (Herculano-Houzel, 2014; Zuchero and Barres, 2015). The mRNA pattern of glial cells was analyzed in the cerebral cortex of *Arl6ip1*^*−/−*^ and *Arl6ip1*^*+/+*^ mice. We found that the mRNA levels of pan-astrocytes and A1-reactive astrocytes were increased, whereas those of A2-reactive astrocytes were decreased in *Arl6ip1*^*−/−*^ mice (Fig. 2 A, left). Astrocytes can respond to CNS damage and exist as quiescent astrocytes in the normal CNS. A1-reactive astrocytes upregulate neurotoxins and proinflammatory cytokine levels, which can induce neuronal cell death, whereas A2-reactive astrocytes activate neurotrophic factors and anti-inflammatory cytokines for neuron survival. In CNS injury, microglia are activated as either M1 or M2, wherein M1 microglia release inflammatory mediators that induce inflammation and neurotoxicity and M2 microglia release anti-inflammatory and neuroprotective mediators. In *Arl6ip1*^*−/−*^ mice, the mRNA levels of *Cxcr3-1*, *Cd40*, and *Cd80* (M1 microglia markers) were increased, whereas those of *Arg-1*, *Cd163*, and *Igf-1* (M2 microglia markers) were decreased. However, the mRNA level of *Ym-1*, an M2 marker, was significantly increased (Fig. 2 A, middle panel). Also, a change of microglia M1/M2 polarization, a hallmark of neurodegenerative diseases, was observed in *Arl6ip1*^*−/−*^ mice (Fig. S2, F and G). Increased mRNA levels of proinflammatory cytokines and chemokines were also confirmed in *Arl6ip1*^*−/−*^ mice (Fig. 2 A, right). Inflammatory mediators are released by M1 microglia, which can activate A1 neurotoxic astrocytes and further induce neuroinflammation and demyelination. Moreover, oligodendrocytes generate and maintain the myelin sheath throughout the CNS; a typical pathological feature of neurodegenerative diseases is progressive axonal demyelination and degeneration. The cerebrospinal fluid (CSF) from *Arl6ip1*^*−/−*^ or *Arl6ip1*^*+/+*^ mice were analyzed to measure microglia and astrocyte activation, neuroinflammation, and cerebrovascular changes. BLC, C5/C5α, M-CSF, IL-7, sICAM-1, TIMP-1, TNF-α, and JE protein levels were considerably increased in the CSF from *Arl6ip1*^*−/−*^ mice (Fig. 2 B). The mRNA levels of oligodendrocyte precursor and differentiation markers were remarkably reduced in the spinal cord tissues of *Arl6ip1*^*−/−*^ mice than in those of *Arl6ip1*^*+/+*^ mice (Fig. 2 C). In addition, protein levels of NF-L as an axon marker of neurons, MOG, and myelin basic protein (MBP) as a marker of mature oligodendrocytes were reduced in the spinal cord tissues of *Arl6ip1*^*−/−*^ mice (Fig. 2 D). Radiological findings of patients with HSP are non-specific, including mild-to-moderate brain atrophy, thinning of the corpus callosum (CC), non-specific white matter lesions in the cerebral hemispheres, abnormal T2 high signal intensity in the posterior limb of the internal capsules, and atrophy of the spinal cord (Hourani et al., 2009). NFs have been identified as biomarkers for the clinical evaluation of neurodegeneration as a key protein contributing to the cytoskeleton of myelinated axons (Zucchi et al., 2020). We confirmed the reduction of myelinated axons in the internal capsule (IC), a major part of the corticospinal tract, of *Arl6ip1*^*−/−*^ mice by NF-L staining (Fig. 2, E and F). Similarly, a thin CC, a representative phenotype of complex AR-HSPs, was observed in *Arl6ip1*^*−/−*^ mice (Fig. 2, G and H). NFs were detected in the CSF and peripheral blood as a result of axonal degeneration. Markedly, increased NF-L concentrations were detected in the CSF and serum samples of *Arl6ip1*^*−/−*^ mice, confirming that *Arl6ip1*^*−/−*^ mice exhibited significant axonal degeneration compared with *Arl6ip1*^*+/+*^ mice (Fig. S2, H and I). In addition, the MAP2-positive signal intensity was low in the spinal cord of *Arl6ip1*^*−/−*^ mice, indicating a reduction of dendritic arborization (Fig. S2, J and K). TEM analysis revealed abnormal myelination, such as fewer and more thinly myelinated nerve fibers in the spinal cord of *Arl6ip1*^*−/−*^ mice (Fig. 2 I). The thickness of the myelinated axon was measured; a significant reduction was revealed in *Arl6ip1-*KO mice (Fig. 2 J). Thus, *Arl6ip1*^*−/−*^ mice had histopathophysiological features such as neuronal loss within the cortical spinal tract and thinning of the CC and exhibited a disease-specific phenotype with progressive weakness and stiffness of the hindlimbs.

### *Arl6ip1* deficiency promotes oxidative stress–induced apoptosis in mouse embryonic fibroblasts in vitro

**Figure 3.**
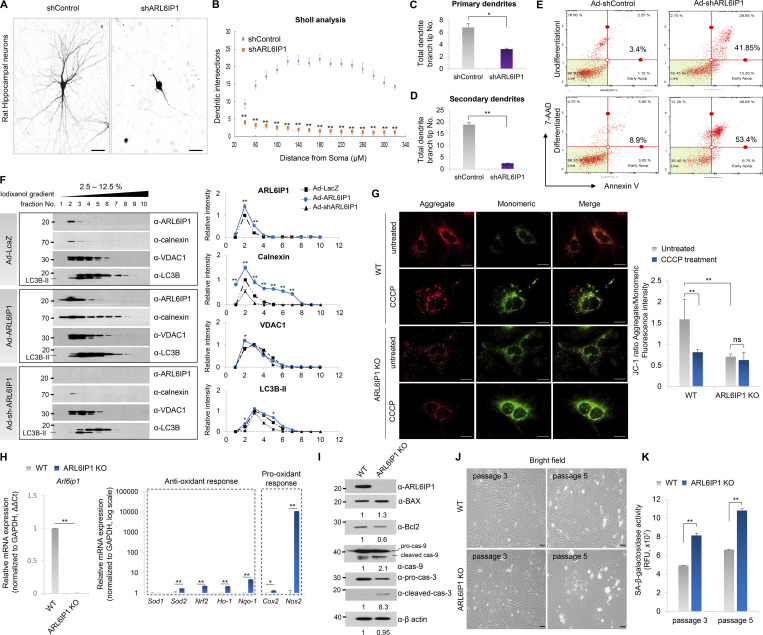
**Induction of oxidative stress–induced cellular senescence by ARL6IP1 deficiency. (A)** Representative dendritic arborization of primary rat hippocampal neurons after transfection with ARL6IP1-shRNA (shARL6IP1) and scramble shRNA (shControl) plasmids (600×; scale bar: 20 μm). **(B)** Sholl profiles show that dendritic arborization was attenuated by transfection of shARL6IP1. Means ± SEM of data from shControl and shARL6IP1 are shown (*n* = 10/group). **(C and D)** Numbers of primary and secondary dendrites were analyzed. Means ± SEM of data from shControl and shARL6IP1 neurons are shown (*n* = 10/group) and are analyzed using Student’s *t* test. **(E)** ReNcell CX was transduced with 100 MOI of adenovirus expressing ARL6IP1 shRNA (Ad-shARL6IP1) or scramble shRNA (Ad-shControl) 2 d after incubation in differentiation media (differentiated), and apoptotic cell death was assessed by Annexin V staining using MUSE cell analyzer. **(F)** Iodixanol density gradient analysis of ReNcell CX transduced with 100 MOI of adenovirus expressing ARL6IP1 (Ad-ARL6IP1) and Ad-shARL6IP1 (left). Quantitative data of intensity values of each protein using ImageJ v1.57 (right). **(G)** MMP was determined fluorometrically with JC-1 probe in *ARL6IP1* WT or KO MEFs at three passages (left, 600×; scale bar: 20 μm). The ratio of red to green JC-1 fluorescence intensity was measured using a fluorescence microplate reader (right, green fluorescent JC-1 monomers, Ex/Em = 485/529 nm; red fluorescent JC-1 aggregates, Ex/Em = 485/590 nm). Data represent mean ± SD of triplicates and are analyzed using Student’s *t* test. **(H)** mRNA level of ROS-related genes quantified using RT-qPCR in *ARL6IP1* WT and KO MEFs at three passages. Data represent mean ± SEM of triplicates. **(I)** Protein levels of apoptosis-related genes from *ARL6IP1* WT and KO MEFs at three passages. **(J)** Representative bright field images of passage 3 and 5 cells are shown in *ARL6IP1* WT and KO MEFs (200×; scale bar: 100 μm). **(K)** SA-β-Gal activity was measured in *ARL6IP1* WT and KO MEFs at passages 3 and 5 using a fluorescence microplate reader (Ex/Em = 360/465 nm). Data represent mean values of triplicates ± SD. *P < 0.05; **P < 0.01; ns, not significant. Data represent averages of three independent biological replicates. Source data are available for this figure: SourceData F3.

The effect of shRNA-ARL6IP1 was transiently tested in primary rat hippocampal neuron cells using the Sholl assay. ARL6IP1 depletion induced significantly shortened primary and secondary dendrites compared with those in the control (Fig. 3, A–D). In ReNcell CX, an immortalized human neural progenitor cell line transduced with Ad-shARL6IP1, the total apoptotic cell population, was significantly increased from 3.4% to 41.85% by *ARL6IP1* silencing, while the total apoptosis rate was increased from 8.9% to 53.4% in neuronal differentiation conditions compared with that in Ad-shControl (Fig. 3 E). ARL6IP1 is predominantly localized at the ER membrane (Lui et al., 2003; Yamamoto et al., 2014). Therefore, we evaluated changes in intracellular organelles depending on the level of ARL6IP1. Expression levels of VDAC1 (a mitochondrial marker) and calnexin (an ER marker) were dynamically changed in an ARL6IP1 dose-dependent manner. Also, selective organelle clearance through autophagy is critical for regulating cellular organelle homeostasis. Active LC3B (LC3B-II) levels increased or decreased in an ARL6IP1 dose-dependent manner (Fig. 3 F). Neurons consume a lot of energy and use autophagy to break down damaged mitochondria and other cellular components to produce ATP. This process replaces impaired mitochondria with new and improved ones, improving ATP production efficiency. Impaired mitochondrial function is a common feature in various neurodegenerative diseases. Mitochondrial membrane potential (MMP) represents the electrical potential difference between the inner and outer membranes of mitochondria, serving as a crucial indicator of mitochondrial function. High MMP (ΔΨm) is the driving force for mitochondrial ATP production; MMP loss is directly related to cell damage. To investigate changes in MMP in MEFs, MEFs were isolated from both *Arl6ip1*^*−/−*^ (*ARL6IP1* KO) and *Arl6ip1*^*+/+*^ (WT) mice on embryonic days 12 and 13 (E12–13). After carbonyl cyanide m-chlorophenyl hydrazone (CCCP) treatment to induce mitochondrial dysfunction, cells were observed in 5,5′,6,6′-tetrachloro-1,1′,3,3′-tetraethyl benzimidazolyl carbocyanine iodide (JC-1) staining under a fluorescence microscope and quantified by determining the ratio between the red and green fluorescence (Fig. 3 G). The ratios of red/green fluorescence were 1.58 ± 0.46 and 0.7 ± 0.25 in *ARL6IP1* WT and KO MEFs under the untreated condition, and after CCCP treatment, they were confirmed as 0.809 ± 0.05 and 0.623 ± 0.18, respectively. The results show that *ARL6IP1* silencing can cause decay in MMPs. Furthermore, expression of ROS-related genes, which reflect mitochondrial function, was markedly increased in *ARL6IP1* KO than in WT MEFs (Fig. 3 H), thereby sequentially inducing apoptosis (Fig. 3 I). To evaluate oxidative-induced cellular senescence in WT and *ARL6IP* KO MEFs, senescent cells were quantified via senescence-associated β-galactosidase activity at passages 3 and 5. Rapid cellular senescence was observed after passage three in *ARL6IP1* KO, whereas WT MEFs had consistent growth until passage 5 (Fig. 3, J and K).

### ARL6IP1 is essential for autophagosome formation and regulates mitophagy through interaction with BCL2L13

**Figure 4.**
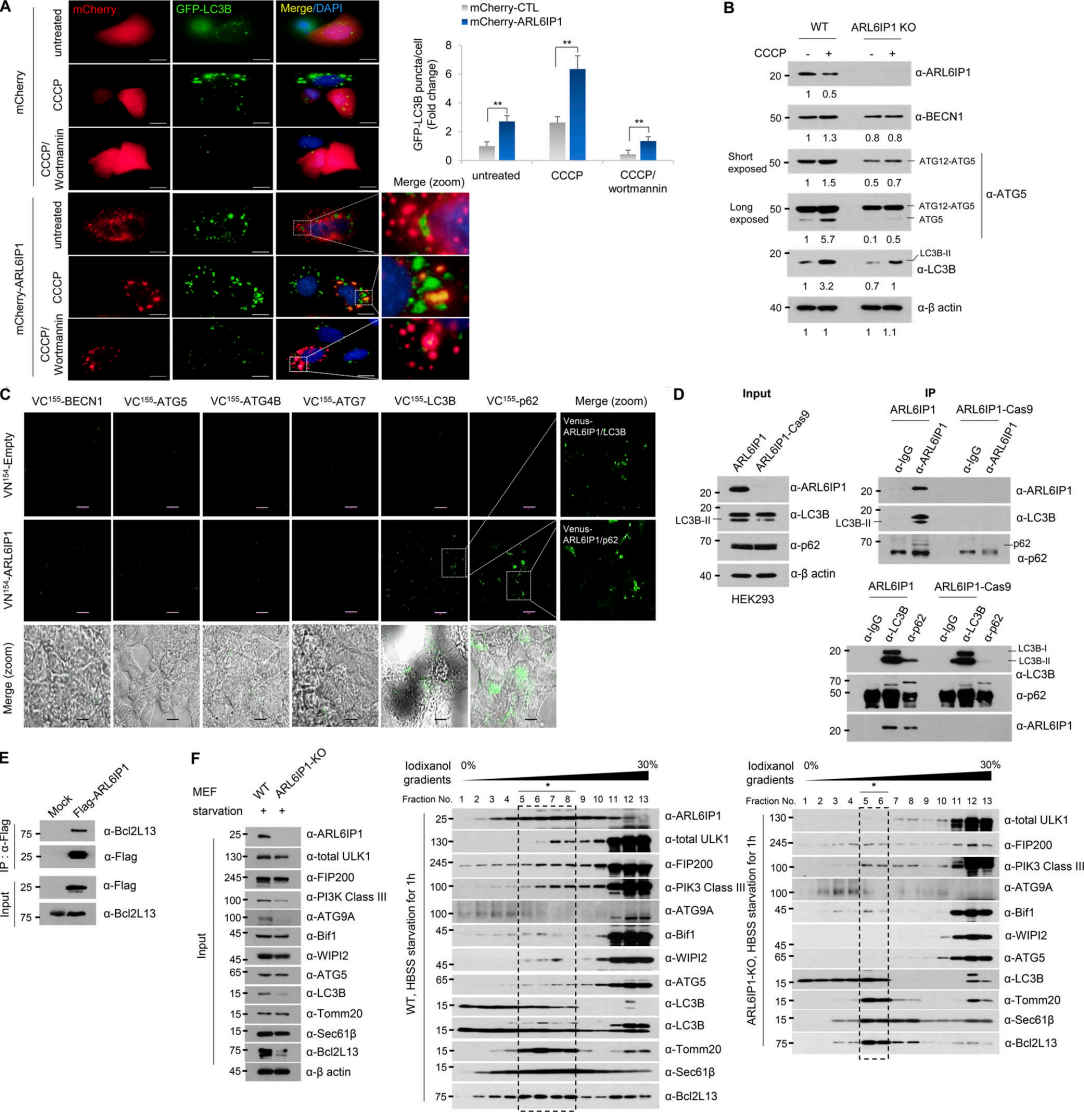
**ARL6IP1 regulates mitophagy through interaction with LC3B and BCL2L13 in autophagosome formation. (A)** Immunofluorescence detection of LC3B-II puncta in HeLa cells transfected with mCherry-empty vectors or mCherry-ARL6IP1 after 20 μM CCCP and/or 10 μM wortmannin treatment for 6 h (left, 600×; scale bar: 20 μm). Quantification of LC3B-II puncta per cell in HeLa cells stably expressing GFP-LC3B (right). Data presented as mean values of triplicates ± SD (≥10 images assessed per group). **P < 0.01. **(B)** Protein levels of autophagosome-related genes were analyzed using western blotting in *ARL6IP1* WT and KO MEFs at three passages after 10 µM CCCP treatment for 6 h. **(C)** Live-cell fluorescence image of HeLa cells cotransfected with indicated vectors (400×; scale bar: 50 μm) observed under a fluorescence microscope and magnified in a bright field (600×; scale bar: 20 μm). **(D)** Interaction of endogenous ARL6IP1, LC3B, and p62 by IP-western blotting in *ARL6IP1* KO HEK293T cells via CRISPR/Cas9-mediated gene editing. **(E)** Interaction of ARL6IP1 and BCL2L13 by IP-western blotting in HEK293T cells. **(F)** *ARL6IP1* KO and WT MEFs were cultured in starvation medium (HBSS) for 1 h. After flotation assay in iodixanol gradients, fractions were detected by western blotting. Asterisks indicate the floatation of omegasome-related proteins from *ARL6IP1* KO and WT MEFs. The arrow indicates the position of the ATG9A protein. Data represent averages of three independent biological replicates. Source data are available for this figure: SourceData F4.

**Figure S3.**
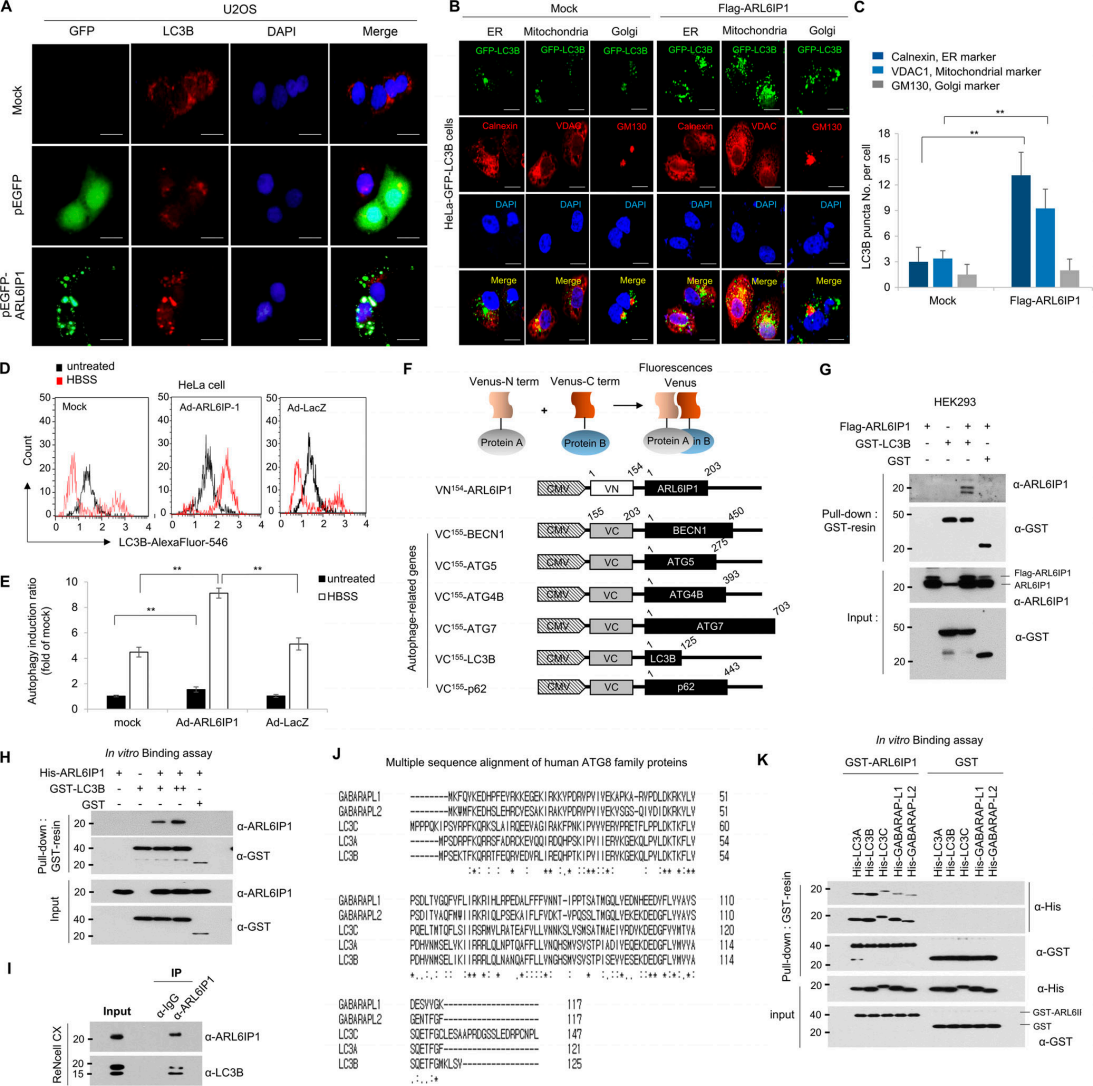
**Activation of autophagy signal by ARL6IP1 via interaction with LC3B. (A)** Immunofluorescence staining of ARL6IP1 and LC3B in U2OS cells transfected with pEGFP-tagged ARL6IP1 or pEGFP-empty vectors (600×; scale bars: 20 μm). **(B)** Immunofluorescence co-localization of LC3B puncta against calnexin, VDAC, and GM130 in HeLa cells expressing GFP-LC3B transfected with FLAG-tagged ARL6IP1 (600×; scale bars: 20 μm). **(C)** Quantification of LC3B puncta per organelle marker positive cell. Data represent mean ± SD of 10 images (**P < 0.01). Antibodies against the calnexin (endoplasmic reticulum), VDAC-1 (mitochondria), and GM130 (Golgi) used as organelle marker proteins. **(D and E)** HeLa cells were transduced with Ad-ARL6IP1 or Ad-LacZ at MOI 100, and incubated for 6 h in an HBSS medium, before using the Muse Autophagy LC3-antibody-based kit with the MUSE cell analyzer to detect autophagy. Cytofluorimetric plots of LC3B-II level quantified on a Muse cell analyzer. Data presented as mean values of triplicates ± SD (**P < 0.01). **(F)** Schematic representation of BiFC constructs involving ARL6IP1 and autophagy-related genes. **(G)** HEK293 cells transiently transfected with FLAG-tagged ARL6IP1 and GST-tagged LC3B or GST empty vectors (5 μg each). Interaction between exogenous ARL6IP1 and LC3B was detected using GST pull-down and western blot analysis. **(H)** In vitro binding assay was performed with purified 0.5 µg His-tagged ARL6IP1 and 0.5 µg GST-tagged LC3B or GST protein from the *E. coli* expression system; analysis was performed via GST pull-down and western blotting. **(I)** Immunoprecipitation experiments of ARL6IP1 and LC3B from ReNcell CX. **(J)** Sequence alignment of human LC3/GABARAP proteins. **(K)** In vitro binding assay between ARL6IP1 and ATG8 family proteins purified from *E. coli* expression system. All experiments were performed by three independent biological replicates. Source data are available for this figure: SourceData FS3.

**Figure S4.**
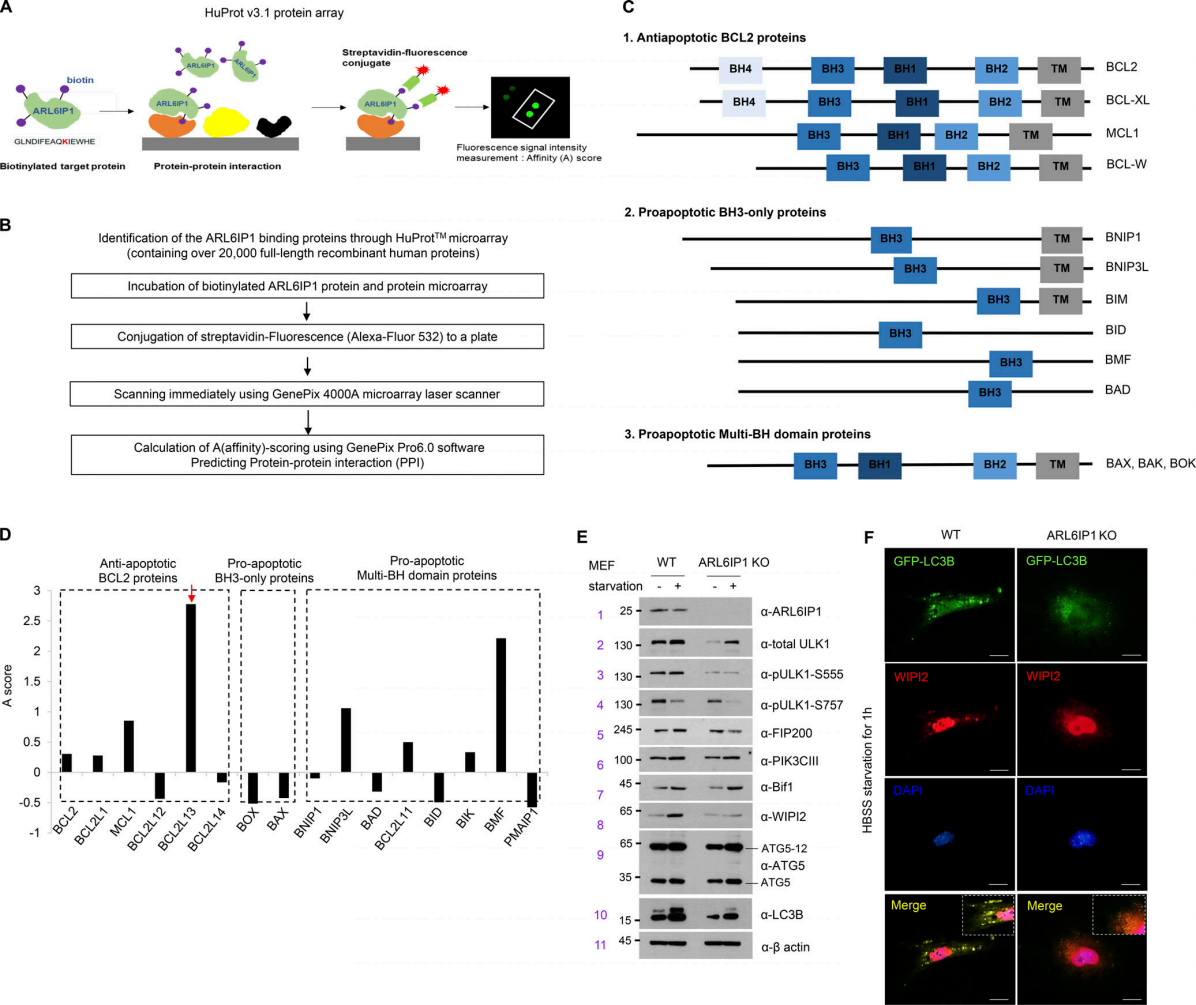
**The identification of the interaction between BCL2L13 and ARL6IP1 in regulating autophagosome formation. (A)** Schematic representation of protein microarray with >21,000 human proteins (HuProt v3.1 protein array). **(B)** Schematic diagram of protein microarray data analysis and filtering method involving ARL6IP1 protein. **(C)** Classification of BCL2 family members based on domain organization predicted to bind with ARL6IP1. BH; BCL2 homology domains, TM; transmembrane domains. **(D)** Quantifying the interaction affinity score between ARL6IP1 and BCL2 family genes using an array. **(E)** The protein level of ARL6IP1 and omegasome-related genes in *ARL6IP1* KO and WT MEFs after HBSS starvation for 1 h. **(F)** Immunofluorescence colocalization of LC3B and WIPI2 from *ARL6IP1* KO and WT MEFs after HBSS starvation. Experiments (E and F) were performed on two independent biological repeats. Source data are available for this figure: SourceData FS4.

To confirm the autophagic flux according to ARL6IP1 expression, autophagic flux was observed in stable green fluorescent protein (GFP)-LC3B HeLa cells transfected with mCherry-ARL6IP1, which showed that the ARL6IP1-transfected group had markedly increased GFP-LC3B puncta compared with that in the control group following autophagy induction by CCCP, while reduced GFP-LC3B puncta following autophagy inhibition by wortmannin (Fig. 4 A). To determine whether an increase in active LC3B according to the ARL6IP1 level induces autophagosome formation in specific organelles, we showed that GFP-LC3B HeLa cells that demonstrated enhanced LC3B-positive puncta and exogenously expressed ARL6IP1 and LC3B puncta were colocalized in the ER and mitochondria after ARL6IP1 overexpression (Fig. S3, A–C). For quantification of active LC3B according to ARL6IP1 expression, the autophagy induction ratio was significantly induced by ARL6IP1 expression (Fig. S3, D and E). A *LacZ*-expressing adenovirus was used as a negative control for the adenoviral vector. ARL6IP1 overexpression induced autophagosome formation by increasing active LC3B levels, indicating that ARL6IP1 is involved in intracellular autophagy signaling. Thus, ARL6IP1 is likely involved in the activation of several sequential steps of autophagy. To clarify the mechanism underlying autophagy regulation by ARL6IP1, we investigated the changes in the expression of autophagy-related gene (ATGs) protein according to the expression of ARL6IP1 in *ARL6IP1* KO MEFs. Levels of protein encoded by ATGs were significantly reduced in *ARL6IP1* KO MEFs compared with those in WT, either in the condition of autophagy induction by CCCP treatment or in the absence of treatment (Fig. 4 B). The bimolecular fluorescence complementation (BiFC) assay was used to directly visualize protein–protein interactions (PPIs) in living cells; ARL6IP1 and ATG-fusion proteins were generated with the N-terminal or C-terminal half of Venus, respectively (Fig. S3 F). BiFC visualized the binding between ARL6IP1 and either LC3B or p62 in HeLa cells (Fig. 4 C). Biochemical interactions between ARL6IP1 and LC3B were confirmed via glutathione S-transferase (GST) pull-down assays in a dose-dependent manner in cell and in vitro (Fig. S3, G–I). LC3B belongs to the LC3/GABARAP protein family, comprising seven family proteins (LC3A [two splice variants], LC3B, LC3C, GABARAP, GABARAPL1, and GABARAPL2; Schaaf et al., 2016). To determine the specific binding between ARL6IP1 and the LC3/GABARAP protein family, recombinant proteins of the GST-ARL6IP1 and His-LC3/GABARAP family were analyzed using pull-down assays; ARL6IP1 was found to bind to all LC3/GABARA*P* family proteins (Fig. S3, J and K). Furthermore, we confirmed that the LC3B II and p62 interacted in the presence of ARL6IP1 and formed a ternary complex with ARL6IP1, while the amount of LC3B-II and p62 complex significantly reduced in the absence of ARL6IP1 (Fig. 4 D). In previous results, we indicated that ARL6IP1 depletion increased the total apoptotic cell population (Fig. 3 E) and induced dysfunctional mitochondria by JC-1 assay (Fig. 3 G). Also, it was confirmed that overexpression of ARL6IP1 induced autophagosome formation in ER and mitochondria (Fig. S3, B and C). To identify mitochondrial partners of ARL6IP1, recombinant His-tagged ARL6IP1 protein purified in *E. coli* was biotinylated and performed predicting PPI between ARL6IP1 and >21,000 human proteins through human proteome microarray (HuProt v4.0; Fig. S4, A and B). As a result, the interaction of anti- and pro-apoptotic (multi BH domain proteins) BCL2 family was confirmed by calculating the affinity (A) score of the proteins that bind to ARL6IP1 (Fig. S4, C and D). We verified the interaction between ARL6IP1 and Bcl-2-like protein 13 (BCL2L13), which are BCL2 family proteins that bind to each other with an A-score of 2 or higher and contain the transmembrane (TM) domain (Fig. 4 E). Recently, it has been reported that Bcl2-L-13 is a mammalian homolog of yeast Atg32 involved in mitophagy (Murakawa et al., 2015). It acts as a mitophagy receptor, maintaining mitochondrial quality and quantity for cellular balance (Murakawa et al., 2019). BCL2L13 recruits the Unc-51-like kinase 1 (ULK1) complex and induces mitophagy through the interaction of LC3B with ULK1 and BCL2L13 (Murakawa et al., 2019). Autophagy induces the activation of the ULK1 and Vps34 complexes. Autophagosome formation initiates with the isolation membrane in omegasomes, which are membrane extensions of the ER where some autophagosomes are formed. To validate phagophore membrane formation according to the ARL6IP1 expression, *ARL6IP1* KO and WT MEFs were subjected to flotation assay after autophagy induction by nutrient deficiency. *ARL6IP1* deficiency reduced membrane flotation of phagophore compared with WT (Fig. 4 F). In *ARL6IP1* KO MEFs, activation of ULK1 complex due to nutrient deprivation was minimized, and WIPI2, an essential factor for autophagosome formation, was no obvious change (Fig. S4, E and F). These results suggest that ARL6IP1 is essential for autophagome formation through phagophore expansion from the ER and regulated mitophagy by interaction with LC3B and BCL2L13.

### ARL6IP1 deficiency reduces physical and functional ER–mitochondria coupling

**Figure 5.**
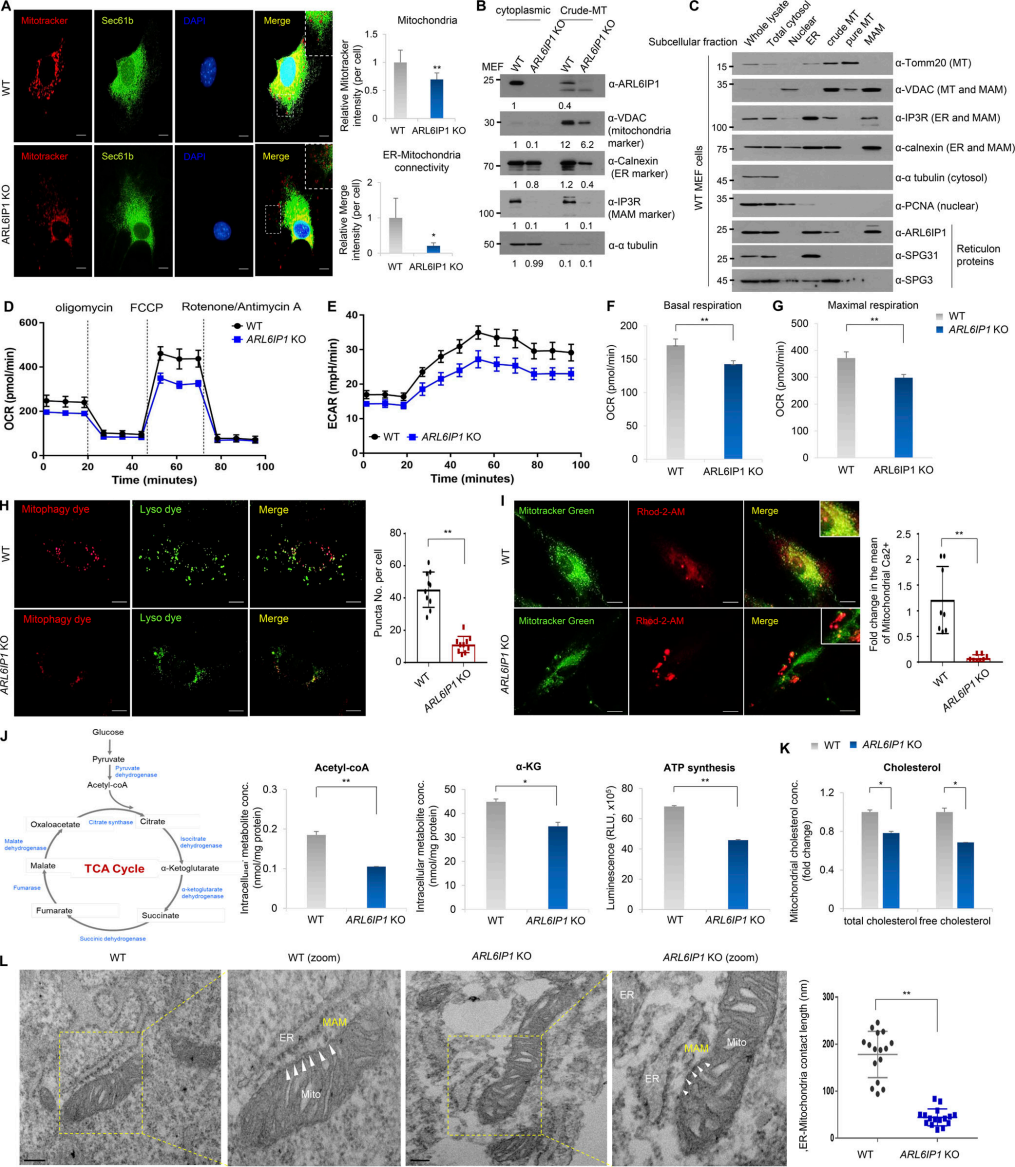
***ARL6IP1* deficiency reduces physical and functional coupling of ER–mitochondria. (A)** Representative image of immunofluorescence staining of MitoTracker Deep Red and Sec61β in *ARL6IP1* KO or WT MEFs at three passages (600×; scale bar: 20 μm). **(B)** Protein levels of organelle markers in cytoplasmic and crude mitochondria (MT) fraction isolated from *ARL6IP1* KO or WT MEFs at three passages. **(C)** Subcellular fractions were isolated via Percoll density gradient ultracentrifugation from WT MEFs at three passages and analyzed using western blotting. **(D and E)** Oxygen consumption rate (OCR) and extracellular acidification rate (ECAR) were measured using Seahorse XF24 Extracellular Flux Analyzer in *ARL6IP1* WT and KO MEFs at two passages (*n* = 5/group). **(F and G)** Quantification of basal and maximal OCR rate using Wave Controller 2.6 (Agilent Seahorse XFe24 Analyzers). Data represent mean ± SD of *n* = 5/group and two independent experiments. **(H)** Representative mitophagy image in CCCP-treated *ARL6IP1* KO or WT MEFs at three passages using Mitophagy detection kit (left, 600×; scale bar: 20 μm). Quantification of autophagosome/lysosome fusion by puncta numbers in *ARL6IP1* KO or WT MEFs (right). Data represent mean values of ≥10 images assessed per experiment ± SD, calculated in GraphPad Prism 7.0. **P < 0.01 **(I)** Colocalization in fluorescent images by Rhod-2 AM and MitoTracker Green in *ARL6IP1* KO or WT MEFs. Rhod-2 AM is used to indicate mitochondrial Ca^2+^ levels (left, 600×; scale bar: 20 μm). Quantification of immunofluorescence staining. Data represent mean ± SD of ≥10 images/group and are calculated in GraphPad Prism 7.0 (right). **(J)** Intracellular acetyl-CoA, α-ketoglutarate, and ATP synthesis levels were measured in *ARL6IP1* WT and KO MEFs at three passages. Data represent mean ± SD of triplicates per group in three independent experiments. **(K)** Mitochondrial total and free cholesterol levels were quantified using cholesterol assay kit in *ARL6IP1* WT and KO MEFs and represent three independent experiments. Data represent mean values of triplicates ± SD. **(L)** Representative TEM images of ER–mitochondria tethering in *ARL6IP1* KO or WT MEFs. ER–mitochondria contact sites were indicated by arrowheads (left, 52,000×; scale bar: 200 nm; ER, endoplasmic reticulum; Mito, mitochondria; MAM, mitochondria-associated ER membranes). Quantification of ER–mitochondria contact length represents mean ± SD of ≥10 images/group and is calculated in GraphPad Prism 7.0 (right). *P < 0.05; **P < 0.01. Data represent averages of three independent biological replicates. Source data are available for this figure: SourceData F5.

In previous reports, it has been shown that the phagophore membrane forms at ER–mitochondrial junctions, known as MAM (mitochondria-associated ER membranes), in mammalian cells (Hamasaki et al., 2013; Zachari and Ganley, 2017). To verify that ARL6IP1 is a novel member of the MAM, first, ER–mitochondrial connectivity according to ARL6IP1 expression was evaluated; the connection between the ER (Sec61β) and mitochondria (Mitotracker Deep Red) was significantly reduced in *ARL6IP1* KO MEFs compared with that in WT (Fig. 5 A). Total cytosolic and crude mitochondrial lysates were isolated using density gradient centrifugation from *ARL6IP1* KO or WT MEFs, and protein levels of the ER and mitochondrial marker genes were lower in *ARL6IP1* KO MEFs than those in WT (Fig. 5 B). Next, to determine whether ARL6IP1 existed in the MAM fraction, subcellular fractions were isolated via density gradient centrifugation from WT MEFs and analyzed using western blotting with subcellular organelle marker antibodies. The MAM-associated proteins VDAC, IP3R, and calnexin were used as positive controls. ARL6IP1 was detected in ER, crude mitochondria, and MAM fractions, and was confirmed as a novel MAM-associated protein (Fig. 5 C). SPG31 and SPG3, which are also RTN proteins similar to ARL6IP1, were not detected in the MAM fraction. MAMs play a principal role in the regulation of cell survival and death, autophagy, calcium signaling, intracellular lipid exchange, and cellular metabolic homeostasis (Perrone et al., 2020). Mitochondrial dysfunction was assessed by measuring OCR and ECAR. OCR and ECAR were lower in *ARL6IP1* KO MEFs than in WT MEFs (Fig. 5, D and E). The basal and maximal OCR rates were monitored and were significantly decreased in *ARL6IP1* KO MEFs than those in WT (Fig. 5, F and G). Mitophagy, involved in maintaining healthy mitochondria, was significantly reduced in *ARL6IP* KO MEFs compared with that in WT (Fig. 5 H); therefore, ARL6IP1 may be required for mitochondrial quality control by regulating mitophagy. MAMs in close contact between the ER and mitochondria have been investigated as a zone for the selective transfer of physiological and pathological Ca^2+^ signals. To verify Ca^2+^ uptake into the mitochondrial matrix according to ARL6IP1 signaling, *ARL6IP1* KO and WT MEFs were cultured with a fluorescent Ca^2+^ indicator, Rhod-2 AM, and observed under a fluorescence microscope. Mitochondrial Ca^2+^ was significantly decreased in *ARL6IP1* KO MEFs compared with that in WT (Fig. 5 I). Mitochondrial Ca^2+^ improves ATP production by regulating tricarboxylic acid (TCA) cycle dehydrogenase (i.e., pyruvate, isocitrate, and oxoglutarate dehydrogenase) activity (Glancy and Balaban, 2012). We assessed the alteration of the TCA cycle and found that concentrations of intracellular acetyl-CoA and α-ketoglutarate as TCA cycle products were significantly inhibited in *ARL6IP1* KO MEFs compared with that in WT. Likewise, ATP production was reduced in *ARL6IP1* KO MEFs (Fig. 5 J). Mitochondria require cholesterol for biogenesis and membrane structure, and mitochondrial cholesterol trafficking occurs in MAM. We isolated mitochondrial fractions from *ARL6IP1* KO and WT MEFs, and mitochondrial total and free cholesterol concentrations were measured. *ARL6IP1* KO MEFs had lower mitochondrial cholesterol levels than WT MEFs (Fig. 5 K). Lower cholesterol levels in mitochondrial membranes by *ARL6IP1* KO correlated with previous results of loss of MMP and increased apoptosis. Finally, to further corroborate these data, transmission electron microscopy (TEM) was performed on *ARL6IP1* KO and WT MEFs. *ARL6IP1* silencing significantly reduced the membrane contact site between ER and mitochondria, suggesting that ARL6IP1 plays a structural role in ER–mitochondria connectivity in MAM (Fig. 5 L).

### *ARL6IP1* gene transfer restores behavioral abnormalities and histopathologic changes in vivo

**Figure S5.**
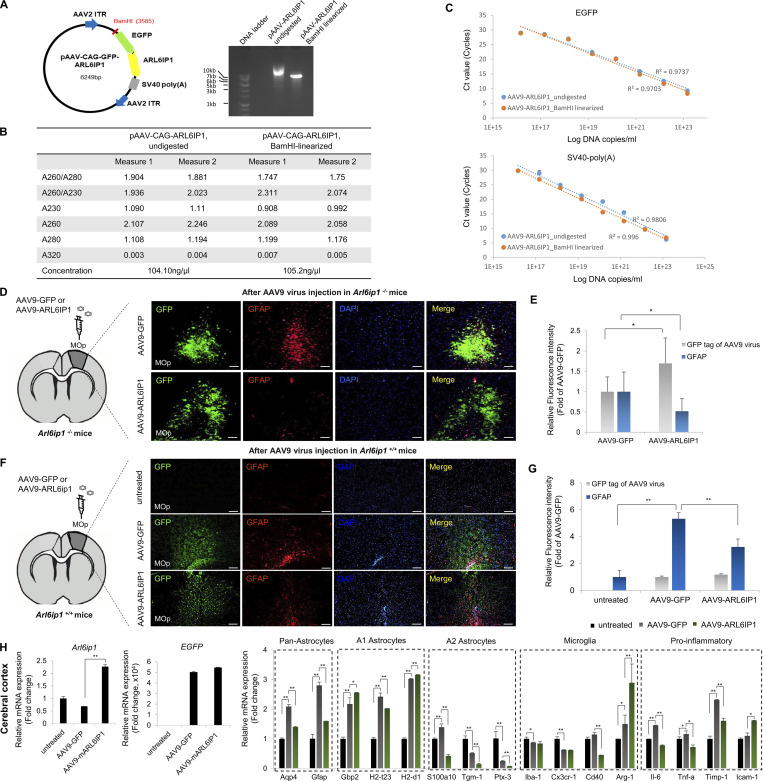
**The validation of AAV9 gene delivery in the primary motor cortex region of *Arl6ip1***^***−/−***^ **and *Arl6ip1***^***+/+***^ **mice. (A)** Schematic representation of the AAV9-CAG-GFP-ARL6IP1 plasmid and the BamHI restriction sites. Representative image of gel electrophoresis of an undigested and digested plasmid. **(B)** After BamHI digestion, the purity and concentration of AAV9-CAG-GFP-ARL6IP1 plasmid were measured using a nanodrop spectrophotometer. **(C)** AAV9-CAG-GFP-ARL6IP1 plasmid was serially diluted and analyzed by qPCR using EGFP and SV40 primers. A qPCR standard curve was created by plotting Ct values against the corresponding Log DNA copies/ml. **(D and E)** Representative immunofluorescence images and quantification of GFP, and GFAP in the primary motor cortex region of *Arl6ip1*^*−/−*^ mice after AAV9 gene delivery (200×; scale bars: 100 μm). **(F and G)** Representative immunofluorescence images and quantification of GFP, and GFAP in the primary motor cortex area of *Arl6ip1*^*+/+*^ mice after AAV9 gene delivery (200×; scale bars: 100 μm). Data (E and G) represent mean ± SD of *N* = 5 mice/group and the two-tailed unpaired *t* test. *P *<* 0.05, **P < 0.01. **(H)** After AAV9 gene delivery, *Arl6ip1*, and EGFP mRNA levels were confirmed for gene transfer efficiency in the cerebral cortex of *Arl6ip1*^*+/+*^ mice. mRNA levels of glial or pro-inflammatory marker genes after AAV9 gene delivery. Data are represented with the average of triple technical repeats, the mean and error bars represent the SEM, and the two-tailed unpaired *t* test. *P < 0.01, **P < 0.001 (*n* = 3 mice/group). All experiments were performed in three independent biological replicates. Source data are available for this figure: SourceData FS5.

**Figure 6.**
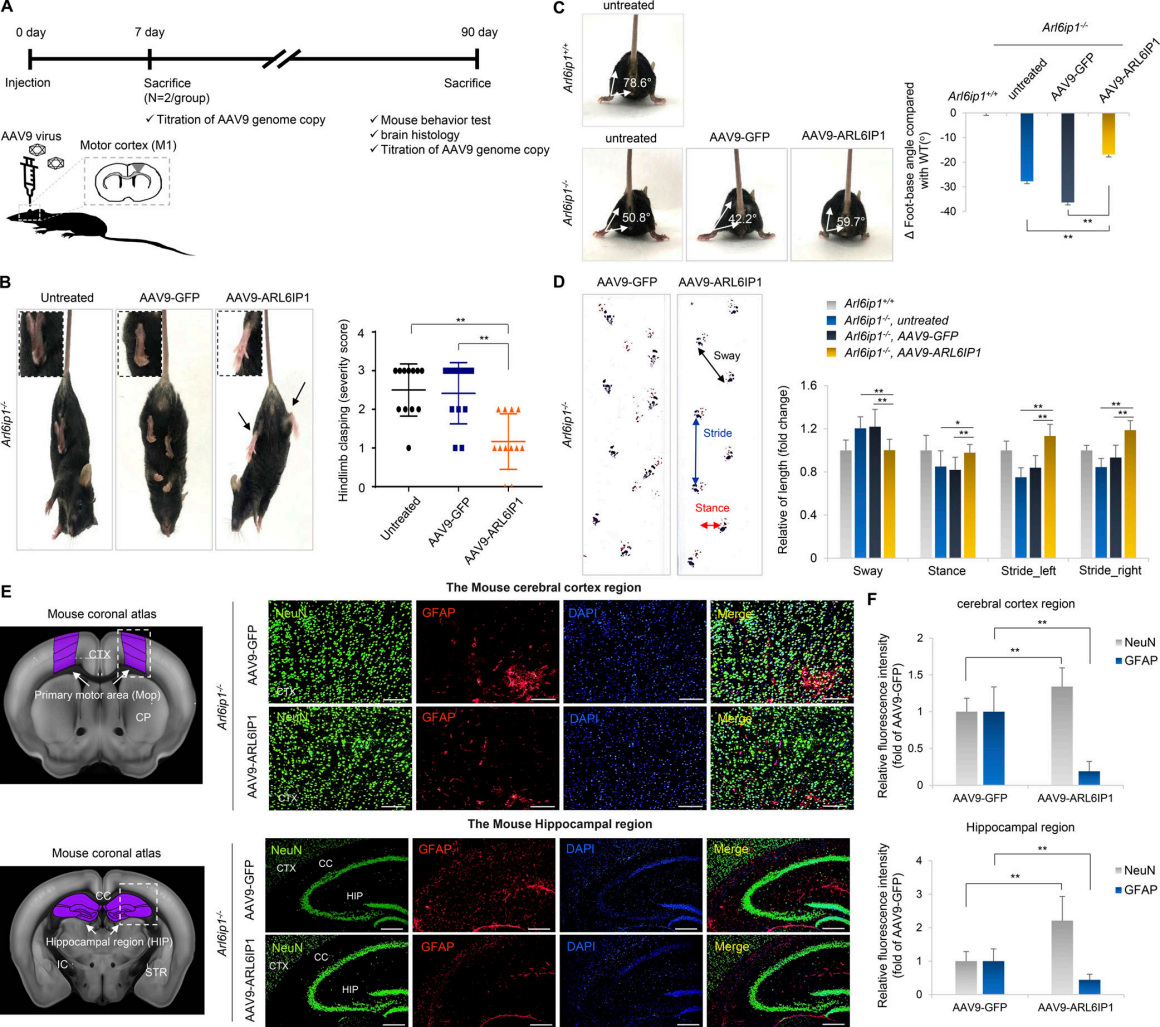
**Restoration of behavioral abnormalities and histopathology by *ARL6IP1* gene transfer. (A)** Experimental design for AAV9 gene therapy. Each AAV9 vector was delivered to *Arl6ip1*^*−/−*^ mice with 1 μl AAV9 virus (2 × 10^13^ Vg/ml) per primary motor cortex (M1), with two injection sites/mouse (*n* = 20 mice/group). **(B)** Representative image of improvement of abnormal limb reflex in *Arl6ip1*^*−/−*^ mice after AAV9 gene delivery (left). Quantification of hindlimb clasping score in *Arl6ip1*^*−/−*^ mice after AAV9 gene delivery. Data represent mean ± SD of *n* = 18/group and are calculated in GraphPad Prism 7.0 (right). **(C)** Representative image (left) and measurement of foot-base angle (right) of *Arl6ip1*^*−/−*^ mice after AAV9 gene delivery. *Arl6ip1*^*+/+*^ mice of the same age were used as positive controls for behavioral analysis. Data represent mean values of *n* = 18/group ± SD. **(D)** Representative image showing improvement in disturbed footprint pattern in *Arl6ip1*^*−/−*^ mice after AAV9 gene delivery (left). Stride, stance, and sway length were calculated from footprint patterns of *Arl6ip1*^*−/−*^ mice (right). Data represent mean ± SD of *n* = 8 mice/group. **(E)** Mouse coronal section image from Allen Adult Mouse Brain Atlas showing primary motor cortex and hippocampal area (left, marked in purple). Immunofluorescence staining of NeuN and GFAP in the cerebral cortex (top) and hippocampal regions (down) of *Arl6ip1*^*−/−*^ mice after AAV9 gene delivery (200×; scale bar: 100 μm). **(F)** Quantification of immunofluorescence staining. Data represent mean ± SD of *n* = 5 mice/group and are analyzed using Student’s *t* test. **P < 0.01. Data represent averages of three independent biological replicates.

To evaluate *ARL6IP1* gene therapy, we constructed an AAV9 serotype with a chicken β-actin promoter that drives expression in *Arl6ip1*^*+/+*^ mice and estimated its expression in *Arl6ip1*^*−/−*^ mice, an HSP model. *Arl6ip1*^*−/−*^ mice start showing initial mild phenotypic manifestations at 3 mo progressively exacerbating at 5 and 7 mo, culminating in pronounced symptoms by 9 mo. To evaluate the therapeutic effects, we performed a stereotaxic injection of AAV9-ARL6IP1 into *Arl6ip1*^*−/−*^ mice at 2 mo of age (8 wk old), even before they displayed any symptoms. After injection, two mice per group were euthanized; the genome copy number of the AAV9 virus was calculated in the mouse tissues at 7 and 90 d (Table S10 and Fig. S5, A–C), and then we monitored the differences in symptoms between the groups, and the analysis was conducted 3 mo (5 mo old) after the gene therapy injection when the differences in motor function began to manifest (Fig. 6 A). AAV9-ARL6IP1 gene transfer reduced hindlimb clasping score in *Arl6ip1*^*−/−*^ mice, while untreated mice exhibited hindlimb spasticity (Fig. 6 B). Furthermore, foot-base angles of *Arl6ip1*^*−/−*^ mice were ∼50.8° and ∼42.2° in untreated or AAV9-GFP treated mice, respectively; the foot-base angle was restored to ∼59.7° in AAV9-ARL6IP1 treated mice (Fig. 6 C). At 90 d after injection, gait analysis using the footprint test was performed in *Arl6ip1*^*−/−*^ mice; on *ARL6IP1* gene transfer, stance length increased, stride length improved, and sway length decreased (Fig. 6 D). AAV9-GFP or ARL6IP1 injection into the primary motor cortex was confirmed via GFP expression and the reduction of GFAP-positive cells was observed in the same region after AAV9-ARL6IP1 injection compared with the AAV9-GFP group (Fig. S5, D and E). An increase in NeuN-positive cells and a decrease in GFAP-positive cells were observed in the cerebral and hippocampus regions of *Arl6ip1*^*−/−*^ mice injected with AAV9-ARL6IP1 (Fig. 6, E and F). Additionally, we investigated the overexpression effects of AAV9-ARL6IP1 gene delivery on *Arl6ip1*^*+/+*^ mice. Our results showed that the injection of the AAV9 virus alone led to the activation of reactive astrocytes, both in AAV9-GFP- and AAV9-ARL6IP1-treated mice. However, in the case of AAV9-ARL6IP1, there was a reduction in the proinflammatory response due to the modulation of M1 and M2 microglia polarization (Fig. S5, F–H).

### *ARL6IP1* gene transfer restores CNS remyelination ability and reverses histopathological changes

**Figure 7.**
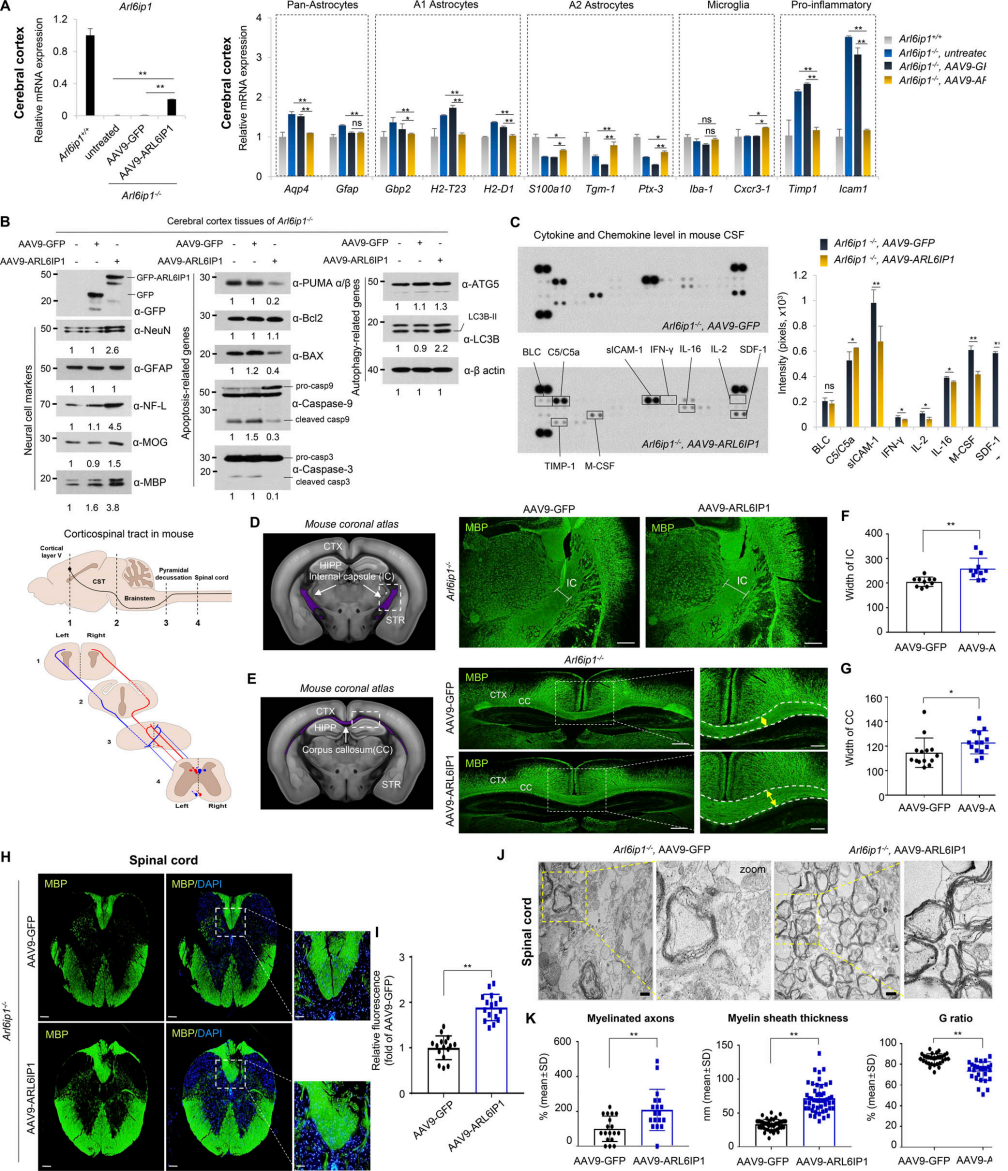
**Restoration of CNS remyelination capacity by *ARL6IP1* gene transfer. (A)** After AAV9 gene delivery, *Arl6ip1* mRNA levels were confirmed using RT-qPCR for gene transfer efficiency in the cerebral cortex of *Arl6ip1*^*−/−*^ mice. mRNA levels of glial or pro-inflammatory marker genes after AAV9 gene delivery compared with that in *Arl6ip1*^*+/+*^. Data represent mean ± SEM of *n* = 4 mice/group. **(B)** Change in protein levels of neural and glial markers (left) and of apoptosis- (middle) or autophagy-related genes (right) from pooled lysates in mouse cortex after AAV9 gene delivery (*n* = 4 mice/group). **(C)** Changes in mouse cytokine and chemokine levels in CSF samples after AAV9 gene delivery. A bar graph shows the quantification of cytokine array analysis. Data represent mean ± SD of *n* = 8 mice/group. **(D and E)** Mouse coronal section image from Allen Adult Mouse Brain Atlas showing internal capsule and corpus callosum area (left, marked in purple). Representative image of MBP staining of the IC and CC from *Arl6ip1*^*−/−*^ after *ARL6IP1* gene delivery (40×; scale bar: 500 μm). CTX, cerebral cortex; HIPP, hippocampus, STR, striatum; IC, internal capsule; CC, corpus callosum. **(F and G)** Quantification of myelin thickness (MBP-positive fibers) in IC and CC region. Data represent mean ± SD of 10 images/group and are calculated in GraphPad Prism 7.0 (*n* = 5 mice/group; *P < 0.05; **P < 0.01). **(H)** Representative image of MBP fluorescence staining in the spinal cord from *Arl6ip1*^*−/−*^ after AAV9 gene delivery, and highlighted in corticospinal tract of mouse spinal cord (40×; scale bar: 500 μm, 400×, scale bar: 50 μm). **(I)** Quantification of myelin (MBP staining) of white matter in the spinal cord. Data represent mean ± SD of 10 images/group, calculated in GraphPad Prism 7.0 (*n* = 5 mice/group; **P < 0.01). **(J)** Representative TEM images of spinal cord white matter in *Arl6ip1*^*−/−*^ after AAV9 gene delivery (*n* = 3 mice/group, 15,000×; scale bar: 800 nm). **(K)** Quantitative data of the percentage of myelinated axons, G ratio, and myelin sheath thickness. Data represent mean ± SD of 10 images/group and are calculated in GraphPad Prism 7.0. *P < 0.05; **P < 0.01. Data represent averages of three independent biological replicates with two technical replicates for each. Source data are available for this figure: SourceData F7.

Following *ARL6IP1* gene transfer, mRNA levels of *Arl6ip1* (Fig. 7 A, left) and various glial cells were confirmed in the cerebral cortex surrounding the injection site. The cortex from the AAV9-ARL6IP1-injected group showed a reduction in A1 reactive astrocytes and proinflammatory mediators that induce neuronal cell death and showed an increase in A2 reactive astrocytes and microglia that induce neuronal survival by releasing neurotrophic factors and anti-inflammatory cytokines (Fig. 7 A, right). Changes in protein levels in neurons and glial cells were investigated in the cortex of *Arl6ip1*^*−/−*^ mice following gene transfer; the expression of NeuN, NF-L, and MOG proteins was increased, whereas that of the active astrocyte marker GFAP protein was decreased following *ARL6IP1* gene transfer. Furthermore, autophagy-related protein levels were also upregulated, whereas apoptosis-related protein levels were downregulated in the mouse cortex tissues of *Arl6ip1*^*−/−*^ after gene delivery (Fig. 7 B). Changes in microglia, astrocytes, and neuroinflammation in accordance with ARL6IP1 expression were analyzed in the CSF through proinflammatory cytokines and chemokines. On *ARL6IP1* gene transfer, increased BLC, C5/C5α, M-CSF, sICAM-1, TIMP-1, and TNF-α levels in *Arl6ip1*^*−/−*^ mice were reduced, and CSF levels of IFN-γ, IL-2, IL-16, and SDF-1 were also decreased (Fig. 7 C). To confirm the recovery of nerve fibers to the corticospinal tract, after AAV9-ARL6IP1 gene transfer, MBP-positive staining, as a mature oligodendrocyte marker, was used to show that myelinated axons were increased in the IC region (Fig. 7, D and F); in addition, TCC, a complex AR-HSP representative phenotype, was restored in *Arl6ip1*^*−/−*^ mice after AAV9-ARL6IP1 gene transfer (Fig. 7, E and G). The level of MBP-positive fiber was increased in the corticospinal tract of the spinal cord of *Arl6ip1*^*−/−*^ mice after *ARL6IP1* gene transfer (Fig. 7, H and I). Electron microscopy analysis confirmed that the number of myelinated axons and myelin sheath thickness were increased in the spinal cord of *Arl6ip1*^*−/−*^ mice following *ARL6IP1* gene transfer (Fig. 7, J and K). These results suggest that *ARL6IP1* gene transfer into the brain of *Arl6ip1*^*−/−*^ mice helps to mitigate HSP disease progression, such as axonal degeneration.

Reverse corelation between SPAST and FBXL17 protein expression was confirmed in mouse embryonic tissues at the neural development stage and ReN cells with induced neuronal differentiation.

## Discussion

In this study, *ARL6IP1* has been identified as a member of the MAM gene family involved in modulating mitochondrial function to maintain the axonal generation of neuronal cells. It has also been implicated in HSP, a typical neurodegenerative disease caused by axonopathy. Indeed, a frameshift mutation (c.576_579delAAAC, p.K193Ffs36X) has been reported in two separate cases of HSP (Novarino et al., 2014; Nizon et al., 2018). In both cases, these patients exhibited congenital insensitivity to pain, acromutilation, and spastic paraplegia, indicating the highly pathogenic nature of this mutation.

To clarify the physiological significance of *ARL6IP1* in vivo, we attempted to generate *Arl6ip1* conditional KO mice using Arl6ip1-Tm1a. Unexpectedly, only Loxp-Arl6ip1-Lox*P* cassette-possessing homozygote mice (Tm1a) showed HSP phenotypes clearly. Although Nestin-Cre-crossed KO mice (Tm1b) showed a more severe pathogenic phenotype earlier than the cassette KO mice, this Tm1b could not be expanded in sufficient numbers for experimentation; therefore, all experiments were conducted using Tm1a mice (named *Arl6ip1*^*−/−*^ mice). We confirmed that the expression of the neighboring genes of *Arl6ip1* on chromosome 7 had no effect in *Arl6ip1*^*−/−*^ mice (data not shown). *Arl6ip1*^*−/−*^ mice showed gait abnormalities, irregular stride lengths, lower leg angles, and pathophysiology of the brain, which are typical HSP phenotypes (Fig. 1). In histopathological identification, evidence of demyelination and neuroinflammation was observed in *Arl6ip1*^*−/−*^ mice (Fig. 2). Microglia-mediated neuroinflammation is a common feature of various neurodegenerative diseases, including Alzheimer’s disease, Parkinson’s disease, amyotrophic lateral sclerosis, and multiple sclerosis. Although the physiological and pathophysiological roles of glial cells in disease are not fully understood, astrocytes and microglia play vital roles in brain functions, such as the regulation of oligodendrocyte homeostasis, during demyelination and remyelination (Domingues et al., 2016). In addition, our mouse model revealed some manneristic behaviors, such as jumping (data not shown). Compared with other SPG mouse models (SPG11 and SPG15), the *Arl6ip1*^*−/−*^ mouse model showed a similar pathogenic phenotype, such as CC thinning (TCC), which is the most common cause of AR-HSP and extensive white matter abnormalities (França et al., 2012; Riverol et al., 2009; Khundadze et al., 2013; Beetz et al., 2013).

The pathophysiological role of ARL6IP1 in HSP was validated using an *Arl6ip1*^*−/−*^ mouse model. The *Arl6ip1*^*−/−*^ mouse model used successfully proved the function of ARL6IP1. Typical spastic limb and neurodegenerative phenotypes were observed in the *Arl6ip1*^*−/−*^ mice; hence, we examined the critical role of ARL6IP1 in maintaining neuronal homeostasis. Furthermore, the therapeutic effect of gene delivery of *ARL6IP1* was assessed to develop gene therapy for HSP.

Four genes encoding reticulon proteins (RTNs), namely *RTN1*, *RTN2*, *RTN3*, and *RTN4* (also known as NOGO), have been discovered in mammals. RTNs possess a conserved C-terminal RTN homology domain characterized by two unusually long hydrophobic regions interrupted by a hydrophilic loop (Chiurchiù et al., 2014). The hydrophobic parts of the RTN domain inserted into lipid bilayers have an unusual hairpin topology between the inner and outer shear controlling the shape of the ER membrane (Pradhan and Das, 2021). These proteins are abundant in the tubular ER and play a critical role in interorganelle communication between the ER and mitochondria (Hübner and Kurth, 2014; Voeltz et al., 2006). Some protein-encoding HSP genes, including *ARL6IP1*, *REEP1*, *ALT1*, and *SPAST*, have been identified as RTNs localized in the ER membrane (Hübner and Kurth, 2014; Voeltz et al., 2006); these play a critical role in shaping the ER, connecting intracellular membrane-bound organelles, and are intrinsic in neurodegenerative disease pathogenesis (Chiurchiù et al., 2014; Pradhan and Das, 2021). ARL6IP1 is an anti-apoptotic regulator of the ER membrane (Lui et al., 2003); its depletion induced neuronal cell death and reduced neuronal axonal outgrowth and extension by inhibiting autophagy signaling (Fig. 3). It was previously identified that ARL6IP1 was localized in the ER membrane; however, it was newly verified to modulate mitophagy via direct interaction with LC3B and BCL2L13 (Fig. 4). Interestingly, cell death induced by ARL6IP1 depletion was immediate and strong via mitochondrial apoptosis and reduced autophagic flux. These results led us to hypothesize that ARL6IP1 is a MAM protein.

MAMs, the contact site between the ER and mitochondria, have critical functions in mitochondrial quality control and dynamics and are associated with neurodegenerative disease and ER networking, while mitochondria are implicated in cellular pathophysiology, especially in long axonal extension of neurons (Paillusson et al., 2016; Wilson and Metzakopian, 2021). MAMs play a critical role in interorganelle interactions and the long projection of motor neurons to the spinal cord. Long extension of axons requires extensive ATP as a mitochondrial energy source, where MAMs recruit mitochondria from the center to the distant axon (Pinton, 2018; Liu and Yang, 2022). MAMs control various critical cellular functions, including lipid exchange, calcium homeostasis, and autophagy, and contribute to the regulation of inflammation in neuronal diseases. In the cellular fraction analysis, ARL6IP1 was found in a MAM fraction, where it regulated various important cellular functions of MAM (Fig. 5). RTN-1C, a member of the RTN family, is a MAM influencing autophagosome formation (D’ Eletto et al., 2019). ARL6IP1 is a modulator for ER and mitochondrial organization in the HSP *Drosophila* model (Fowler and O’Sullivan, 2016). However, this is the first report that ARL6IP1 is a member of MAM in mammals and is associated with regulating autophagy. To this notion, we investigated whether ARL6IP1 is involved in the formation of omegasomes, which are specific structures within the ER involved in mitophagy. Omegasomes have distinct signaling pathways from general autophagy, and it is known that proteins involved in omegasome formation form complexes. We found that ARL6IP1 coexists with the omegasome complex in cells. Based on this finding, we hypothesized that ARL6IP1 functions not only in the MAM but also in omegasome formation, thereby regulating mitophagy.

Lastly, the therapeutic effects of *ARL6IP1* gene delivery were demonstrated in an HSP model. AAV9 was selected for delivery of *ARL6IP1* into the primary motor cortex due to its high transduction efficiency to neurons. At 3 mo after injection, *Arl6ip1*^*−/−*^ mice showed reduced pathogenic phenotypes, including gait abnormality, irregular stride length, and low leg angle (Fig. 6), and restored CNS remyelination ability following reduced neuroinflammation (Fig. 7). In WT mice, AAV9 virus injection induces an increase in reactive astrocytes, which acts as a defensive response to brain injury, inhibiting inflammatory reactions in brain tissue and promoting protection and tissue recovery. On the other hand, AAV9-ARL6IP1 regulates the risk of excessive proinflammation through microglia polarization. These results suggest that *ARL6IP1* overexpression could be a therapeutic option for HSP in cases of loss of ARL6IP1 function. Furthermore, ARL6IP1 gene therapy has the potential to rescue HSP caused by impaired ER and mitochondrial networking. Supplying ARL6IP1 may help maintain normal ER and mitochondrial function, thereby promoting the health of neuronal cells.

## Materials and methods

### Resource table

Reagents and resources are listed in Tables S2 and S3.

### Animals

Mice were housed in a specific pathogen–free animal laboratory (humidity, 60–65%; temperature, 22°C) under a 12/12-h light/dark cycle. All animal housing and experiments were conducted following the Institutional Animal Care and Use Committee Guidelines (KRIBB-AEC-18090) of the Korea Research Institute of Bioscience and Biotechnology (KRIBB). WT mice were used as the control group, and to minimize genetic variation, mice of the same age from the same inbred strain were utilized.

### Generation of *Arl6ip1*^−/−^ mice

To generate *Arl6ip1*^−/−^ mice, heterozygote *Arl6ip1*^*t*m1a(EUCOMM)^ mice were purchased from European Conditional Knockout Mouse Mutagenesis (EUCOMM) and maintained in a pathogen-free housing in the animal facility. Offspring of C57BL/6N inbred females were analyzed using PCR with genomic DNA from mouse toes. PCR conditions were as follows: 5 min at 95°C, followed by 35 cycles at 95°C for 30 s, 60°C for 30 s, and 72°C for 30 s, and a final 5-min incubation at 72°C. The *Arl6ip1* WT allele was detected with the WT primer, resulting in the amplification of a 350-bp fragment, whereas the mutant allele was targeted by the MT primer and amplified to a 120-bp fragment. Based on these results, homologous *Arl6ip1*^−/−^ mice were selected, maintained, and utilized for behavioral and histopathological analyses. The primer sequences are provided in Table S4.

### Plasmid constructions

For the *ARL6IP1* (NM_198737.2) KO model, rat shARL6IP1 was produced by subcloning nt 142–160 (5′-GAT​GCT​GAT​GGC​TGA​CAA​A-3′) into pSuper-GFP/neo (Oligoengine). *ARL6IP1* expression constructs were generated using reverse transcription (RT)-PCR from ReNcell CX cells using specific primers and incorporated into various plasmid vectors as below. Mutated constructs were generated using plasmids containing the *ARL6IP1* WT gene using primers with the desired mutation using PCR-based methods. The pAAV9-CAG-GFP plasmid was purchased from Addgene (Plasmid #37825). All the plasmid constructs were confirmed via sequencing; detailed information is provided in Table S5.

### Cell culture

HEK293, HeLa, U2OS cell lines (KCTC), and GFP-LC3B HeLa (generated GFP-LC3B-expressing cell line) were maintained in Dulbecco’s modified Eagle’s medium (DMEM; Welgene) with 10% fetal bovine serum (FBS; Thermo Fisher Scientific) and penicillin–streptomycin (P/S, #15140122; Thermo Fisher Scientific) in a humidified incubator with 5% CO_2_ at 37°C. ReNcell CX cells, derived from the cortical region of human fetal brain tissue (#SCC007; Merck Millipore) as an immortalized human progenitor cell line, were maintained in ReNcell NSC maintenance medium (SCM005; Merck Millipore) supplemented with 20 ng/ml epidermal growth factor (AF-100-15; PeproTech), 20 ng/ml basic fibroblast growth factor (100-18B; PeproTech), and P/S. For neuronal differentiation of ReNcell CX cells, the ReNcell NSC maintenance medium was used for culturing after removing growth factors. To generate an *ARL6IP1*-Cas9 stable cell line, the ARMER CRISPR/Cas9 (sc-409837) and ARMER homology-induced repair (HDR, sc-409837-HDR) plasmids were purchased from Santa Cruz Biotechnology. HEK293 cells (3 × 10^5^ cells/well in a six-well plate) were cotransfected with 2 μg ARMER CRISPR/Cas9 and 2 μg ARMER-HDR plasmids using the Transporter 5 transfection reagent (Polysciences, Inc.). After 24 h, the HEK293 cells expressing GFP and red fluorescent protein were selected using 5 μg/ml puromycin dihydrochloride treatment (A1113803; Thermo Fisher Scientific) for 2 wk and confirmed using RT-qPCR and western blot analysis. The cells used in this experiment were tested for mycoplasma contamination prior to use using the CycleavePCR Mycoplasma Detection Kit (#CY232; TAKARA Bio).

### Primary cultures of mouse embryonic fibroblasts (MEFs)

For MEF culture, E12.5–13.5 *Arl6ip1*^−/−^ and *Arl6ip1*^+/+^ mice were euthanized by carbon dioxide (CO_2_) inhalation and the embryos were separated from their placentas and washed with Dulbecco’s phosphate-buffered saline (DPBS, SH30028; Cytiva Hyclone) three times to remove the residual blood. The embryonic bodies were minced, washed with sterile PBS, treated with 0.25% trypsin-EDTA (#15400054; Thermo Fisher Scientific) at 37°C for 5 min to dissociate the cells, and passed through a 40-μm cell strainer (#352340; Corning Inc.) to eliminate clumps and debris. Then, the passaged cells were centrifuged and maintained in DMEM with 10% FBS and P/S in a humidified incubator with 5% CO_2_ at 37°C; the medium was changed routinely every 2–3 d; cells used in this study were between passages 2 and 4.

### Transient cell transfection

HEK293 cells were transfected with the indicated plasmid constructs using the Transporter 5 transfection reagent and U2OS, or HeLa cells were transfected using Lipofectamine 3000 Reagent (L3000015; Thermo Fisher Scientific) following the manufacturer’s instructions. ReNcell CX cells were harvested, resuspended in BTX electroporation buffer (#45-0805; BTX), and transfected using the ECM830 electroporation system (BTX). Electroporation was performed using one pulse at a 70 V discharge for 30 ms.

### RT-qPCR

Total RNA was isolated using an RNeasy Mini Kit (#74104; Qiagen) from cells and tissues. cDNA was synthesized from 1 μg of total RNA using a Verso cDNA Synthesis Kit (AB1453A; Thermo Fisher Scientific) according to the manufacturer’s instructions. For RT-qPCR, the reaction mixture was prepared in a total volume of 20 µl containing 2 µl of template, 0.8 µl of each primer (final concentration of 400 nM), and 10 µl of 2× Brilliant III SYBR Ultra-Fast qPCR Master Mix (Agilent). PCR was performed according to the manufacturer’s instructions (AriaMx Real-Time PCR System; Agilent) as follows: 95°C for 3 min, followed by 40 cycles of 5 s at 95°C and 10 s at 60°C. Melting curve analysis was performed under the following conditions: 30 s at 95°C, 30 s at 65°C, and 30 s at 95°C. After normalization against *GAPDH* mRNA levels, relative quantification of gene expression was performed using the 2^−ΔΔCT^ method (Livak et al., 2013). The primer sequences are provided in Table S6.

### RT-PCR

Briefly, total RNA was isolated from cells and tissues using an RNeasy mini kit (Qiagen). cDNA was synthesized using a Verso cDNA Synthesis Kit (Thermo Fisher Scientific) according to the manufacturer’s instructions. The mRNA expression of target genes was analyzed by RT-PCR using specific primers. The PCR was conducted at 95°C for 3 min, followed by 25 cycles at 95°C for 30 s, 55°C for 30 s, and 72°C for 30 s, and a final extension at 72°C for 5 min, in a T100 PCR thermal cycler (BioRad). The primer sequences are provided in Table S7.

### Measurement of intracellular acetyl-CoA and α-ketoglutarate levels

*ARL6IP1* WT and KO MEFs (2–5 × 10^7^ cells/assay) at passage 3 were lysed in PBST via sonication and centrifuged at 13,000 × *g* at 4°C for 10 min. Cleared cell lysates were prepared from equal amounts of *ARL6IP1* WT and KO MEFs for the quantitative analysis of intracellular metabolites. These assays were performed using commercial acetyl-CoA (ab87546; Abcam) and α-ketoglutarate assay kits (ab83431; Abcam) according to the manufacturer’s instructions. The levels of intracellular acetyl-CoA and α-ketoglutarate were measured by converting a nearly colorless probe to a fluorescent product using a Spectra Max i3x microplate reader (Molecular Devices) at excitation and emission wavelengths of 535 and 587 nm, respectively. Detailed information regarding the assay kits used is provided in Table S3.

### Mitochondrial cholesterol assay

After isolation of mitochondrial lysates from *ARL6IP1* WT and KO MEFs (2–5 × 10^7^ cells/assay) at passage 3, mitochondrial cholesterol content was determined using a total cholesterol assay kit (STA-384; Cell Biolabs Inc.), according to the manufacturer’s instructions. Detailed information regarding the assay kit used is provided in Table S3.

### Measurement of ATP levels

Intracellular ATP levels in *ARL6IP1* WT and KO MEFs were determined using the Luminescent ATP Detection Assay Kit (ab1113849; Abcam), according to the manufacturer’s protocol. Briefly, MEFs at passage 3 were seeded at a density of 5 × 10^4^ cells/well in 24-well plates (SPL Life Sciences) for 24 h, lysed, and analyzed using a luminescence plate reader (GloMax Navigator; Promega). Detailed information regarding the assay kits used is provided in Table S3.

### MEF oxidative phosphorylation assay

Cellular respiration (oxygen consumption rate [OCR] and extracellular acidification rate [ECAR]) was assessed using an XFe24 Extracellular Flux analyzer (Seahorse Bioscience). Briefly, MEFs (4 × 10^5^ cells/well) at passage 2 were seeded in XF24 cell culture microplates (#102342-100, Seahorse XFe24 FluxPak mini; Seahorse Bioscience). After 24 h, the cells were incubated in 1 ml of XF DMEM supplemented with 10 mM glucose, 2 mM glutamine, and 1 mM sodium pyruvate for 1 h at 37°C in a non-CO_2_ incubator. The mitochondrial respiration was measured using oligomycin (1.5 μM), carbonyl cyanide-p-trifluoromethoxy phenylhydrazone (FCCP, 1 μM), and a mixture of rotenone/antimycin A (both 0.5 μM; #103015-100, XF Cell Mito Stress Test kit; Seahorse Bioscience) using an XFe24 analyzer.

### Mitophagy detection

Mitophagy was detected in *ARL6IP1* WT and KO MEFs using the Mitophagy Detection Kit (MD01-10; Dojindo Molecular Technologies), according to the manufacturer’s protocol. MEFs at passages 2–3 were cultured at a density of 3 × 10^4^ cells in a confocal dish (#101350; SPL Life Sciences) for 24 h and then incubated with 100 nM Mtphagy Dye at 37°C for 30 min. After washing with PBS, the cells were treated with 10 μM CCCP for 6 h, incubated with 1 μM Lyso Dye at 37°C for 30 min, and washed with PBS. Colocalization of Mtphagy and Lyso Dye was observed using the fluorescence microscope (IX71; Olympus) and CellSens imaging software (Olympus).

### Preparation for TEM

To confirm the myelin morphology of the spinal cord via TEM, WT or *ARL6IP1* KO mice (6-mo-old male, *n* = 4/group) were anesthetized by intraperitoneal injection with avertin (0.25 mg/g, T48402; Sigma-Aldrich) and perfused with ice-cold PBS until the liver became clear (∼30 min) and then with a fixation solution (2% paraformaldehyde [PFA], 2% glutaraldehyde in DPBS, pH 7.4) for 20 min at a low speed (2 ml/min). The spinal cord was dissected and fixed in the same solution overnight at 4°C. The fixed spinal cord was cut into 1-mm-thick sections, washed with ice-cold DPBS at 4°C, and fixed with 1% osmium tetroxide solution (Sigma-Aldrich) for 1 h at 4°C. To observe the contact surface between the mitochondria and ER, MEFs (3 × 10^6^ cells) were grown in 10-cm dishes (>90% confluency) and fixed with fixation solution (2% PFA, 2% glutaraldehyde in DPBS; pH 7.4) overnight at 4°C. Cells scraped into ice-cold PBS were harvested via centrifugation at 1,500 × *g* for 2 min. The pelleted cells were uniformly coated with 2% agarose via gentle vortexing and fixed with 1% osmium tetroxide solution for 1 h at 4°C.

Fixated cells and tissues were dehydrated with increasing concentrations of ethanol, infiltrated with propylene oxide, and embedded in EMbed-812 resin (EMS). Polymerization was performed at 60°C for 48 h and the sections were obtained using an ultracut EM UC7 ultramicrotome (Leica). The ultrathin sections (70 nm) were stained with uranyl acetate and Reynold’s lead citrate (Sigma-Aldrich), observed using FEI Tecnai G2 Spirit Twin TEM (FEI), and captured with NanoSprint 12 sCMOS camera (AMT Imaging). All images were obtained using ImageJ v1.57 (National Institutes of Health; NIH).

### Adenovirus and AAV production

For adenovirus production using the AdEasy system (Agilent), a recombinant adenovirus plasmid was obtained by homologous recombination between the pCMV-Shuttle vector (Agilent), encoding the target gene and the adenovirus genome vector in BJ5183 *E. coli* cells. Recombinant adenovirus packaging and amplification were performed in HEK293 cells (KCTC). For AAV production, we used a large-scale AAV9 packaging service and purified AAV9 for in vivo experiments from Vigene Biosciences.

### Determination of the AAV9 genome copy number using qPCR-based method

For the standard curve, circular and singly cut linear DNA plasmids were used as qPCR standards, and DNA concentrations were measured using Nanodrop spectrophotometers (N60; Implen GmbH). The plasmid was diluted to the range between 1 × 10^16^ and 1 × 10^23^ copies/ml for the qPCR standard curve. qPCR mixtures were prepared to a total volume of 20 µl with 2 µl of a template (plasmid DNA), 400 nM of each primer, and 10 µl of 2× Brilliant III SYBR Ultra-Fast qPCR Master Mix (Agilent) in a 96-well optical plate (Agilent). PCR was performed using EGFP and SV40 polyA primers according to the manufacturer’s instructions (AriaMx Real-Time PCR System; Agilent) as follows: an initial denaturation step at 95°C for 5 min, followed by 40 cycles of denaturation at 95°C for 15 s, and annealing or extension at 60°C for 30 s. The *R*^*2*^, slope, and intersection values were calculated from standard curves using the EGFP and the SV40 polyA primers. The primer sequences are provided in Table S8. For the AAV9 genome titer calculation from mouse tissues, all tissues (10–20 mg) were lysed in 180 µl ALT buffer supplemented with 20 µl Proteinase K (>600 mAU/ml) at 56°C until the tissue was completely digested. The lysates were subjected to extraction using the DNeasy Mini spin column according to the manufacturer’s instructions (DNeasy Blood & Tissue Kit; Qiagen). The genomic DNA concentration was measured and the AAV9 genome copy number was calculated using the qPCR method. The *R*^*2*^, slope, and intersection values are described in Table S9.

### Western blot analysis

Cells were lysed on ice using RIPA buffer (50 mM Tris-HCl pH 7.5, 150 mM NaCl, 0.5 mM EDTA, 1% NP-40, 0.1% SDS, 1 mM PMSF, and 1× protease inhibitor cocktail [Roche]). For protein extraction, tissues were placed in ice-cold PRO-PREP buffer (10–20 µl/mg tissue, #17081; Intron Biotech) containing protease inhibitor cocktail (#1183617001; Merck Millipore), homogenized, and treated with benzonase nuclease (E1014; Sigma-Aldrich). The whole-cell or tissue lysates were separated on 12 or 15% SDS-PAGE gel and then transferred onto PVDF membranes (Merck Millipore). The membranes were incubated with the primary antibodies in PBST (1× PBS, 0.05% Tween 20) overnight at 4°C. The membrane was then incubated with a secondary antibody in PBST-containing 0.5% skim milk for 1 h at 25°C. Proteins were visualized using a chemiluminescence kit (Immobilon; Merck Millipore).

### Protein microarray

Protein microarrays were performed using a HuProt human proteome microarray version 4.0 (HuProt; CDI Laboratories). The protein chip was blocked with 2% BSA in PBST (1× PBS with 0.1% Tween 20) on an orbital shaker at 4°C for 2 h. After washing three times with PBST, 9 μg His tagged ARL6IP1 was treated on the microarray and incubated overnight at 4°C. The microarray was incubated with 1 μg streptavidin-fluorescence (Alexa-Fluor 532) for 1 h at 4°C. After three washes with PBST, the array was centrifuged at 600 × *g* for 1 min for buffer removal. The microarray result was detected by scanning with an Axon GenePix 4000A Microarray Scanner (Molecular Devices) and acquired using GenePix Pro6.0 software (Molecular Devices).

### Glutathione S-transferase (GST)-pull-down and immunoprecipitation assays

Whole-cell lysates and reaction mixtures after the in vitro assay were incubated with glutathione Sepharose 4B beads (GE17-0756-01; Sigma-Aldrich) for 4 h at 4°C with gentle rotation. For immunoprecipitation assays, cells (3 × 10^6^ cells) were lysed in NET gel buffer (50 mM Tris-HCl; pH 7.5, 150 mM NaCl, 0.1% NP-40, and 1 mM EDTA; pH 8.0, containing protease inhibitor cocktails [#1183617001; Merck Millipore]) by sonication, and cell lysates cleared by centrifugation (at 15,000 × *g* at 4°C for 20 min) were incubated with 2 µg/reaction of anti-ARL6IP1, anti-LC3B, and anti-p62 antibodies in PBST overnight at 4°C and immobilized onto Protein A magnetic beads (10 µl/reaction; Thermo Fisher Scientific) for 2 h at 4°C with gentle rotation. The precipitates or bead-bound proteins were washed thoroughly with PBST three times and analyzed using western blotting.

### Purification of recombinant proteins expressed in *E. coli*

For the purification of GST fusion proteins, GST proteins were produced in BL21DE3 *E. coli*. Cells were grown at 37°C until an OD_600_ nm of 0.6 was reached, induced by adding 1 mM isopropyl-b-D-thiogalactoside (IPTG; Elpis Bio) for 3 h at 37°C. Cells were lysed in lysis buffer (1× PBS containing 1% Triton X-100, 1 mM PMSF) by sonication, centrifuged at 15,000 × *g* at 4°C for 20 min, and then the supernatants were incubated with glutathione Sepharose 4B (Sigma-Aldrich) for 3 h at 4°C. The bead–protein complexes were immediately washed with 5–10 column volumes of PBS and eluted in buffer (25 mM reduced glutathione in 10 mM Tris-HCl, 150 mM NaCl, and 1 mM PMSF; pH 7.5). Six His-tagged proteins were produced in BL21DE3 *E. coli* and purified following the manufacturer’s instruction (Qiagen). Bacterial cells were grown at 37°C to reach an OD_600_ nm of 0.6 and then induced by incubation with 1 mM IPTG for 24 h at 20°C. Cells were lysed in lysis buffer (10 mM imidazole in 50 mM NaH_2_PO_4_, 300 mM NaCl, and 1 mM PMSF; pH 8.0) by sonication, and cleared supernatants via centrifugation were incubated with Ni-NTA agarose (R90101; Qiagen) for 3 h at 4°C. The bead–protein complexes were washed in washing buffer (20 mM imidazole in 50 mM NaH_2_PO_4_, 300 mM NaCl, and 1 mM PMSF; pH 8.0) and eluted in elution buffer (250 mM imidazole in 50 mM NaH_2_PO_4_, 300 mM NaCl, and 1 mM PMSF; pH 8.0). Purified GST or His proteins were dialyzed in dialysis buffer (10 mM Tris-HCl, 10% glycerol, and 1 mM PMSF; pH 7.5) overnight at 4°C.

### In vitro binding assay

For the in vitro binding and pull-down assays, *ARL6IP1* and *LC3*-like genes were subcloned into pGEX4T1 and pET28a, respectively. The purified recombinant proteins from *E. coli* (each 0.5 μg protein/reaction) were incubated in PBST overnight at 4°C on a rotating platform. After incubation, the bound proteins were pulled down using glutathione Sepharose 4B beads (Sigma-Aldrich) for 3 h at 4°C, washed thrice with PBST, and analyzed using western blotting with the indicated antibodies.

### Iodixanol density gradient analysis

OptiPrep Density Gradient Medium (60% wt/vol iodixanol; D1556; Sigma-Aldrich) was diluted to 30% in ice-cold PBS, while 30% iodixanol solution (high density) was put in one chamber connected through a stopcock to a second chamber; 5% iodixanol solution (low density) was placed in the second chamber. The iodixanol solution flowed from the first chamber into the second, while a continuous gradient was formed by gravity in polypropylene centrifugation tubes (Hitachi). Cells were lysed on ice using RIPA buffer (500 μl per 5 × 10^6^ cells), and the whole-cell lysate was carefully layered on the top of the gradient and centrifuged at 100,000 *g* rpm at 4°C for 16 h. Fractions of density gradient layers (#1–#12) were collected and analyzed using western blotting.

Flotation analysis was performed as previously described (Nishimura et al., 2017). Briefly, *ARL6IP1* KO and WT MEFs (2 × 10^7^ cells) were harvested, lysed in lysis buffer (250 mM sucrose, 20 mM HEPES-KOH [pH 7.4], 1 mM EDTA containing protease inhibitor cocktail), and centrifuged at 7,000 × *g* for 10 min at 4°C. The supernatant was then mixed with an equal volume of OptiPrep (60% wt/vol iodixanol). Iodixanol gradients were generated using OptiPrep solution and lysis buffer and layered as follows: 2.2 ml at 30%, 2.2 ml at 25%, 2.2 ml at 20%, 1.8 ml at 15%, 1.8 ml at 10%. Additionally, 0.45 ml represented 5%, and 0.45 ml represented 0%. The gradients were centrifuged at 150,000 × *g* for 3 h at 4°C, and subsequently, 13 fractions (each 0.85 μl) were collected from the top and analyzed using western blotting.

### Bimolecular fluorescence complementation (BiFC) assay

The pBiFC-VN155 (#27097) and pBiFC-VC155 (#22011) plasmids were purchased from Addgene; recombinant plasmids encoding ARL6IP1 and ATGs were cloned into the pBiFC-VN155 or pBiFC-VC155 plasmids for the BiFC assay. Detailed information on the plasmid constructs is provided in Table S4 and Fig. S3 F. HeLa cells were seeded on confocal dishes (#101350; SPL Life Sciences) and transfected with the indicated combinations of pBiFC-VN155 and BiFC-VC155 vectors (each 0.5 μg per plasmid). At 24 h after transfection, the live cells were imaged with a confocal laser scanning microscope (LSM800; Zeiss).

### Annexin V-7-amino-actinomycin D (7-AAD) assay

Apoptosis was detected with Muse Annexin V & Dead Cell Reagent (MCH100105; Merck Millipore) using a Muse Cell Analyzer (Merck Millipore). ReNcell CX cells were transduced with adenovirus encoding ARL6IP1-shRNA or scrambled-shRNA at 200 multiplicity of infection (MOI) for 48 h and cultured in ReNcell NSC maintenance medium for 2 d. Cells were then harvested, stained with annexin V and 7-AAD according to the manufacturer’s instructions, and analyzed using a Muse Cell Analyzer. Apoptosis and cell death rates were reported as percentages of the total cell number.

### MEF senescence measurement

For measurement of MEF senescence, MEFs (2 × 10^4^ cells/well) at passages 3 and 5 were seeded in 96-well black-bottom plates coated with poly-L lysine (P4707; Sigma-Aldrich). After 24 h of incubation, the number of senescent cells was determined based on β-galactosidase activity using the Senescence β-Galactosidase Activity Assay Kit (Cell Signaling Technology), according to the manufacturer’s protocol. The fluorescence intensity (excitation and emission wavelengths = 360 and 465 nm, respectively) was detected using a Spectra Max i3x microplate reader (Molecular Devices) equipped with the Max Pro software.

### Detection of autophagy

Autophagy was determined using the Muse RFP-LC3 Reporter Autophagy Assay Kit and Muse Cell Analyzer (Merck Millipore). HeLa cells were seeded (2 × 10^5^ cells/well) in 6-well plates (SPL Life Sciences), and after 24 h, the cells were transduced with an adenovirus encoding ARL6IP1 or LacZ at 100 MOI for 48 h. The cells were harvested and the autophagy induction ratio was determined following the manufacturer’s instructions.

### MMP assay

MMP was determined using a JC-1 probe (T3168; Thermo Fisher Scientific) according to the manufacturer’s instructions. *ARL6IP1* WT and KO MEFs were seeded (5 × 10^4^ cells) in confocal dishes (#101350; SPL Life Sciences), treated with 10 µM CCCP (Sigma-Aldrich) for 6 h, and incubated with 1 μM JC-1 for 20 min at 37°C. JC-1 staining images were obtained using a fluorescence microscope (IX71; Olympus) and CellSens imaging software (Olympus).

MEFs were cultured in 96-well black bottom plates (Corning Inc.) coated with poly-L lysine overnight, treated with CCCP for 6 h, and incubated with 100 μl of 1 μM JC-1 per well for 20 min at 37°C. To quantify the monomers and aggregates of JC-1, the fluorescence was measured using a Spectra Max i3x microplate reader (Molecular Devices) at excitation and emission wavelengths of 485 and 535 nm, respectively, for JC-1 monomer and at 485 and 595 nm, respectively, for JC-1 aggregates.

### Visualization of mitochondrial calcium

The mitochondrial Ca^2+^ concentration was visualized using Rhod-2 AM (R1245MP; Thermo Fisher Scientific), a fluorescent calcium indicator. Briefly, *ARL6IP1* WT and KO MEFs at passage 3 were seeded (5 × 10^4^ cells) in a confocal dish (#101350; SPL Life Sciences), cultured for 24 h, and incubated with Rhod-2 AM (4.5 µM) and MitoTracker Green FM (20 nM; M7514; Thermo Fisher Scientific) in complete medium for 30 min at 37°C. After replacing with fresh medium, live cells were imaged using a fluorescence microscope (IX71; Olympus) and CellSens imaging software (Olympus).

### Immunofluorescence analysis

Cells or frozen tissue sections were fixed with 4% PFA overnight at 4°C, washed thrice in PBS, and permeabilized with 0.5% Triton X-100 in PBS for 15 min at 25°C. Thereafter, blocking was performed with 2% normal horse serum in PBS for 1 h at 25°C. Cells were incubated with primary antibodies, described as Table S2, in PBST for 16 h at 4°C, washed extensively with PBST three times, and incubated with the conjugated antibodies in PBST for 1 h at 25°C. For nuclear counterstaining, cells were incubated with DAPI solution (BD Biosciences), mounted with VectorMount AQ mounting medium (H-5501; Vector Laboratories), and observed under either a fluorescence microscope (IX71) or a confocal laser scanning microscope (LSM800). All images were quantitated using ImageJ v1.57 (NIH).

For immunofluorescence staining of primary rat hippocampal neurons, hippocampal neurons at 9 d after transfection were fixed, permeabilized, and incubated with anti-EGFP antibody and fluorophore-conjugated secondary antibodies (Jackson ImmunoResearch Laboratories, Inc.). Individual neurons were imaged using confocal laser scanning microscopy (LSM800) at 200×. Primary and secondary dendrites were quantified as previously described (Park et al., 2012).

### Isolation of MAM fraction

Isolation of mitochondria and MAM fractions from cells was performed as previously described (Wieckowski et al., 2009). Briefly, MEFs (5 × 10^8^ cells) at passages 2–3 were lysed in lysis buffer (225 mM mannitol, 75 mM sucrose, 0.1 mM EGTA, 30 mM, Tris-HCl pH 7.4, and 1× protease inhibitor cocktail) by sonication and centrifuged at 600 × *g* at 4°C for 5 min. The pellets were collected as the nuclear fraction. The supernatants were centrifuged at 10,000 × *g* at 4°C for 10 min to separate the crude mitochondrial fraction and the pellet containing the cytosolic/microsomal fractions. The supernatants were further centrifuged at 20,000 × *g* at 4°C for 1 h to collect the microsomal fractions. The crude mitochondrial fraction was resuspended in mitochondrial suspension buffer (250 mM mannitol, 5 mM HEPES pH 7.4, 0.5 mM EGTA, and 1× protease inhibitor cocktail) and separated via Percoll gradient ultracentrifugation to obtain the pure mitochondria and MAM fraction. The protein concentration from each subcellular fraction was measured via absorbance at 280 nm, calculated using a Nanodrop spectrophotometer, and analyzed using western blotting.

### Mouse behavior test

For footprint analysis, mice paws were put with nontoxic water-based ink (front paws in red and hind paws in blue ink), and subsequently, the mice were allowed to walk down an enclosed runway lined with white paper. The trials were repeated three to five times per mouse, and hind stride, hind sway, and stance length (the distance between the left and right forelimb stride) were measured in the middle part of each run.

### Mouse hindlimb clasping score measurement

Hindlimb reflexes were observed in the hindlimb position after grasping the tail for 10 s and scored on a scale of 0–3 with a degree of splaying outward from the abdomen. A score of 0 represents hindlimbs fully spread and moving; 1, a retraction toward the abdomen for >50% of one hindlimb; 2, a retraction toward the abdomen for >50% of both hindlimbs; and 3, both hindlimbs fully retracted to the abdomen.

### Mouse tissue collection and preparation

For the collection of the adult mouse brain or spinal cord tissues, mice were anesthetized by intraperitoneal injection using avertin (0.25 mg/g, T48402; Sigma-Aldrich) and perfused with ice-cold PBS to flush the blood and blood cells from the brain, followed by perfusion with 4% PFA solution for fixation. Thereafter, the organs were fixed in 4% PFA overnight at 4°C and immersed in 30% sucrose for 72 h for osmotic dehydration. Fixed tissues were embedded in Tissue-Tek OCT compound (#4583; Sakura Finetek), sectioned at 5 μm using a cryostat (CM1520; Leica), and analyzed using immunofluorescence staining with appropriate antibodies. Detailed information on antibodies used is provided in Table S2.

Individual mouse brain atlas was downloaded from the Allen Brain Atlas (http://atlas.brain-map.org), and reference images were generated from an adult mouse 3D corona averaged template.

### Mouse CSF and serum collection

Mice were anesthetized using avertin (0.25 mg/g, T48402; Sigma-Aldrich) and the CSF was immediately collected from the cisterna magna (5–10 μl CSF/25 g mouse). The pooled CSF samples were centrifuged at 7,500 × *g* for 5 min at 4°C; blood-contaminated samples were not analyzed. After CSF collection, blood samples (100 μl/25 g mouse) were collected from the hepatic portal vein and serum was obtained via centrifugation at 7,500 × *g* for 5 min at 4°C. All CSF and serum samples were aliquoted and stored at −80°C until use.

### Mouse cytokine array and measurement of neurofilament-L (NF-L) level

CSF and serum samples were analyzed using a proteome profiler mouse cytokine array kit (ARY006; R&D Systems) according to the manufacturer's instructions. NF-L level in CSF and serum (6 mo, male, *n* = 8/group) was measured using a Total Neurofilament-L Kit (#99175C; Cell Signaling Technology), according to the manufacturer’s instructions. Then absorbance was measured at 450 nm using a Spectra Max i3x microplate reader (Molecular Devices).

### Isolation of primary mouse cortical and rat hippocampal neurons

Cortical neurons were isolated from the E14.5 mouse brain and then dissociated by trypsinization (Trypsin Solution; Thermo Fisher Scientific). Clump tissues were removed by a 40-μm cell strainer, and cells were centrifuged at 150 × *g* for 2 min and then maintained in a neurobasal medium for 3 d (#21103049; Thermo Fisher Scientific) containing B-27 supplement (#17504044; Thermo Fisher Scientific) and MEM nonessential amino acid solution (#11140050, Thermo Fisher Scientific).

Primary hippocampal neurons were prepared as previously described (Lim et al., 2009). Hippocampi were dissected from the brains of E18 rats and dissociated physically with Pasteur pipettes after trypsin treatment. Neurons were grown on glass coverslips and transfected using the calcium phosphate method at 5 d in vitro.

### Sholl analysis of primary rat hippocampal neurons

Sholl analysis was performed employing the modified method as described previously (Nakayama et al., 2000). Individual neurons were imaged using a 200× objective, employing confocal microscopy, and the captured images were printed. To obtain Sholl profiles of dendritic arbors, printouts were placed under a transparent sheet featuring concentric circles with diameters increasing in 20-μm increments. The center of the circles was placed at the cell-body center and the number of dendrites crossing each concentric circle was counted. The means from multiple individual neurons were averaged to obtain a population mean and SEM.

### Stereotaxic injections with AAV9 virus

Mice were anesthetized by intraperitoneal injection using avertin (0.25 mg/g, T48402; Sigma-Aldrich) and fixed in a stereotaxic frame (David Kopf Instruments). The injection position was marked and the skull was drilled. Then, 1 μl AAV9 virus (2 × 10^13^ Vg/ml, each ARL6IP1 and GFP) per the primary motor cortex (M1) was delivered using an injection syringe (75RN, volume 5 μl; Hamilton). An injection was regulated by a syringe pump (Nanojet; Chemyx) at a constant volume of 0.2 μl/min (total of two sites/mouse), respectively. After the injection, the needle was kept in place for 5 min to minimize the upward flow of the virus solution. The coordinates for injection of the motor cortex region were calculated as anterior–posterior; −0.7 or −1.4, medial–lateral; +1.4, and dorsal–ventral; +1.6 relative to the bregma = 0.

### Statistical analysis

All statistical analyses were performed with GraphPad Prism 7.0 software. The Student’s *t* test was used to determine statistical significance. Data are represented as the mean ± standard deviation (*n* < 5) or mean ± SEM (*n* > 10).

### Online supplemental material

Fig. S1 shows the information of *Arl6ip1*^−/−^ mice model. Fig. S2 shows the neuropathological changes in the corticospinal tract of *Arl6ip1*^−/−^ mice. Fig. S3 shows the activation of the autophagy signal by ARL6IP1 via interaction with LC3B. Fig. S4 shows the identification of the interaction between BCL2L13 and ARL6IP1 in regulating autophagosome formation. Fig. S5 shows the change of neural cells in the primary motor cortex region of *Arl6ip1*^*−/−*^ or *Arl6ip1*^*+/+*^ mice after AAV9 gene delivery. Table S1 shows the summary of clinical features with ARL6IP1 mutations. Tables S2, S3, S4, S5, S6, S7, and S8 contain the lists of antibodies for western blot and immunofluorescence analysis, the lists of reagents a list of primers, and a list of plasmid constructs. Table S9 shows the calculation of R-square, slope, and intersection values of the qPCR. Table S10 shows the titration of the AAV9 genome copy number using SV40 primer to mouse tissues. Videos 1 and 2 show the gait of 9-mo-old *Arl6ip1*^*−/−*^ mice versus *Arl6ip1*^*+/+*^ mice, respectively.

### Ethical approval and consent to participate

All animal housing and experiments conducted were in accordance with the KRIBB Institutional Animal Care and Use Committee Guidelines (KRIBB-AEC-18090). The study involved no human participants.

## Supplementary Material

Table S1 provides a summary of clinical features with ARL6IP1 mutations.

Table S2 lists the antibodies used in this study.

Table S3 lists reagents and kits list used in this study

Table S4 shows the primer list for genotyping of C57BL/6N-Arl6ip1(tm1a) mice.

Table S5 lits plasmid constructs used in the study.

Table S6 shows the primer list for RT-qPCR.

Table S7 shows the primer list for conventional RT-PCR.

Table S8 shows the primer sets used in qPCR for titration of AAV genome copy numbers.

Table S9 lists R-square, slope, and intersection values of the qPCR.

Table S10 lists titration of AAV9 genome copy number using SV40 primer to mouse tissues.

SourceData F2 is the source file for Fig. 2.

SourceData F3 is the source file for Fig. 3.

SourceData F4 is the source file for Fig. 4.

SourceData F5 is the source file for Fig. 5.

SourceData F7 is the source file for Fig. 7.

SourceData FS1 is the source file for Fig. S1.

SourceData FS2 is the source file for Fig. S2.

SourceData FS3 is the source file for Fig. S3.

SourceData FS4 is the source file for Fig. S4.

SourceData FS5 is the source file for Fig. S5.

## Data Availability

The data that supports the findings of this study are available in the supplementary material and provided source data of this article. Contact for reagent and resource sharing; Jung Hwa Lim, jhwa@kribb.re.kr or Cho-Rok Jung, crjung@kribb.re.kr.
