# Supplementary material for: ARL6IP1 gene delivery reduces neuroinflammation and neurodegenerative pathology in hereditary spastic paraplegia model
Source: J Exp Med. 2023 Nov 7;221(1):e20230367. doi: 10.1084/jem.20230367 (PMC10630151; doi:10.1084/jem.20230367)
Supplement: Table S1 — provides a summary of clinical features with ARL6IP1 mutations. [file JEM_20230367_TableS1.docx]

Table S1. Summary of clinical features with ARL6IP1 pathogenic mutations

| **Disease** | **Gene Name** | **Ethnicity** | **Age of onset** | **Nucleotide**  **change** | **Protein**  **change** | **Family**  **history** | **Clinical phenotype** | **Ref.** |
| --- | --- | --- | --- | --- | --- | --- | --- | --- |
| Hereditary Spastic paraplegia  (HSP) | *ARL6IP1* | Middle East | 14 months | c.576_579delAAAC,  homozygous | **p.K193Ffs36X** | AR | spastic paraplegia Objective sensory loss diffuse sensory and motor polyneuropathy acromutilation Loss of terminal digits and acropathy | Novarino et al., 2014^1)^ |
| Hereditary sensory and autonomic neuropathies (HSAN) type II | *ARL6IP1* | Caucasian | <12 months | c.577_580delAACT,  homozygous | **p.K193FfsX37** | AR | congenital insensitivity to pain acromutilation spastic paraplegia Loss of terminal digits and acropathy | Nizon et al., 2018^2)^ |
| Hereditary Spastic paraplegia  (HSP) | *ARL6IP1* | Russian | 3 months | c.92T>C,  homozygous | **p.L31P** | AR | Inability to sit or walk without assistance  At the age of 3, signs of developmental regression (inability of head control, chewing diﬃculties, drooling)  At the age of 6, muscle weakness, hypotrophy, spastic tetraparesis, increased patellar and ulnar reﬂexes, myoclonic episode (hands jerking), hand tremor, extensor plantar responses,  oral automatisms and intellectual disability | Chukhrova et al., 2019^3)^ |
| Hereditary Spastic paraplegia  (HSP) | *ARL6IP1* | Middle East | Birth | c.112C>T,  homozygous | **p.R38X** | AR | Respiratory distress (ventilation dependent)  Severe development delay  Motor neuropathy  Muscle weakness and wasting of the calf muscle  Severe lumbar  Hyperlordosis  Microcephaly  Died from cardiac arrest at 28 months | Wakil et al., 2019^4)^ |
| Hereditary Spastic paraplegia  (HSP) | *ARL6IP1* | Mexican | <4 weeks | c.346C>T,  homozygous | **p.R116X** | AR | Respiratory distress (ventilation dependent)  Extreme development delay (head lag, no social)  Denervation, atrophy with rare areas of reinnervation  Died at 4 months | Cao et al., 2020^5)^ |
| Hereditary Spastic paraplegia  (HSP) | *ARL6IP1* | Mexican | 3 weeks | c.346C>T,  homozygous | **p.R116X** | AR | Respiratory distress (ventilation dependent)  Amyelinating neuropathy | Cao et al., 2020^5)^ |

^1)^ Novarino, Gaia, et al. "Exome sequencing links corticospinal motor neuron disease to common neurodegenerative disorders." science 343.6170 (2014): 506-511.

^2)^ Nizon, M., et al. "ARL6IP1 mutation causes congenital insensitivity to pain, acromutilation and spastic paraplegia." Clinical Genetics 93.1 (2018): 169-172.

^3)^ Chukhrova, A. L., et al. "A new case of infantile‐onset hereditary spastic paraplegia with complicated phenotype (SPG 61) in a consanguineous Russian family." European Journal of Neurology 26.5 (2019): e61-e62.

^4)^ Wakil, Salma M., et al. "Truncating ARL6IP1 variant as the genetic cause of fatal complicated hereditary spastic paraplegia." BMC Medical Genetics 20.1 (2019): 1-6.

^5)^ Ninmer, E. K., et al. "Necrotizing enterocolitis totalis complicates an infantile presentation of ARL6IP1-related spastic paraplegia 61." Journal of Pediatric Surgery Case Reports 75 (2021): 102063.
