# Supplementary material for: ARL6IP1 gene delivery reduces neuroinflammation and neurodegenerative pathology in hereditary spastic paraplegia model
Source: J Exp Med. 2023 Nov 7;221(1):e20230367. doi: 10.1084/jem.20230367 (PMC10630151; doi:10.1084/jem.20230367)
Supplement: Table S2 — lists the antibodies used in this study. [file JEM_20230367_TableS2.docx]

Table S2. Antibody list used in this study

| **Antibodies** | **Company** | **Catalogue No.** | **Clone No.** | **Dilution for western blot** | **Dilution for IF** |
| --- | --- | --- | --- | --- | --- |
| **Primary Antibodies** | | | | | |
| β actin | Sigma-Aldrich | A1978 | AC-15 | 1:5000 | NA |
| LC3B | Sigma-Aldrich | L7543 | polyclonal | 1:5000 | 1:500 |
| ATG5  (ATG5-ATG12 conjugate) | Santa-Cruz | sc-133158 | C-1 | 1:2000 | NA |
| BAX | Santa-Cruz | sc-70407 | 4H32 | 1:2000 | NA |
| Bcl-2 | Santa-Cruz | sc-7382 | C-2 | 1:2000 | NA |
| GST | Santa-Cruz | sc-138 | B-14 | 1:2000 | NA |
| LAMP-1 | Santa-Cruz | sc-20011 | H4A3 | NA | 1:200 |
| SQSTM1/p62 (rodent) | Cell signaling | #23214 | D6M5X | 1:2000 | NA |
| SQSTM1/ P62 | Santa-Cruz | sc-28359 | D-3 | 1:2000 | NA |
| PCNA | Santa-Cruz | sc-56 | PC10 | 1:5000 | NA |
| PUMA α/β | Santa-Cruz | sc-374223 | G-3 | 1:2000 | NA |
| Atlastin (SPG3) | Santa-Cruz | sc-376619 | E-9 | 1:2000 | NA |
| ARMER(ARL6IP1) | Santa-Cruz | sc-514227 | E-8 | 1:2000 | NA |
| IbaI | Invitrogen | MA5-27726 | GT10312 | NA | 1:500 |
| ATG5 | Cell signaling | #12994 | D5F5U | 1:3000 | NA |
| Beclin-1 | Cell signaling | #3495 | D40C5 | 1:2000 | NA |
| Caspase-3 | Cell signaling | #9662 | polyclonal | 1:3000 | NA |
| Cleaved caspase-3 | Cell signaling | #9661 | 5A1E | 1:1000 | NA |
| Caspase-9 | Cell signaling | #9508 | C9 | 1:2000 | NA |
| GFAP | Cell signaling | #3670 | GA5 | 1:2000 | 1:100 |
| GM130 | Cell signaling | #12480 | D6B1 | NA | 1:100 |
| His tag | Cell signaling | #2365 | polyclonal | 1:3000 | NA |
| IP3R | Cell signaling | #8568 | D53A5 | 1:2000 | NA |
| MAP2 | Cell signaling | #4542 | polyclonal | 1:2000 | 1:200 |
| MBP | Cell signaling | #78896 | D8X4Q | 1:5000 | 1:100 |
| MOG | Cell signaling | #45268 | D5B4C | 1:2000 | 1:100 |

*NA; not applicable

Table S2. Antibody list used in this study (continued)

| **Antibodies** | **Company** | **Catalogue No.** | **Clone No.** | **Dilution for western blot** | **Dilution for IF** |
| --- | --- | --- | --- | --- | --- |
| **Primary Antibodies** | | | | | |
| Sec61B | Cell signaling | #14648 | D5Q1W | 1:5000 | 1:500 |
| NeuN | Cell signaling | #24309 | D4G40 | 1:3000 | 1:100 |
| Neurofilament light chain | Cell signaling | #2835 | DA2 | 1:3000 | 1:100 |
| Tom20 | Cell signaling | #42406 | D8T4N | 1:3000 | NA |
| α-tubulin | Cell signaling | #2125 | 11H10 | 1:3000 | NA |
| ULK1 | Cell signaling | #8504 | D8H5 | 1:2000 | NA |
| Phospho-ULK1 (Ser555) | Cell signaling | #5869 | D1H4 | 1:1000 | NA |
| Phospho-ULK1 (Ser757) | Cell signaling | #14202 | D7O6U | 1:2000 | NA |
| FIP200 | Cell signaling | #12436 | D10D11 | 1:2000 | NA |
| PI3 Kinase Class III | Cell signaling | #4263 | D9A5 | 1:2000 | NA |
| Bif-1 | Cell signaling | #4467 | polyclonal | 1:1000 | NA |
| ATG9A | Cell signaling | #13509 | D4O9D | 1:1000 | NA |
| WIPI2 | Cell signaling | #8567 | polyclonal | 1:1000 | 1:100 |
| BCL2L13 | Cell signaling | # 61974 | E6G1Q | 1:2000 | NA |
| CD40 | Cell signaling | #86165 | E2Z7J | NA | 1:100 |
| Arginase-1 | Cell signaling | #93668 | D4E3M | NA | 1:100 |
| GFP | Roche | 1181446001 | Clone 7.1,13.1 | NA | 1:500 |
| ARL6IP1  (discontinued) | abcam | ab24228 | polyclonal | 1:3000 | 1:200 |
| Calnexin | abcam | ab22595 | polyclonal | 1:5000 | 1:500 |
| Cytochrome C | abcam | ab90529 | polyclonal | NA | 1:200 |
| REEP1 (SPG31) | abcam | ab105583 | polyclonal | 1:2000 | NA |
| VDAC-1 | abcam | ab14734 | 20B12AF2 | 1:2000 | 1:100 |

*NA; not applicable

| **Antibodies** | **Company** | **Catalogue No.** | **Dilution for western blot** | **Dilution for IF** |
| --- | --- | --- | --- | --- |
| **Secondary Antibodies** | | | | |
| m-IgGκ BP-HRP | Santa-Cruz | sc516102 | 1:10000 | NA |
| Anti-mouse IgG-HRP | Cell signaling | #7076 | 1:10000 | NA |
| Anti-rabbit IgG-HRP | Cell signaling | #7074 | 1:10000 | NA |
| Donkey anti-Mouse IgG, Alexa Fluor 488 | Invitrogen | A21202 | NA | 1:1000 |
| Donkey anti-Rabbit IgG, Alexa Fluor 488 | Invitrogen | A21206 | NA | 1:1000 |
| Goat anti-Mouse IgG, Alexa Fluor 546 | Invitrogen | A11003 | NA | 1:1000 |
| Goat anti-Rabbit IgG, Alexa Fluor 546 | Invitrogen | A11010 | NA | 1:1000 |

Table S2. Antibody list used in this study (continued)

*NA; not applicable
