# Supplementary material for: ARL6IP1 gene delivery reduces neuroinflammation and neurodegenerative pathology in hereditary spastic paraplegia model
Source: J Exp Med. 2023 Nov 7;221(1):e20230367. doi: 10.1084/jem.20230367 (PMC10630151; doi:10.1084/jem.20230367)
Supplement: Table S3 — lists reagents and kits list used in this study [file JEM_20230367_TableS3.docx]

Table S3. Reagents and kits list used in this study

| **Reagents** | **Company** | **Catalogue No.** |
| --- | --- | --- |
| Pierce™ Protein A Magnetic Beads | Thermo Scientific | 88845 |
| Ni-NTA agarose | QIAGEN | Ni-NTA resin |
| Glutathione Sepharose® 4B | Sigma-Aldrich | GE17-0756-01 |
| OptiPrep™ Density Gradient Medium | Sigma-Aldrich | D1556 |
| Percoll®, pH 8.5-9.5 (20 °C) | Sigma-Aldrich | P1644 |
| Carbonyl cyanide 3-chlorophenylhydrazone (CCCP) | Sigma-Aldrich | C2759 |
| Wortmannin | Sigma-Aldrich | W1628 |
| DAPI solution | BD Biosciences | #564907 |
| JC-1 Dye  (Mitochondrial Membrane Potential Probe) | Invitrogen | T3168 |
| MitoTracker™ Deep Red FM | Invitrogen | M22426 |
| MitoTracker™ Green FM | Invitrogen | M7514 |
| Rhod-2, AM, cell permeant | Invitrogen | R1244 |
| Sudan Black B | Sigma-Aldrich | 86015 |
| Muse™ Annexin V & Dead Cell Reagent | Merck | MCH100105 |
| Muse™RFP-LC3 Reporter Autophagy Assay Kit | Merck | MCH200110 |
| Senescence β-Galactosidase Activity Assay Kit | Cell signaling | 23833 |
| Proteome Profiler Mouse Cytokine Array Kit, Panel A | R&D systems | ARY006 |
| Total Neurofilament-L kit | Cell signaling | #99175C |
| Luminescent ATP detection assay | abcam | ab1113849 |
| Alpha ketoglutarate assay kit | abcam | ab83431 |
| Acetyl-CoA assay kit | abcam | ab87546 |
| Total cholesterol assay kit | Cell biolabs | STA-384 |
| Seahorse XFe24 Flux Pak  Seahorse XF Cell Mito Stress Test Kit | Agilent | 102342-100  103015-100 |
