# Supplementary material for: ARL6IP1 gene delivery reduces neuroinflammation and neurodegenerative pathology in hereditary spastic paraplegia model
Source: J Exp Med. 2023 Nov 7;221(1):e20230367. doi: 10.1084/jem.20230367 (PMC10630151; doi:10.1084/jem.20230367)
Supplement: Table S4 — shows the primer list for genotyping of C57BL/6N-Arl6ip1(tm1a) mice. [file JEM_20230367_TableS4.docx]

Table S4. The primer list for genotyping of C57BL/6N-Arl6ip1^(tm1a)^ mice

| **Gene name** | **Primer** | **Primer sequences (5'-3')** | **AT (℃)** |
| --- | --- | --- | --- |
| *Arl6ip1*  *WT allele* | 5arm-WTF | GGC TGC ATT TGG GGA TAT TA | 60 |
|  | Crit-WTR | TCA ACA CTT TGG TGG CTC AG | 60 |
| *Arl6ip1*  *MT allele* | 5arm-WTF | GGC TGC ATT TGG GGA TAT TA | 60 |
|  | 5 mut-R1 | GAA CTT CGG AAT AGG AAC TTC G | 60 |

https://archive.har.mrc.ac.uk/harwell_archive/live_files/7237/HA_7237_Genotyping_Protocols.pdf
