# Supplementary material for: ARL6IP1 gene delivery reduces neuroinflammation and neurodegenerative pathology in hereditary spastic paraplegia model
Source: J Exp Med. 2023 Nov 7;221(1):e20230367. doi: 10.1084/jem.20230367 (PMC10630151; doi:10.1084/jem.20230367)
Supplement: Table S5 — lits plasmid constructs used in the study. [file JEM_20230367_TableS5.docx]

Table S5. Plasmid constructs used in the study

| **Plasmid** | **Construction** |
| --- | --- |
| pShuttle-CMV-ARL6IP1 | ARL6IP1 full-length was amplified by RT-PCR using cDNA from SH-SY5Y cells and cloned into the XhoI/HindIII sites of pShuttle-CMV |
| pSuper-ARL6IP1 shRNA | ARL6IP1-shRNA (5’-CTCCTTGGAAAGCTTCTTA-3’) was inserted between the cloning sites (BglII and HindIII) into the pSuper (H1 promoter) |
| pFlag-ARL6IP1 | ARL6IP1 full-length was amplified by RT-PCR using cDNA from HeLa cells and cloned into the EcoRI/ SalI sites of pFLAG-CMV2 |
| pET28a-ARL6IP1 | ARL6IP1 full-length was amplified from pFlag-ARL6IP1 and cloned into the EcoRI/ SalI sites of the pET28a |
| pET28a-LC3A | LC3A full-length was amplified by RT-PCR using cDNA from HeLa cells and cloned into the EcoRI/ XhoI sites of pET28a |
| pET28a-LC3B | LC3B full-length was amplified by RT-PCR using cDNA from HeLa cells and cloned into the EcoRI/ XhoI sites of pET28a |
| pET28a-LC3C | LC3C full-length was amplified by RT-PCR using cDNA from HeLa cells and cloned into the EcoRI/ XhoI sites of pET28a |
| pET28a-GABARAP-L1 | GABARAP-L1 full-length was amplified by RT-PCR using cDNA from HeLa cells and cloned into the EcoRI/ XhoI sites of pET28a |
| pET28a- GABARAP-L2 | GABARAP-L2 full-length was amplified by RT-PCR using cDNA from HeLa cells and cloned into the EcoRI/ XhoI sites of pET28a |
| pGEX4T1-ARL6IP1_FL | ARL6IP1 full-length was digested with BamHI and NotI from pET28a-ARL6IP1and cloned into the pGEX4T1 |
| pGEX4T1-ARL6IP1_NT | ARL6IP1 fragment (1-44a.a) was amplified from pFlag-ARL6IP1 and cloned into the BamHI/ NotI sites of pGEX4T1 |
| pGEX4T1-ARL6IP1_ΔNT1 | ARL6IP1 fragment (45-203a.a) was amplified from pFlag-ARL6IP1 and cloned into the BamHI/ NotI sites of pGEX4T1 |
| pGEX4T1-ARL6IP1_ΔNT2 | ARL6IP1 fragment (88-203a.a) was amplified from pFlag-ARL6IP1 and cloned into the BamHI/ NotI sites of pGEX4T1 |
| pGEX4T1-ARL6IP1_ΔNT3 | ARL6IP1 fragment (139-203a.a) was amplified from pFlag-ARL6IP1 and cloned into the BamHI/ NotI sites of pGEX4T1 |
| pGEX4T1-LC3B | LC3B full-length was digested with EcoRI and XhoI from pET28a-LC3B and cloned into the pGEX4T1 |
| pAAV-CAG-GFP | The pAAV-CAG-GFP construct was purchased from Addgene.  (Plasmid #37825) |
| pAAV-CAG-ARL6IP1_GFP  (mouse origin) | ARL6IP1 full-length was amplified by RT-PCR using cDNA from Neuro-2a cells and cloned into the XbaI/ BamHI sites of pAAV-CAG-GFP |
| pEGFP-ARL6IP1 | ARL6IP1 full-length was amplified from pFlag-ARL6IP1 and cloned into the BamHI/ EcoRI sites of pEGFP-C2 |

Table S5. Plasmid constructs used in the study (continued)

| **Plasmid** | **Construction** |
| --- | --- |
| pBiFC-VN155  Venus 1-154a.a, I152L | The pBiFC-VN155 construct was purchased from Addgene.  (Plasmid #27097) |
| pBiFC-VC155  Venus 155-238a.a, A206K | The pBiFC-VC155 construct was purchased from Addgene.  (Plasmid #22011) |
| pBiFC-VN155-ARL6IP1 | ARL6IP1 full-length was amplified from pFlag-ARL6IP1 and cloned into the EcoRI/ KpnI sites of pBiFC-VN155 |
| pBiFC-VC155-BECN1 | ARL6IP1 full-length was amplified from pFlag-ARL6IP1 and cloned into the EcoRI/ KpnI sites of pBiFC-VC155 |
| pBiFC-VC155-ATG5 | ATG5 full-length was amplified from pFlag-ARL6IP1 and cloned into the SalI/ KpnI sites of pBiFC-VC155 |
| pBiFC-VC155-ATG4B | ARL6IP1 full-length was amplified from pFlag-ARL6IP1 and cloned into the EcoRI/ KpnI sites of pBiFC-VC155 |
| pBiFC-VC155-ATG7 | ATG7 full-length was amplified from pFlag-ARL6IP1 and cloned into the EcoRI/ KpnI sites of pBiFC-VC155 |
| pBiFC-VC155-LC3B | ARL6IP1 full-length was amplified from pFlag-ARL6IP1 and cloned into the EcoRI/ KpnI sites of pBiFC-VC155 |
| pBiFC-VC155-p62 | ARL6IP1 full-length was amplified from pFlag-ARL6IP1 and cloned into the EcoRI/ KpnI sites of pBiFC-VC155 |
| pcDNA3.1-mCherry-ARL6IP1 | ARL6IP1 full-length was amplified from pFlag-ARL6IP1 and cloned into the EcoRI/ XhoI sites of pcDNA3.1-mCherry |
| ARMER CRISPR/Cas9 | The ARMER CRISPR/Cas9 Plasmids construct was purchased from Santa-cruz.(Plasmid sc-409837) |
| ARMER HDR | The ARMER HDR Plasmids construct was purchased from Santa-cruz.  (Plasmid sc-409837-HDR) |
