# Supplementary material for: ARL6IP1 gene delivery reduces neuroinflammation and neurodegenerative pathology in hereditary spastic paraplegia model
Source: J Exp Med. 2023 Nov 7;221(1):e20230367. doi: 10.1084/jem.20230367 (PMC10630151; doi:10.1084/jem.20230367)
Supplement: Table S6 — shows the primer list for RT-qPCR. [file JEM_20230367_TableS6.docx]

Table S6. The primer list for RT-qPCR

| **Gene categories** | | | **Genes** | | | **Species** | | | **Forward 5’–3’** | | | **Reverse 5’–3’** | **Genebank Assession No.** | | |  |  |
| --- | --- | --- | --- | --- | --- | --- | --- | --- | --- | --- | --- | --- | --- | --- | --- | --- | --- |
| *Target gene* | | | *ARL6IP1* | | | Homo sapiens | | | GAGATAATCGCAGCACCAAC | | | CACCAAAGAAACCACACCC | NM_015161.3 | | |  |  |
| *Housekeeping gene* | | | *GAPDH* | | | Homo sapiens | | | GAGTCAACGGATTTGGTCGT | | | TTGATTTTGGAGGGATCTCG | NM_002046 | | |  |  |
| *Target gene* | | | *Arl6ip1* | | | Mus musculus | | | GGATAACCGCAGCAGCAAC | | | ACCACACCCATGATGGCAG | NM_019419 | | |  |  |
| *Housekeeping gene* | | | *Gapdh* | | | Mus musculus | | | AACTTTGGCATTGTGGAAGG | | | ACACATTGGGGGTAGGAACA | NM_001289726 | | |  |  |
| Pan-astrocyte | | | *Aqp4* | | | Mus musculus | | | CTCCCTTTGCTTTGGACTCA | | | CGATGCTGATCTTTCGTGTG | NM_009700 | | |  |  |
| Pan-astrocyte | | | *Gfap* | | | Mus musculus | | | GCTCCAAGATGAAACCAACC | | | CCTCCTCCAGCGATTCAAC | NM_001131020 | | |  |  |
| A1 Astrocyte | | | *Gbp2* | | | Mus musculus | | | GGAGGAGCTGTGTGGTGAAT | | | TTAGACGTGGCCCATTGACT | NM_010260 | | |  |  |
| A1 Astrocyte | | | *H2-T23* | | | Mus musculus | | | TGATCATCCTTGGAGCTGTG | | | TTCTGAGGCCAGTCAGAGGT | NM_010398 | | |  |  |
| A1 Astrocyte | | | *H2-D1* | | | Mus musculus | | | GTTGCTGTTCTGGGTGTCCT | | | CCTGGAGCCAGAGCATAGTC | NM_010380 | | |  |  |
| A2 Astrocyte | | | *S100a10* | | | Mus musculus | | | GTGCTCATGGAACGGGAGT | | | AAAGCTCTGGAAGCCCACTT | NM_009112 | | |  |  |
| A2 Astrocyte | | | *Tgm1* | | | Mus musculus | | | CCCTGGATGACAATGGAGTT | | | GAATAGCCGGTGCGTAGGTA | NM_001161715 | | |  |  |
| A2 Astrocyte | | | *Ptx3* | | | Mus musculus | | | TTCCTGAGGGTGGACTCCTA | | | CCGATCCCAGATATTGAAGC | NM_008987 | | |  |  |
| Microglia | | | *Iba1*  *(Aif1)* | | | Mus musculus | | | GAAGCGAATGCTGGAGAAAC | | | AGTCAGAGTAGCTGAACGTC | NM_001361501 | | |  |  |
| Microglia and M1 Macrophage | | | *Cx3cr1* | | | Mus musculus | | | AGCTCACGACTGCCTTCTTC | | | GTCCGGTTGTTCATGGAGTT | NM_009987 | | |  |  |
| **Gene categories** | | **Gene** | | | **Species** | | | **Forward 5’–3’** | | | **Reverse 5’–3’** | | | | **Genebank Assession No.** | | |
| M1 Macrophage | | *Cd40* | | | Mus musculus | | | GCACCGCAGAATCAGACAC | | | CCATCTCCATAACTCCAAAGCC | | | | NM_011611.2 | | |
| M1 Macrophage | | *Cd80* | | | Mus musculus | | | AAAACCCCCAGAAGACCC | | | GACAACGATGACGACGAC | | | | NM_001359898 | | |
| M2 Macrophage | | *Arg-1* | | | Mus musculus | | | CGCCTTTCTCAAAAGGACAG | | | CCAGCTCTTCATTGGCTTTC | | | | NM_007482 | | |
| M2 Macrophage | | *Ym-1*  *(Chil3)* | | | Mus musculus | | | GGGCATACCTTTATCCTGAG | | | CCACTGAAGTCATCCATGTC | | | | NM_009892 | | |
| M2 Macrophage | | *Cd163* | | | Mus musculus | | | CAGACTGGTTGGAGGAGAAATC | | | TGACTT GTCTCTGGAAGCTG | | | | NM_001170395 | | |
| M2 Macrophage | | *Igf-1* | | | Mus musculus | | | TGGATGCTCTTCAGTTCGTG | | | CACAATGCCTGTCTGAGGTG | | | | NM_010512 | | |
| Premyelinating Oligodendrocyte | | *Olig1* | | | Mus musculus | | | TCATCCTCATCCTCATCCTCTTCC | | | GCTGCTGCTGTTCCTCTTTGG | | | | NM_016968 | | |
| Premyelinating Oligodendrocyte | | *Olig2* | | | Mus musculus | | | GGCGGTGGCTTCAAGTCATC | | | TCGGGCTCAGTCATCTGCTTC | | | | NM_016967 | | |
| Mature Oligodendrocyte | | *Plp1* | | | Mus musculus | | | TGCTCGGCTGTACCTGTGTACATT | | | TACATTCTGGCATCAGCGCAGAGA | | | | NM_011123 | | |
| Mature Oligodendrocyte | | *Mbp* | | | Mus musculus | | | TCACAGAAGAGACCCTCACA | | | GCCGTAGTGGGTAGTTCTTG | | | | NM_001025251 | | |
| Inflammation related genes | | *Timp-1* | | | Mus musculus | | | CCAGAACCGCAGTGAAGAG | | | ACGCCAGGGAACCAAGAAG | | | | NM_001044384 | | |
| Inflammation related genes | | *Icam-1 (Cd54)* | | | Mus musculus | | | CCGCTACCATCACCGTGTATTC | | | GTCCTTGCCTACTTGCTGCC | | | | NM_010493 | | |
| Inflammation related genes | | *Il-1β* | | | Mus musculus | | | GCAGCAGCACATCAACAAG | | | ACGGGAAAGACACAGGTAG | | | | NM_008361 | | |
| Inflammation related genes | | *IL-6* | | | Mus musculus | | | TCTGCAAGAGACTTCCATCC | | | TCCACGATTTCCCAGAGAAC | | | | NM_031168 | | |
| Inflammation related genes | | *TNF-α* | | | Mus musculus | | | AACTGGCAGAAGAGGCAC | | | CAAGCAGGAATGAGAAGAGG | | | | NM_013693.3 | | |
| **Gene categories** | **Gene** | | | **Species** | | | **Forward 5’–3’** | | | **Reverse 5’–3’** | | | | **Genebank Assession No.** | | |  |
| Inflammation related genes | *iNOS*  *(Nos2)* | | | Mus musculus | | | CAGCTGGGCTGTACAAACCTT | | | CATTGGAAGTGAAGCGTTTCG | | | | NM_010927 | | |  |
| Inflammation related genes | *Cox2*  *(Ptgs2)* | | | Mus musculus | | | TGCACTATGGTTACAAAAGCTGG | | | TCAGGAAGCTCCTTATTTCCCTT | | | | NM_011198 | | |  |
| Oxidation related Genes | *CuZn-Sod*  *(Sod1)* | | | Mus musculus | | | AACCAGTTGTGTTGTCAGGAC | | | CCACCATGTTTCTTAGAGTGAGG | | | | NM_011434 | | |  |
| Oxidation related Genes | *Mn-Sod*  *(Sod2)* | | | Mus musculus | | | AGACCTGCCTTACGACTATGG | | | CTCGGTGGCGTTGAGATTGTT | | | | NM_013671 | | |  |
| Oxidation related Genes | *Ho1*  *(Hmox1)* | | | Mus musculus | | | GATAGAGCGCAACAAGCAGAA | | | CAGTGAGGCCCATACCAGAAG | | | | NM_010442 | | |  |
| Oxidation related Genes | *Nrf2*  *(Nfe2l2)* | | | Mus musculus | | | TAGATGACCATGAGTCGCTTGC | | | GCCAAACTTGCTCCATGTCC | | | | NM_010902 | | |  |
| Oxidation related Genes | *Nqo1* | | | Mus musculus | | | AGGATGGGAGGTACTCGAATC | | | TGCTAGAGATGACTCGGAAGG | | | | NM_008706 | | |  |

https://eurofinsgenomics.eu/en/ecom/tools/qpcr-assay-design/
