# Supplementary material for: ARL6IP1 gene delivery reduces neuroinflammation and neurodegenerative pathology in hereditary spastic paraplegia model
Source: J Exp Med. 2023 Nov 7;221(1):e20230367. doi: 10.1084/jem.20230367 (PMC10630151; doi:10.1084/jem.20230367)
Supplement: Table S7 — shows the primer list for conventional RT-PCR. [file JEM_20230367_TableS7.docx]

Table S7. The primer list for conventional RT-PCR

| **Gene name** | **Species** | **Forward primer (5'-3')** | **Reverse primer (5'-3')** | **Genebank Assession No.** | **AT (℃)** |
| --- | --- | --- | --- | --- | --- |
| *Arl6ip1* | Mus musculus | TGC TGA TGG CTG ACA AAG TC | ACC GCA GCA AGA GAA ATG AT | NM_019419 | 57 |
| *Gapdh* | Mus musculus | AAC TTT GGC ATT GTG GAA GG | ACA CAT TGG GGG TAG GAA CA | NM_001289726 | 55 |

https://bioinfo.ut.ee/primer3-0.4.0/
