# Supplementary material for: ARL6IP1 gene delivery reduces neuroinflammation and neurodegenerative pathology in hereditary spastic paraplegia model
Source: J Exp Med. 2023 Nov 7;221(1):e20230367. doi: 10.1084/jem.20230367 (PMC10630151; doi:10.1084/jem.20230367)
Supplement: Table S8 — shows the primer sets used in qPCR for titration of AAV genome copy numbers. [file JEM_20230367_TableS8.docx]

Table S8. The primer sets used in qPCR for titration of AAV genome copy number

| Target | ID | Sequences (5’-3’) | Tm (℃) | PCR product (bp) | qPCR condition |
| --- | --- | --- | --- | --- | --- |
| SV40 polyadenylation signal^1)^ | AAV-SV40-R | AGC AAT AGC ATC ACA AAT TTC ACA A | 70 | 96 | initial denaturation step at 95° for 5 min followed by 40 cycles of denaturation at 95°C for 15 s and annealing or extension at 60°C for 30 s |
|  | AAV-SV40-F | GCA GAC ATG ATA AGA TAC ATT GA | 62 |  |  |
| EGFP^2)^ | AAV-EGFP-F | CCACATGAAGCAGCAGGACTT | 60 | 63 | initial denaturation step at 95° for 5 min followed by 40 cycles of denaturation at 95°C for 15 s and annealing or extension at 60°C for 30 s |
|  | AAV-EGFP-R | GGTGCGCTCCTGGACGTA | 60 |  |  |

^1)^ Werling, Natalie Jayne, et al. "Systematic comparison and validation of quantitative real-time PCR methods for the quantitation of adeno-associated viral products." Human gene therapy methods 26.3 (2015): 82-92.

^2)^ Joshi, Molishree U., et al. "Real-time PCR to determine transgene copy number and to quantitate the biolocalization of adoptively transferred cells from EGFP-transgenic mice." Biotechniques 45.3 (2008): 247-258.
