# Supplementary material for: ARL6IP1 gene delivery reduces neuroinflammation and neurodegenerative pathology in hereditary spastic paraplegia model
Source: J Exp Med. 2023 Nov 7;221(1):e20230367. doi: 10.1084/jem.20230367 (PMC10630151; doi:10.1084/jem.20230367)
Supplement: Table S9 — lists R-square, slope, and intersection values of the qPCR. [file JEM_20230367_TableS9.docx]

Table S9. R-square, slope, and intersection values of the qPCR

|  | **EGFP** | | **SV40-poly(A)** | |
| --- | --- | --- | --- | --- |
|  | undigested | BamHI linearized | undigested | BamHI linearized |
| **Ct range** | 9.28-29.02 | 8.32-28.86 | 6.205-29.805 | 6.755-29.85 |
| **R square** | 0.9737 | 0.9703 | 0.9806 | 0.996 |
| **Slope** | -3.016547619 | -3.15 | -3.470178571 | -3.417738095 |
| **Intercept** | 79.927329 | 82.03034 | 87.702082 | 85.347747 |
| **qPCR efficiency (%)** | 114.5382803 | 107.7113926 | 94.16440149 | 96.1512922 |
