# Supplementary material for: ARL6IP1 gene delivery reduces neuroinflammation and neurodegenerative pathology in hereditary spastic paraplegia model
Source: J Exp Med. 2023 Nov 7;221(1):e20230367. doi: 10.1084/jem.20230367 (PMC10630151; doi:10.1084/jem.20230367)
Supplement: Table S10 — lists titration of AAV9 genome copy number using SV40 primer to mouse tissues. [file JEM_20230367_TableS10.docx]

Table S10. Titration of AAV9 genome copy number using SV40 primer to mouse tissues

| **Route of administration** | **AAV serotype** | **tissues** | **AAV copy numbers/gDNA_1mg** | | | |
| --- | --- | --- | --- | --- | --- | --- |
|  |  |  | AAV9-ARL6IP1_Day 7 | | AAV9-ARL6IP1_Day 90 | |
|  |  |  | Run 1 | Run 2 | Run 1 | Run 2 |
| Stereotaxic injection  (Motor cortex) | AAV9-ARL6IP1 | Cortex | 1.359E+05 | 1.272E+05 | 1.587E+05 | 1.282E+05 |
|  |  | Hippocampus | 1.033E+05 | 1.248E+05 | 2.459E+04 | 5.625E+04 |
|  |  | Midbrain | 7.955E+04 | 1.190E+05 | 1.523E+04 | 4.983E+04 |
|  |  | Cerebellum | 8.464E+04 | 6.925E+04 | ND | 3.945E+04 |
|  |  | Spinal cord | 3.373E+04 | 8.713E+04 | ND | ND |

| **Route of administration** | **AAV serotype** | **tissues** | **AAV copy numbers/gDNA_1mg** | | | |
| --- | --- | --- | --- | --- | --- | --- |
|  |  |  | AAV9-ARL6IP1_Day 7 | | AAV9-ARL6IP1_Day 90 | |
|  |  |  | Run 1 | Run 2 | Run 1 | Run 2 |
| Stereotaxic injection  (Motor cortex) | AAV9-ARL6IP1 | Testes | ND | ND | 1.215E+03 | ND |
|  |  | Spleen | ND | ND | 6.209E+02 | 2.098E+03 |

* LLOQ (lower limit of quantification) : <10^3^ copies/1mg genomic DNA
