# Supplementary figures and images for: ARL6IP1 gene delivery reduces neuroinflammation and neurodegenerative pathology in hereditary spastic paraplegia model
Source: J Exp Med. 2023 Nov 7;221(1):e20230367. doi: 10.1084/jem.20230367 (PMC10630151; doi:10.1084/jem.20230367)

SourceData F2D

Spinal cord tissues

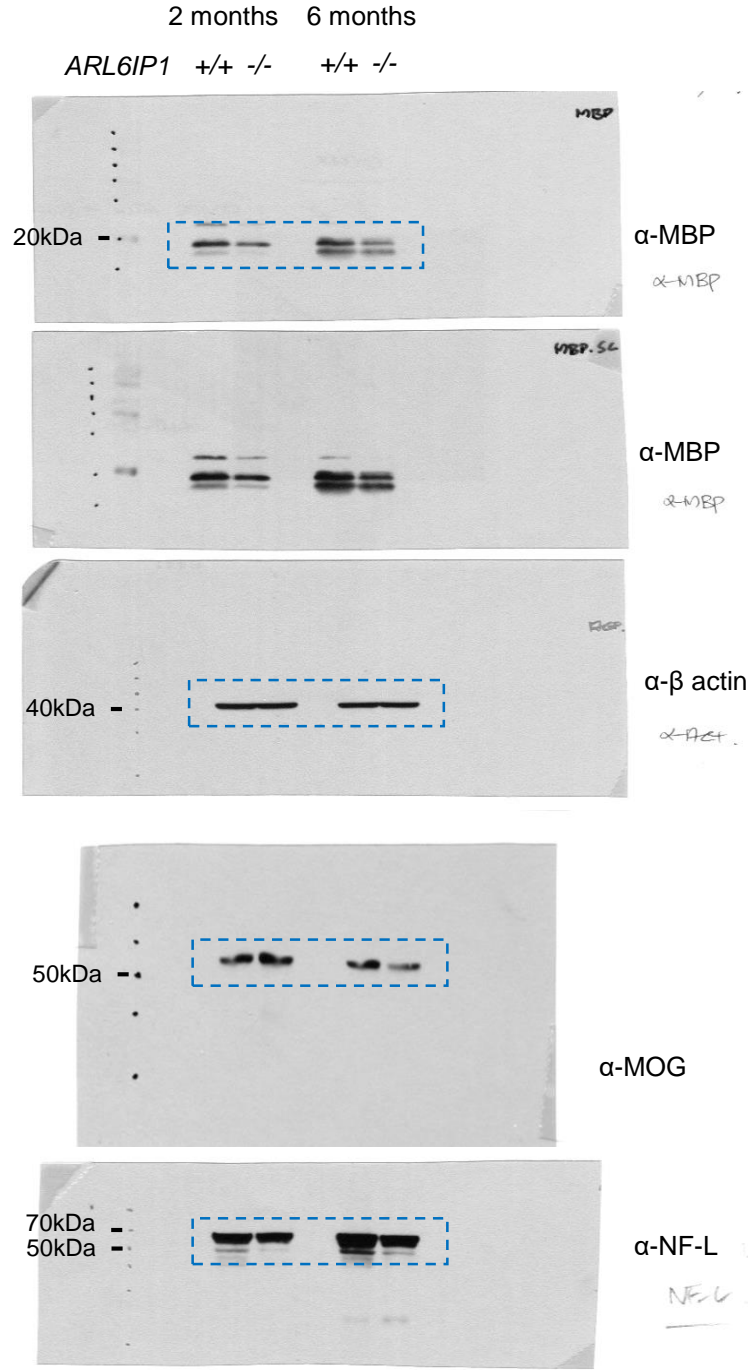

Supplement: SourceData F2 — is the source file for Fig. 2. [file JEM_20230367_SourceDataF2.pdf]

SourceData F3F

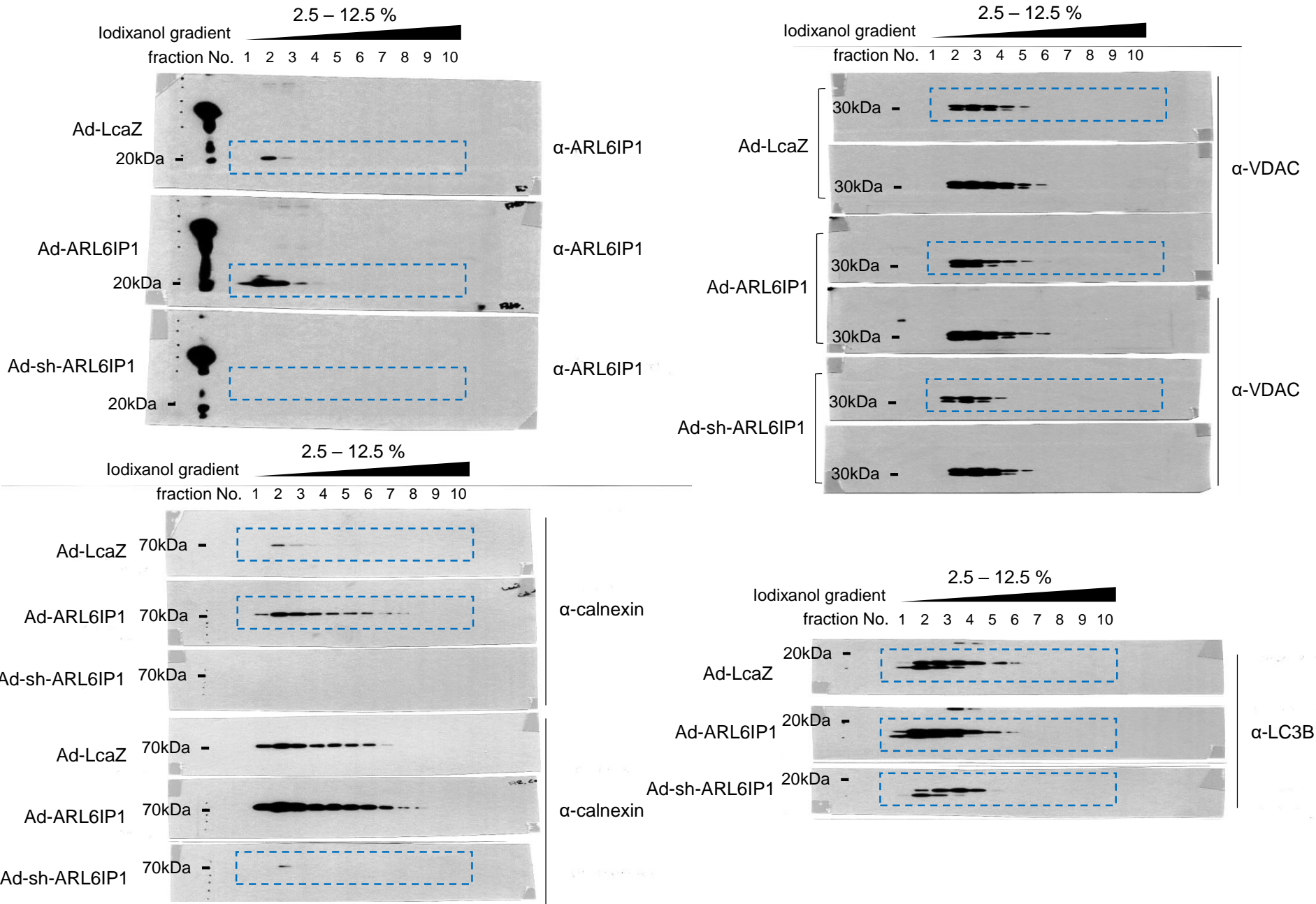

SourceData F3I

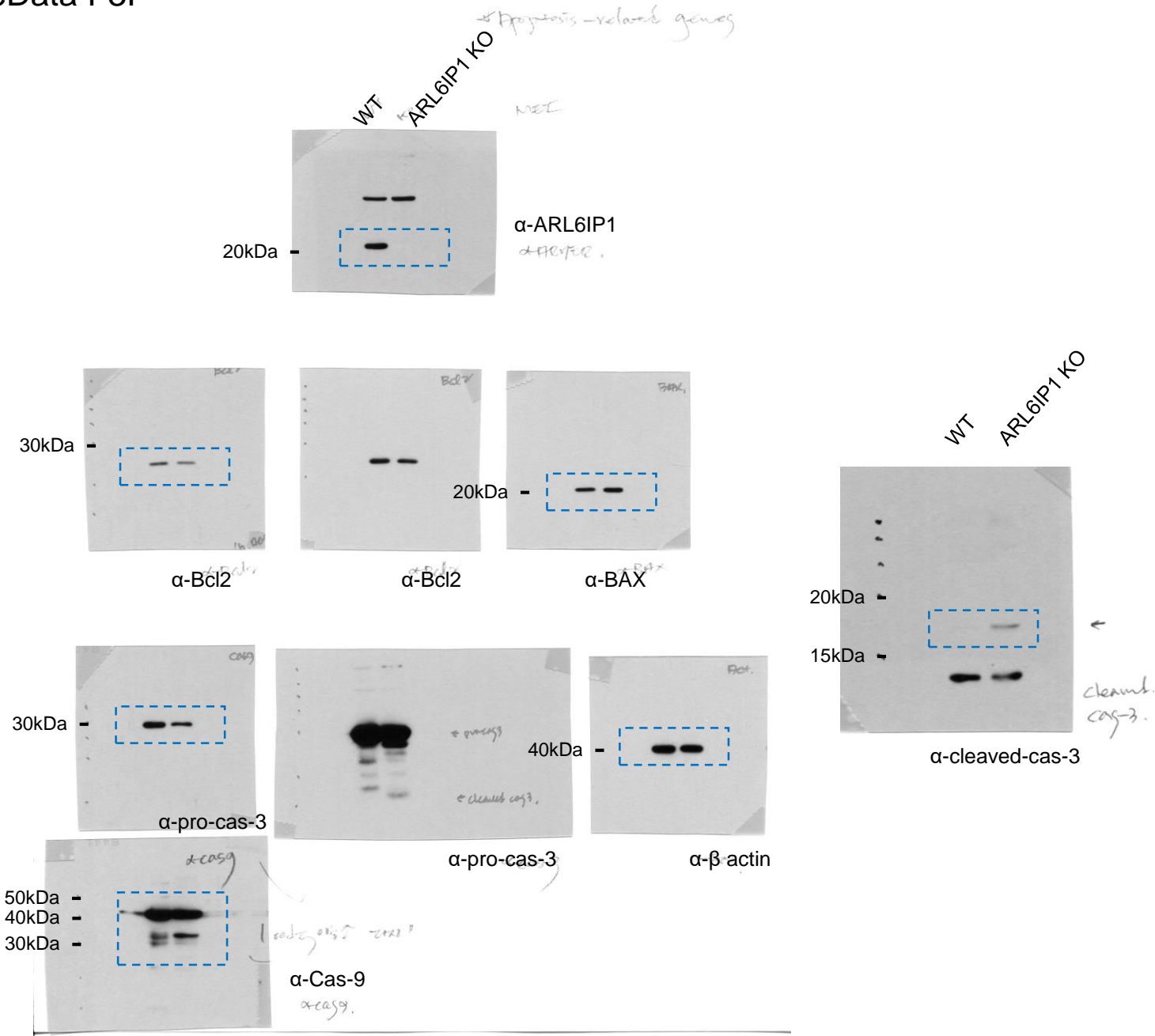

Supplement: SourceData F3 — is the source file for Fig. 3. [file JEM_20230367_SourceDataF3.pdf]

SourceData F7B

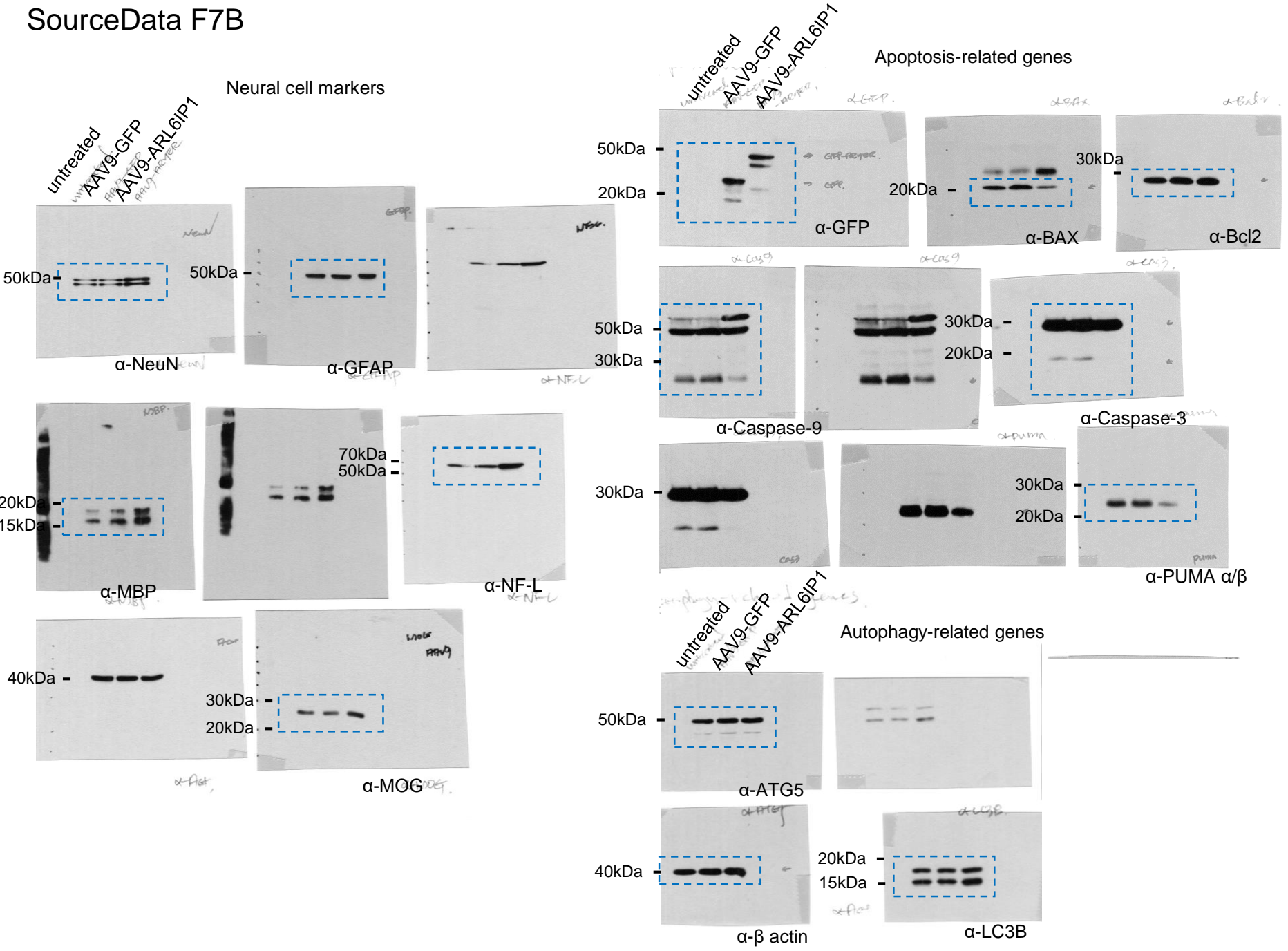

Supplement: SourceData F7 — is the source file for Fig. 7. [file JEM_20230367_SourceDataF7.pdf]

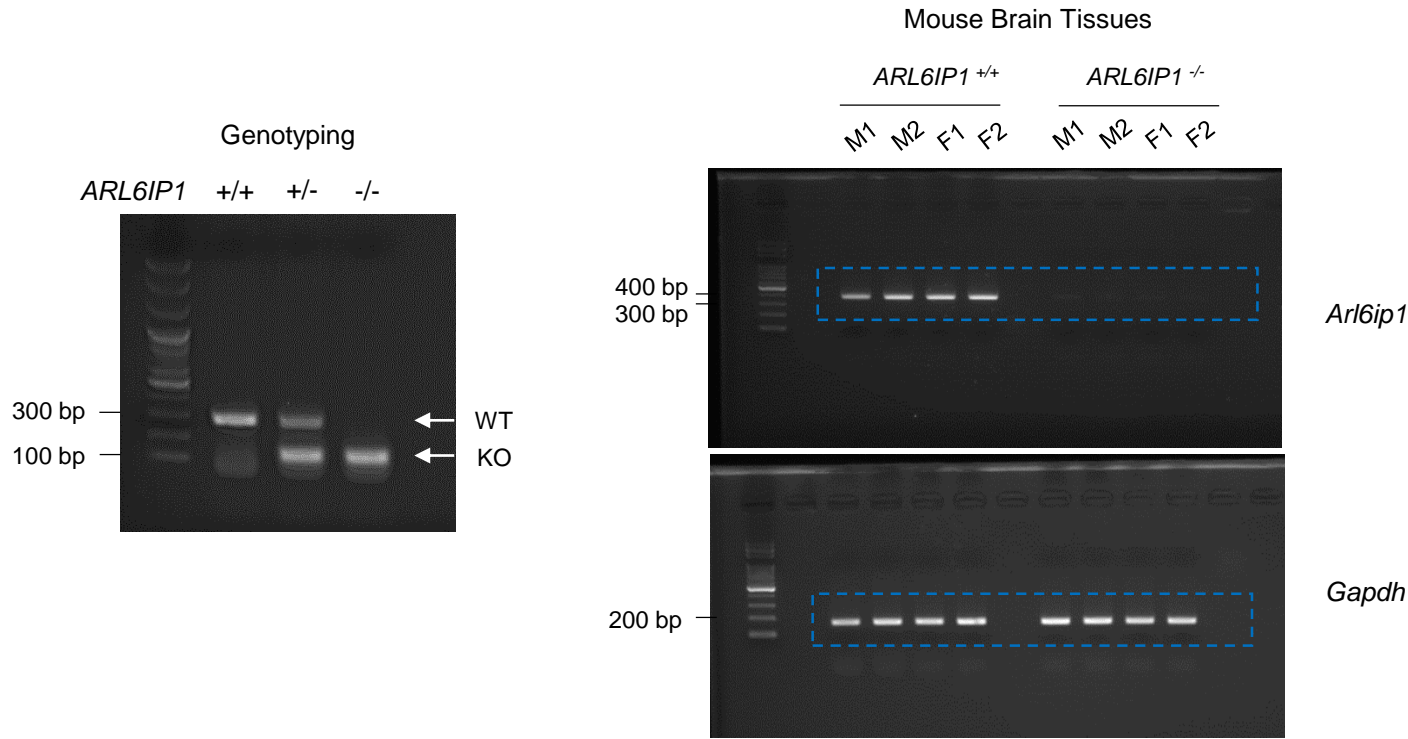

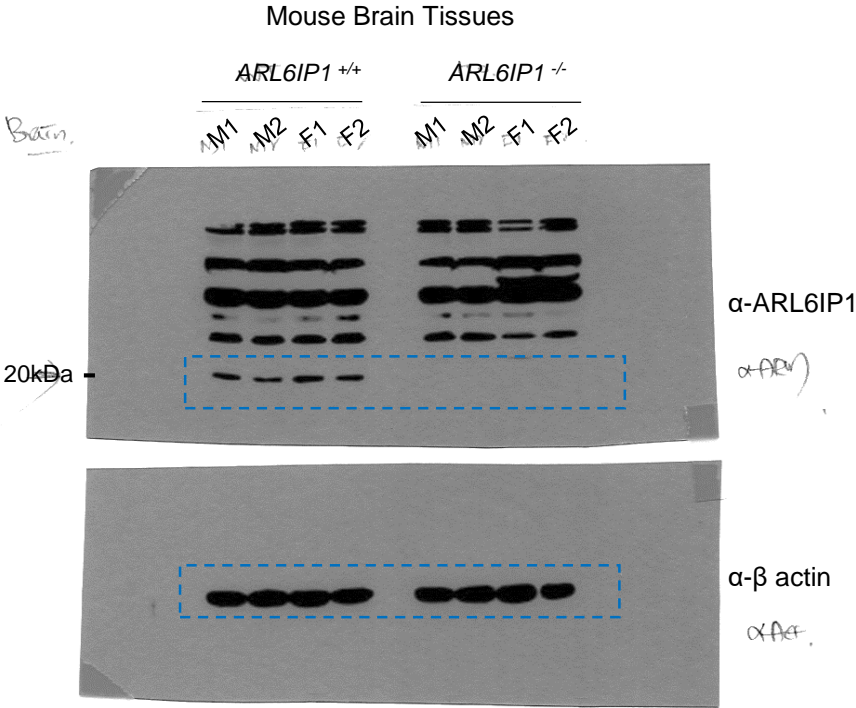

Supplement: SourceData FS1 — is the source file for Fig. S1. [file JEM_20230367_SourceDataFS1.pdf]

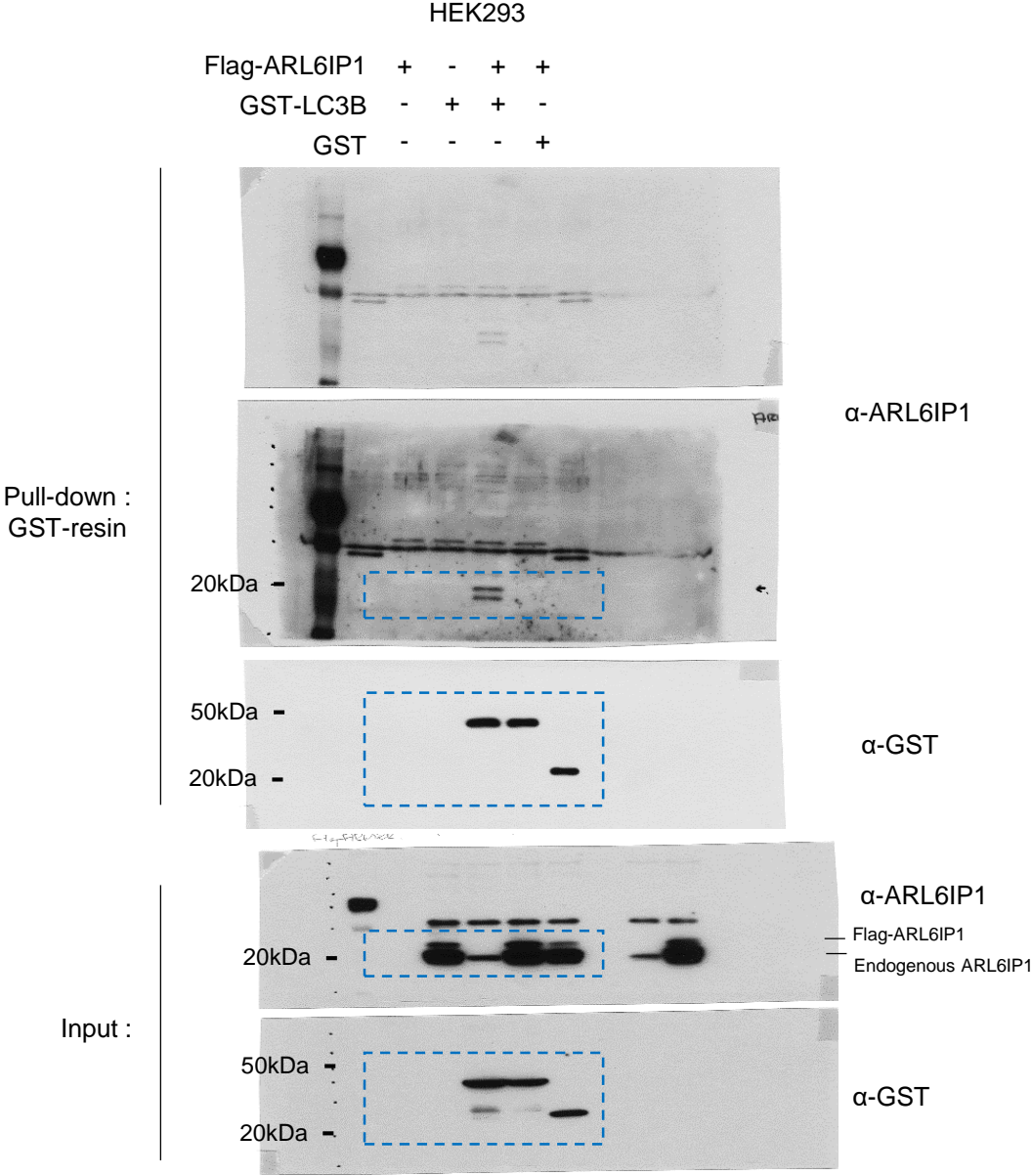

In vitro Binding assay

|             |   |   |   |    |   |
|-------------|---|---|---|----|---|
| His-ARL6IP1 | + | - | + | +  | + |
| GST-LC3B    | - | + | + | ++ | - |
| GST         | - | - | - | -  | + |

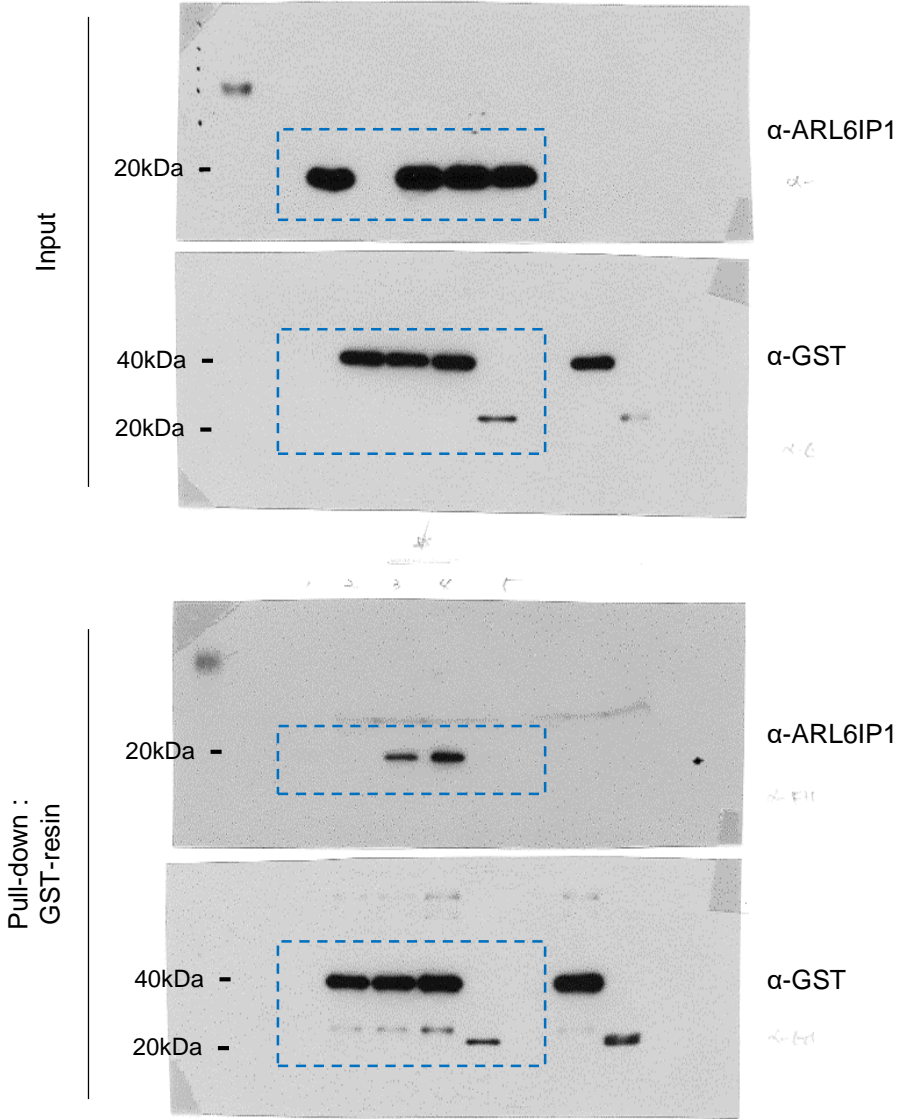

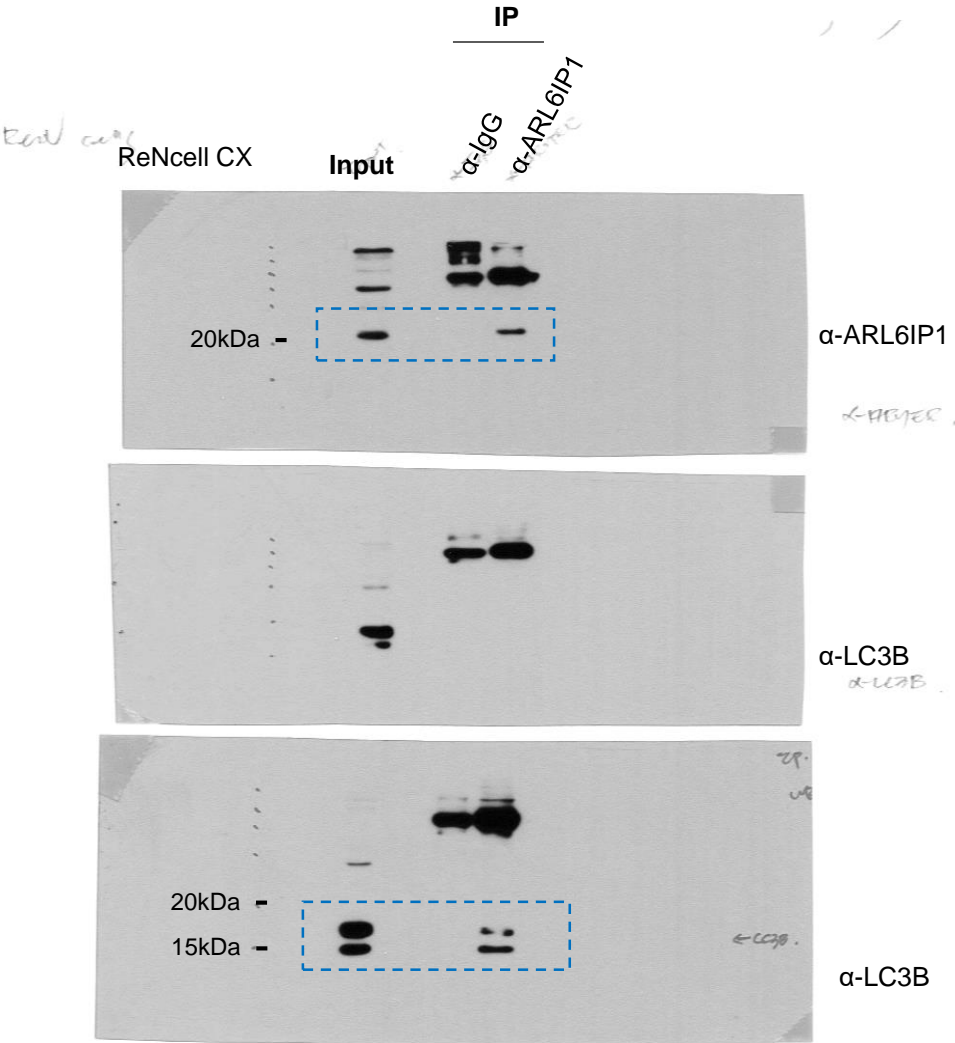

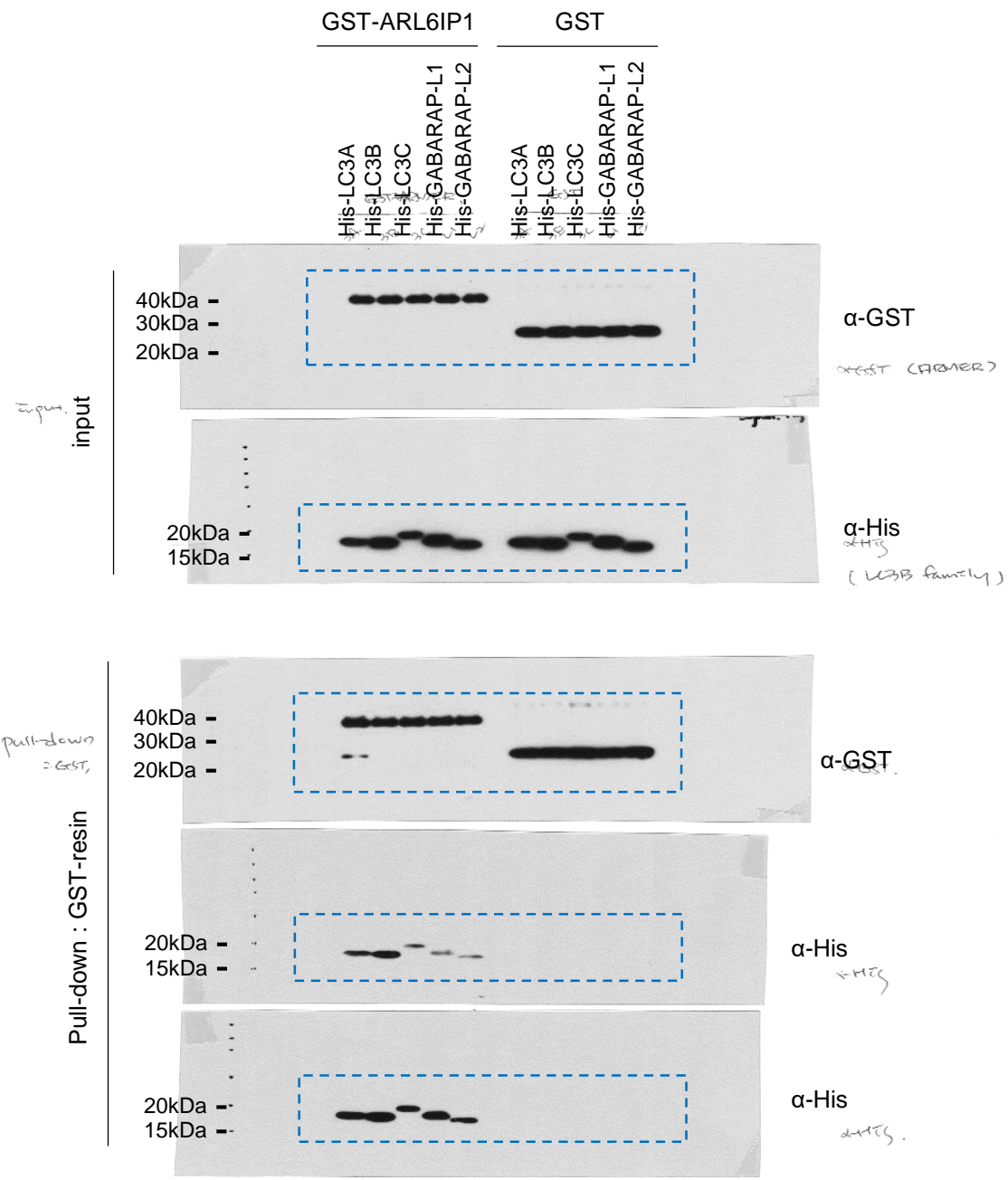

Supplement: SourceData FS3 — is the source file for Fig. S3. [file JEM_20230367_SourceDataFS3.pdf]

SourceData FS4E

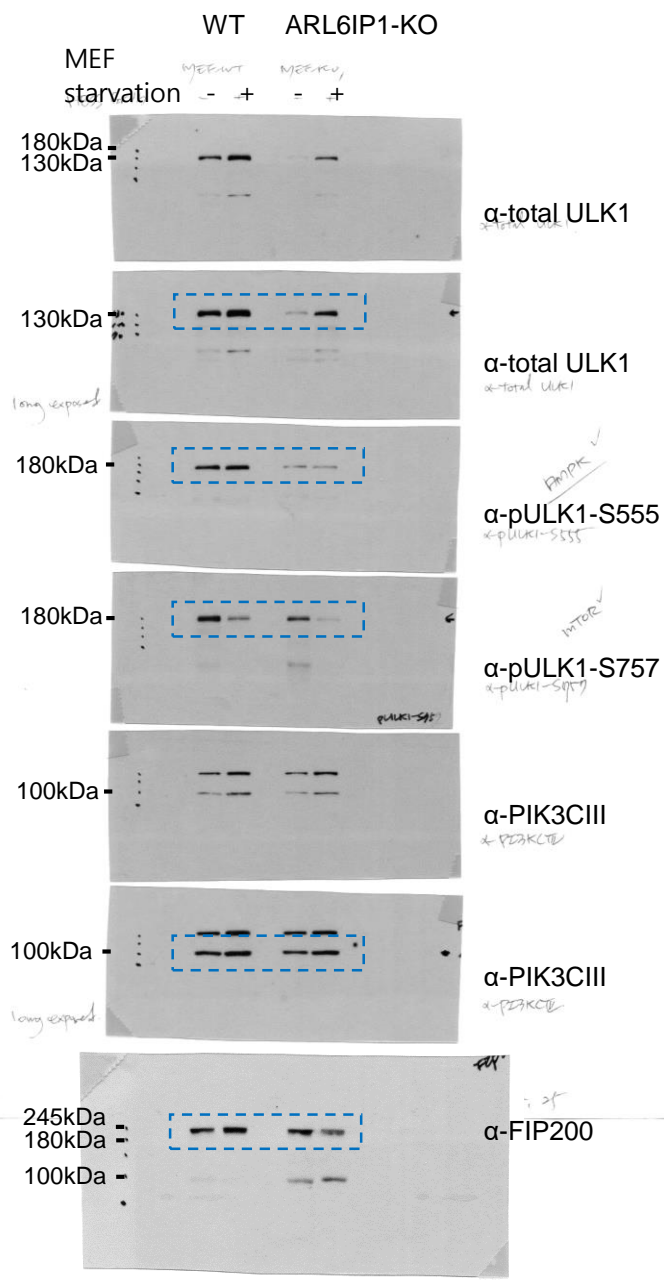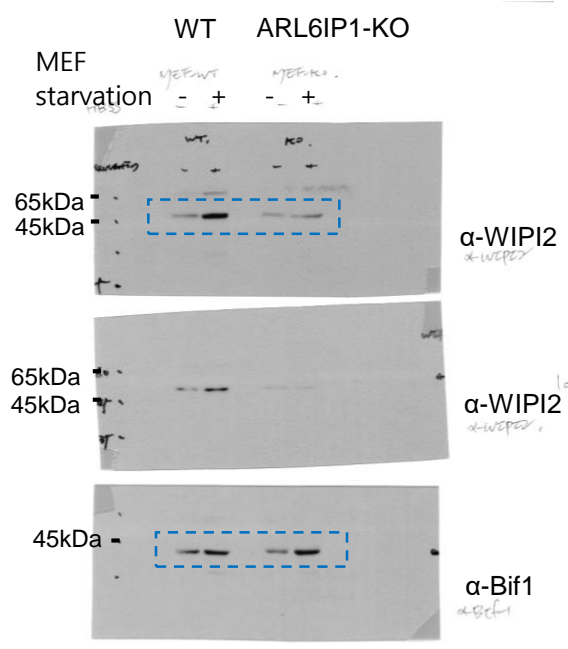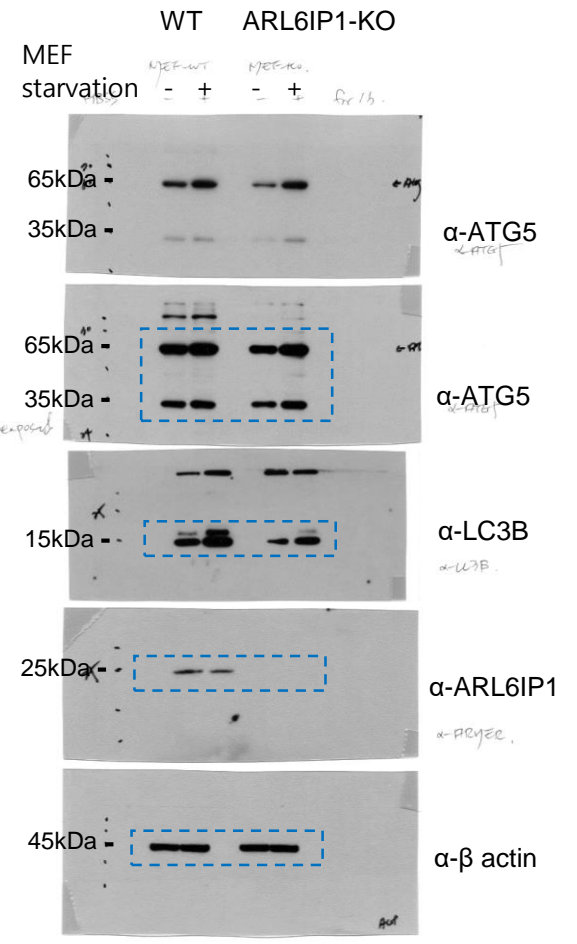

Supplement: SourceData FS4 — is the source file for Fig. S4. [file JEM_20230367_SourceDataFS4.pdf]

DNA ladder  
pAAV-ARL6IP1  
undigested  
pAAV-ARL6IP1  
BamHI linearized

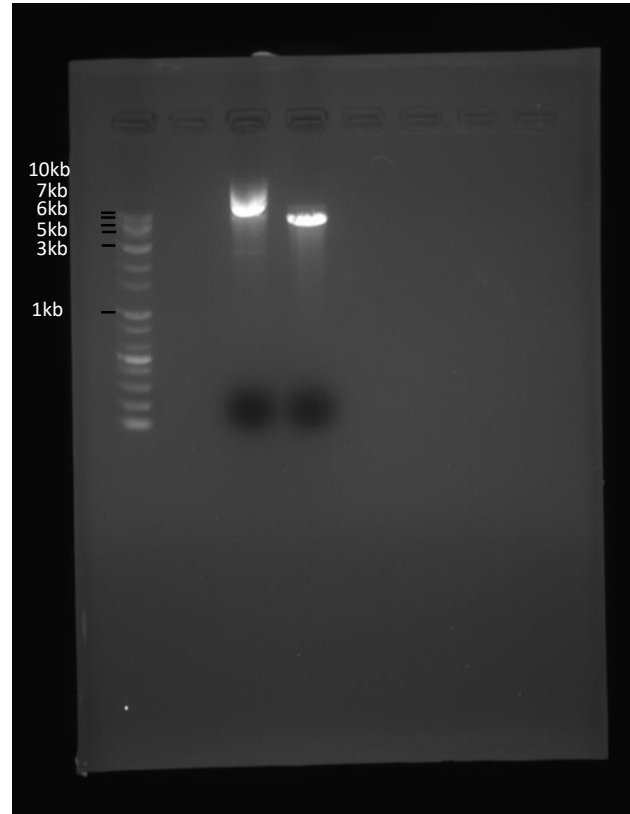

Supplement: SourceData FS5 — is the source file for Fig. S5. [file JEM_20230367_SourceDataFS5.pdf]
