# Supplementary material for: ARL6IP1 gene delivery reduces neuroinflammation and neurodegenerative pathology in hereditary spastic paraplegia model
Source: J Exp Med. 2023 Nov 7;221(1):e20230367. doi: 10.1084/jem.20230367 (PMC10630151; doi:10.1084/jem.20230367)

SourceData F4B

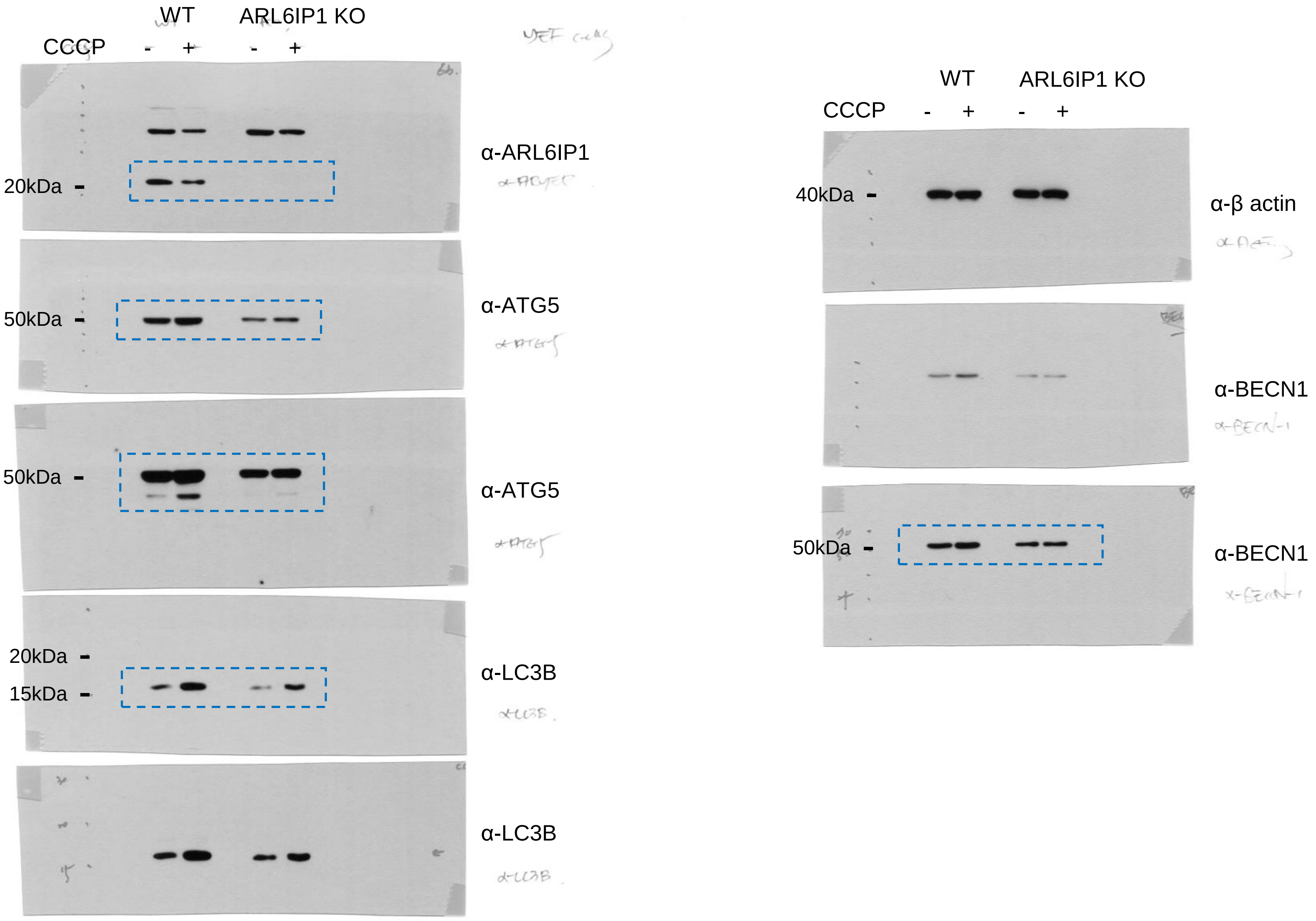

SourceData F4D

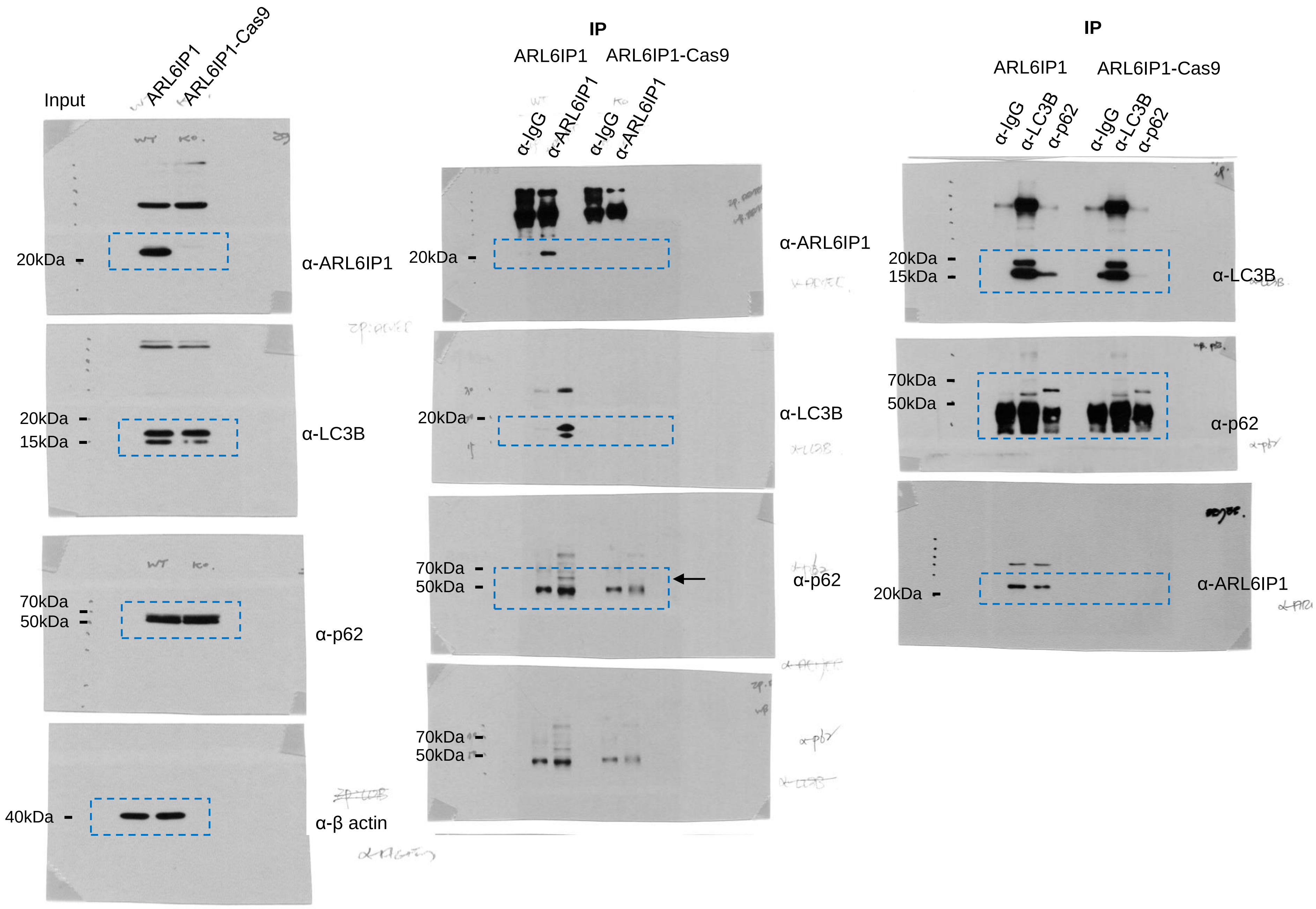

SourceData F4E

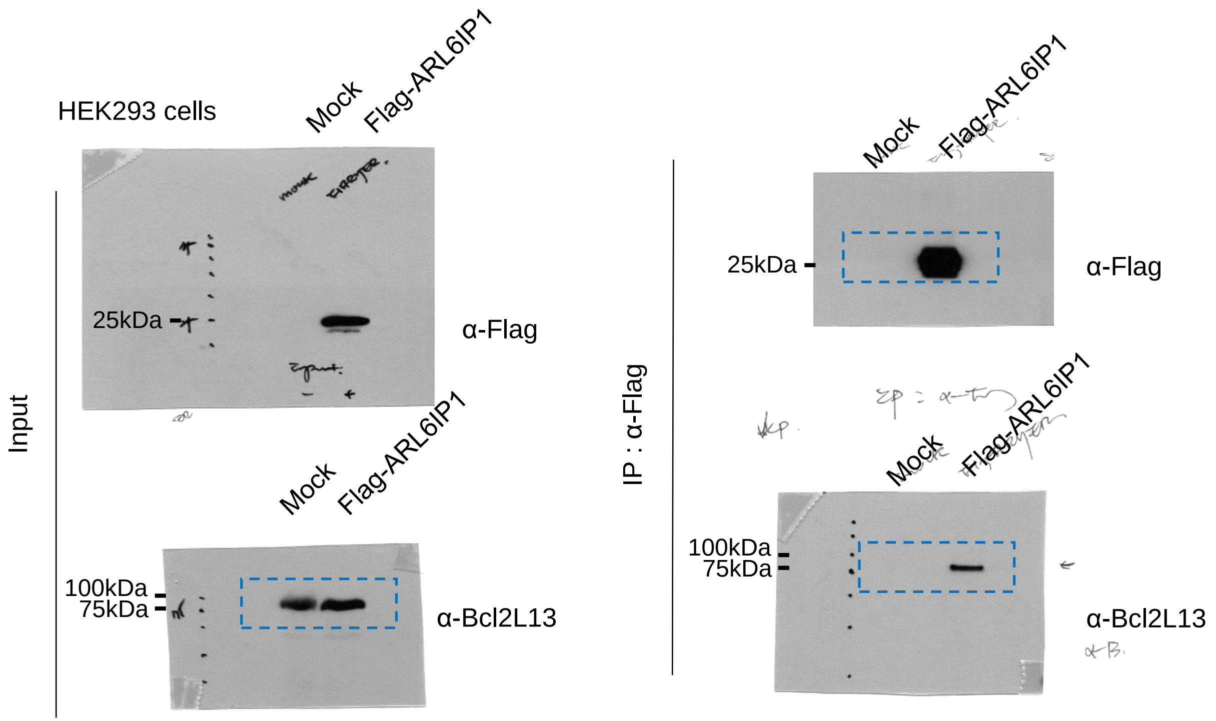

SourceData F4F (continued)

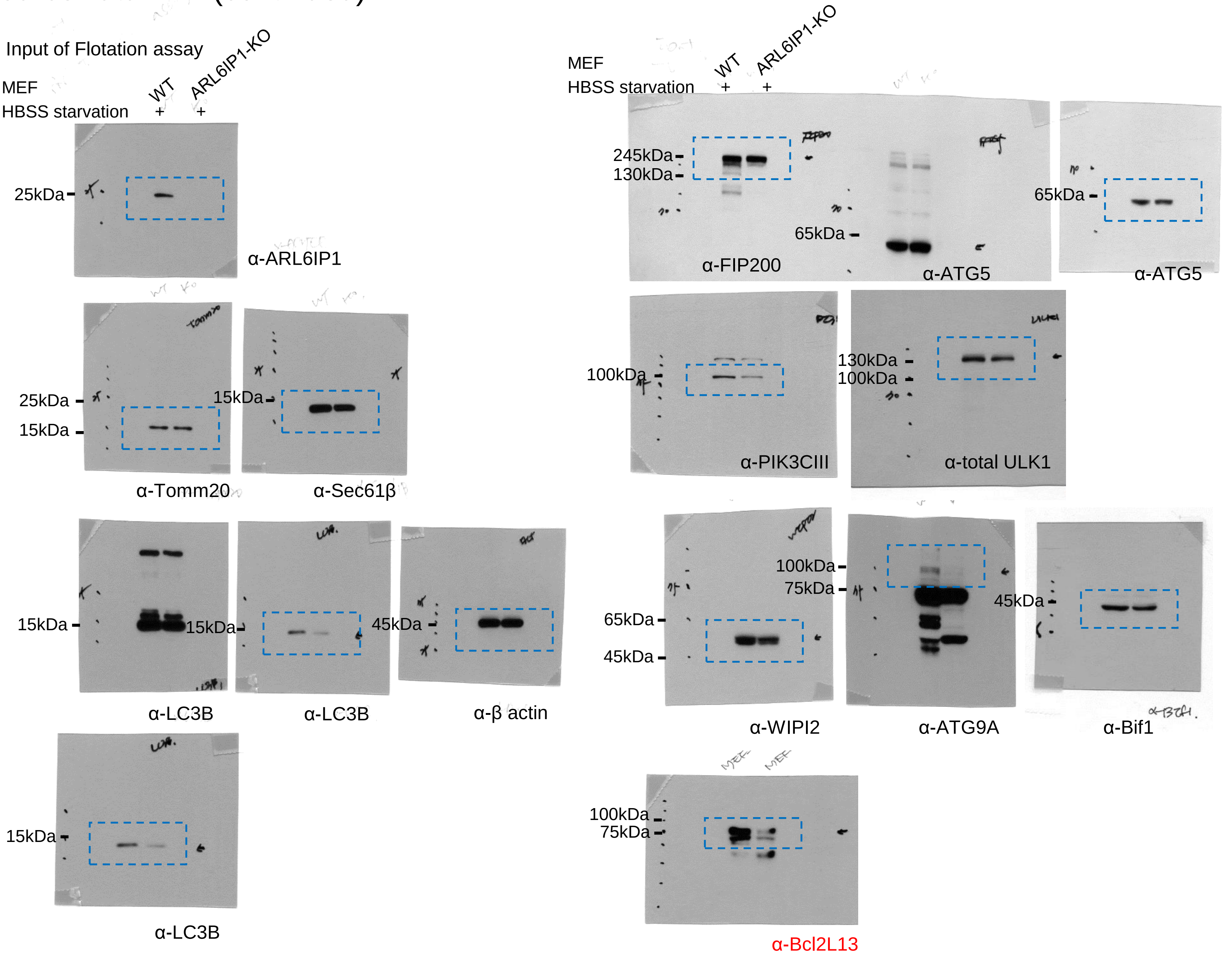

SourceData F4F (continued)

Flotation assay in MEF-WT cells

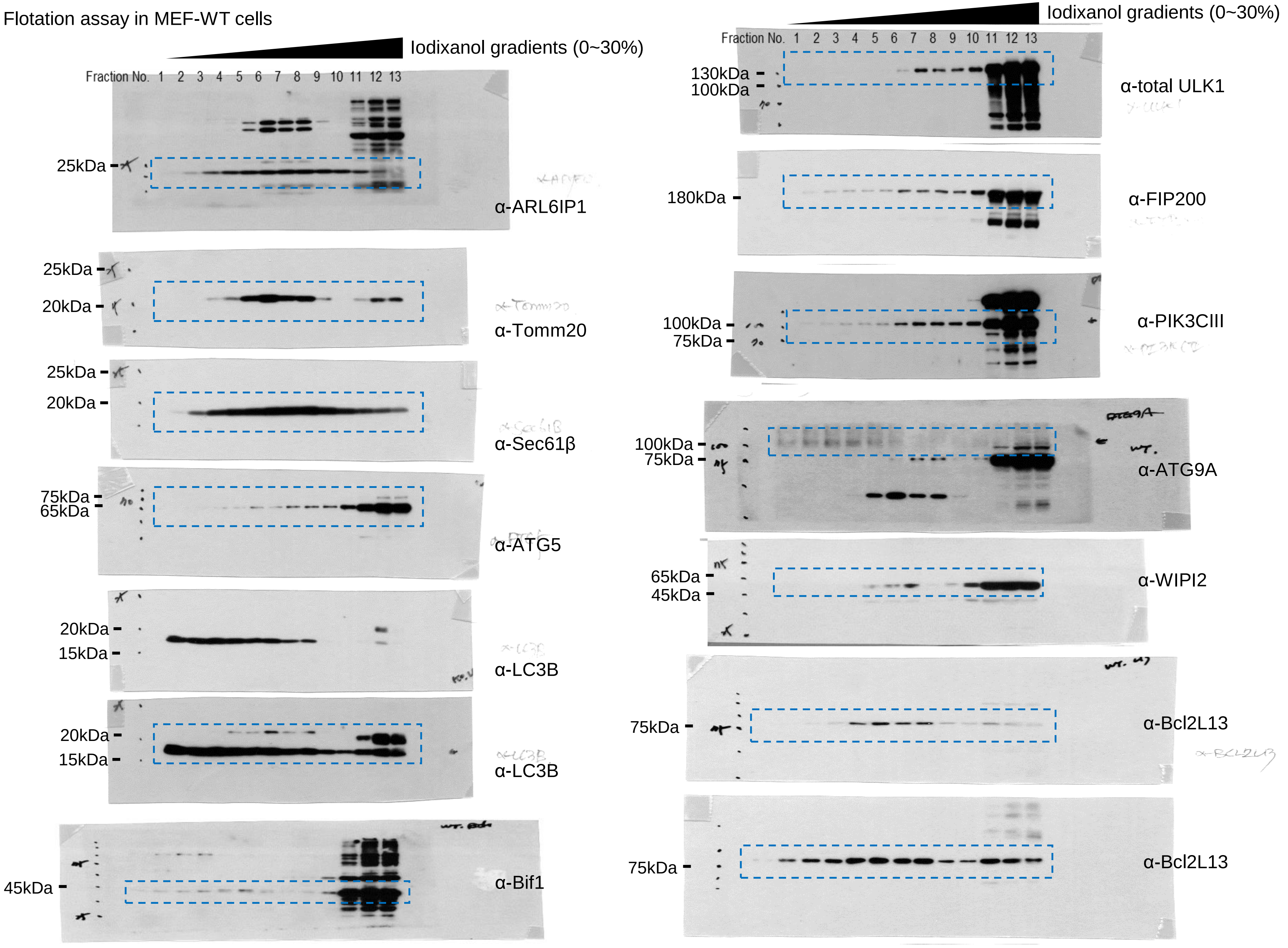

SourceData F4F (continued)

Flotation assay in MEF-KO cells

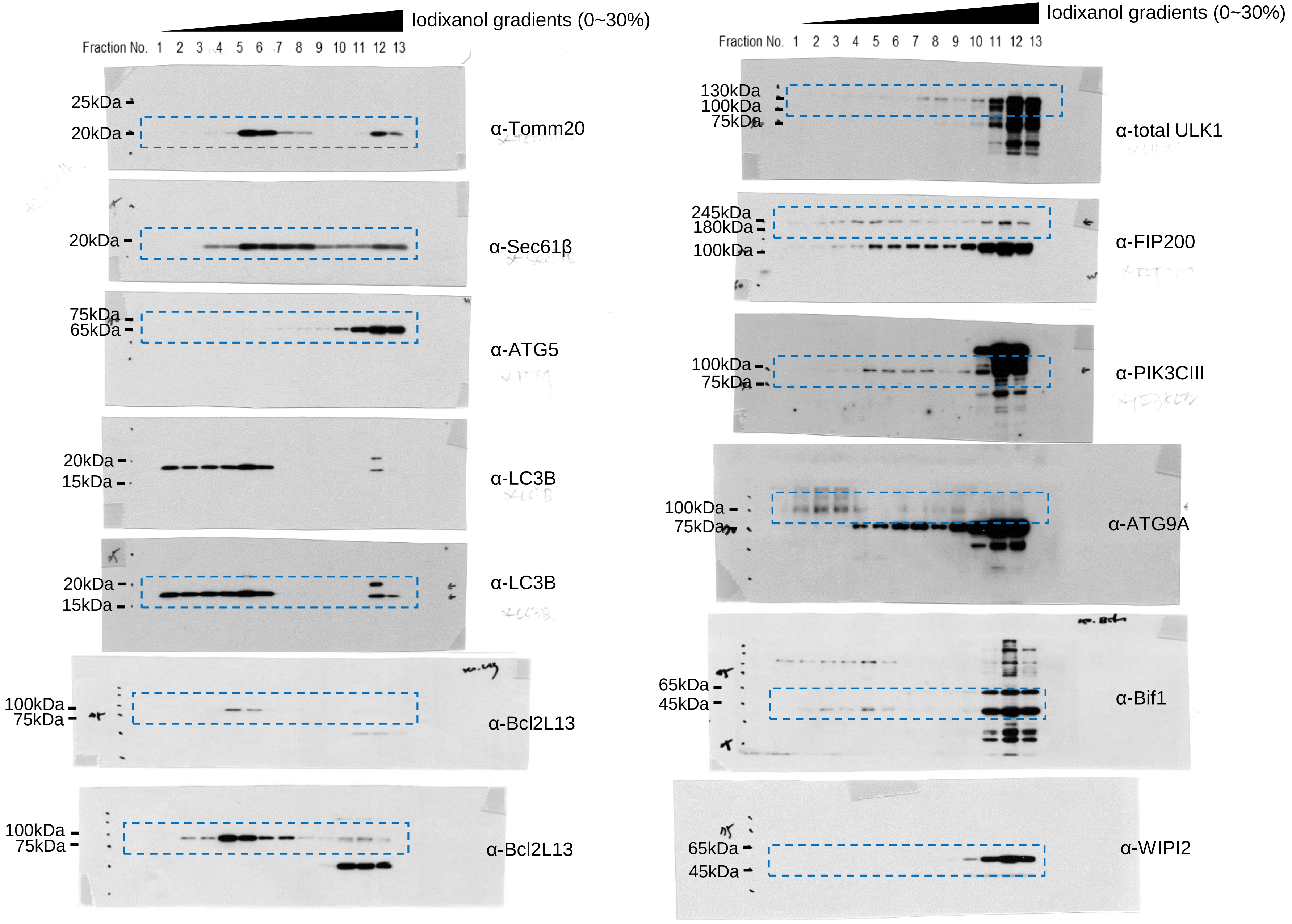

Supplement: SourceData F4 — is the source file for Fig. 4. [file JEM_20230367_SourceDataF4.pdf]
