# Supplementary material for: ARL6IP1 gene delivery reduces neuroinflammation and neurodegenerative pathology in hereditary spastic paraplegia model
Source: J Exp Med. 2023 Nov 7;221(1):e20230367. doi: 10.1084/jem.20230367 (PMC10630151; doi:10.1084/jem.20230367)

SourceData FS2E

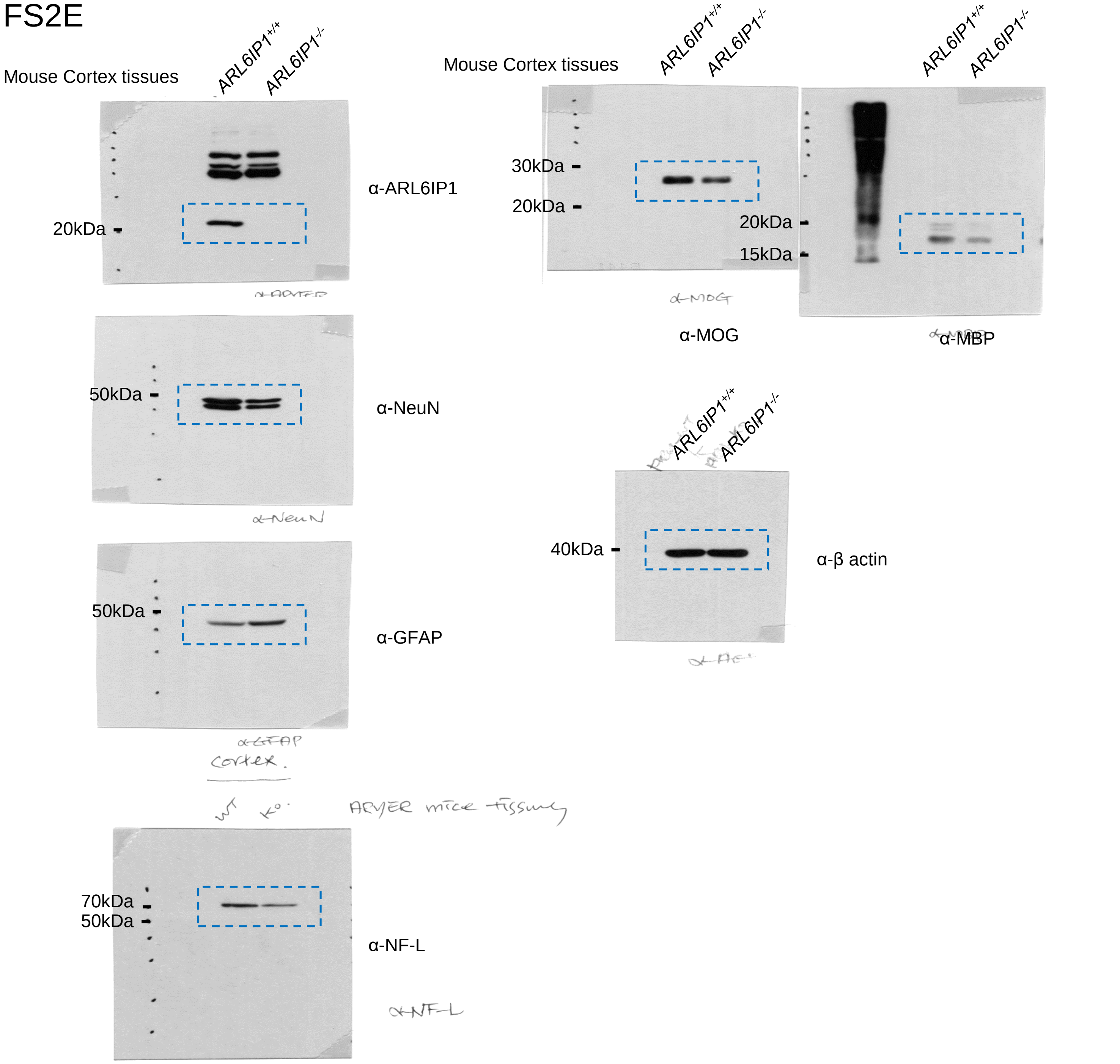

Supplement: SourceData FS2 — is the source file for Fig. S2. [file JEM_20230367_SourceDataFS2.pdf]
